# Digital interventions to reduce social isolation and loneliness in older adults: An evidence and gap map

**DOI:** 10.1002/cl2.1369

**Published:** 2023-11-27

**Authors:** Vivian Welch, Elizabeth T. Ghogomu, Victoria I. Barbeau, Sierra Dowling, Rebecca Doyle, Ella Beveridge, Elisabeth Boulton, Payaam Desai, Jimmy Huang, Nour Elmestekawy, Tarannum Hussain, Arpana Wadhwani, Sabrina Boutin, Niobe Haitas, Dylan Kneale, Douglas M. Salzwedel, Roger Simard, Paul Hébert, Christopher Mikton

**Affiliations:** ^1^ Methods Centre, Bruyère Research Institute Ottawa Ontario Canada; ^2^ Bruyère Research Institute University of Ottawa Ottawa Ontario Canada; ^3^ Lakehead University Thunder Bay Ontario Canada; ^4^ School of Health Sciences, Division of Nursing, Midwifery and Social Work University of Manchester Manchester UK; ^5^ Canadian Red Cross Montreal Quebec Canada; ^6^ Social Science Research Unit, EPPI‐Centre, UCL Institute of Education University College London London UK; ^7^ Department of Anesthesiology, Pharmacology and Therapeutics University of British Columbia Vancouver British Columbia Canada; ^8^ World Health Organization Geneva Switzerland

## Abstract

**Background:**

Social isolation and loneliness are more common in older adults and are associated with a serious impact on their well‐being, mental health, physical health, and longevity. They are a public health concern highlighted by the COVID‐19 pandemic restrictions, hence the need for digital technology tools to enable remotely delivered interventions to alleviate the impact of social isolation and loneliness during the COVID‐19 restrictions.

**Objectives:**

To map available evidence on the effects of digital interventions to mitigate social isolation and/or loneliness in older adults in all settings except hospital settings.

**Search Methods:**

We searched the following databases from inception to May 16, 2021, with no language restrictions. Ovid MEDLINE, Embase, APA PsycInfo via Ovid, CINAHL via EBSCO, Web of Science via Clarivate, ProQuest (all databases), International Bibliography of the Social Sciences (IBSS) via ProQuest, EBSCO (all databases except CINAHL), Global Index Medicus, and Epistemonikos.

**Selection Criteria:**

Titles and abstracts and full text of potentially eligible articles were independently screened in duplicate following the eligibility criteria.

**Data Collection and Analysis:**

We developed and pilot tested a data extraction code set in Eppi‐Reviewer and data were individually extracted and coded based on an intervention‐outcome framework which was also used to define the dimensions of the evidence and gap map.

**Main Results:**

We included 200 articles (103 primary studies and 97 systematic reviews) that assessed the effects of digital interventions to reduce social isolation and/or loneliness in older adults. Most of the systematic reviews (72%) were classified as critically low quality, only 2% as high quality and 25% were published since the COVID‐19 pandemic. The evidence is unevenly distributed with clusters predominantly in high‐income countries and none in low‐income countries. The most common interventions identified are digital interventions to enhance social interactions with family and friends and the community via videoconferencing and telephone calls. Digital interventions to enhance social support, particularly socially assistive robots, and virtual pets were also common. Most interventions focused on reducing loneliness and depression and improving quality of life of older adults. Major gaps were identified in community level outcomes and process indicators. No included studies or reviews assessed affordability or digital divide although the value of accessibility and barriers caused by digital divide were discussed in three primary studies and three reviews. Adverse effects were reported in only two studies and six reviews. No study or review included participants from the LGBTQIA2S+ community and only one study restricted participants to 80 years and older. Very few described how at‐risk populations were recruited or conducted any equity analysis to assess differences in effects for populations experiencing inequities across PROGRESS‐Plus categories.

**Authors' Conclusions:**

The restrictions placed on people during the pandemic have shone a spotlight onto social isolation and loneliness, particularly for older adults. This evidence and gap map shows available evidence on the effectiveness of digital interventions for reducing social isolation or loneliness in older adults. Although the evidence is relatively large and recent, it is unevenly distributed and there is need for more high‐quality research. This map can guide researchers and funders to consider areas of major gaps as priorities for further research.

## 1 PLAIN LANGUAGE SUMMARY

### 1.1 Evidence for digital interventions to reduce social isolation and loneliness in older adults is unevenly distributed and most existing reviews are of critically low quality

Mapping the evidence for digital interventions to reduce social isolation and loneliness in older adults shows that many of the systematic reviews are of critically low quality. Most come from high‐income countries, with sparse reporting of community‐level outcomes.

### 1.2 What is this EGM about?

Social isolation and loneliness are common in older adults and have been highlighted during the COVID‐19 pandemic. Social isolation and loneliness pose a significant concern because of their impact on older adults' well‐being, mental health, physical health and longevity.

Older adults can use digital interventions to maintain existing connections or develop new connections. This was especially evident during the COVID‐19 pandemic, with social distancing and lockdown measures in place. Decisionmakers need to know which digital interventions can reduce social isolation and loneliness in older adults.

### 1.3 What is the aim of this EGM?

This EGM aims to show available evidence from systematic reviews and primary studies on the effects of digital interventions to reduce social isolation and/or loneliness among older adults in all settings except hospital settings.

### 1.4 What studies are included?

The EGM includes 200 articles (97 systematic reviews and 103 primary studies) that assessed how digital interventions can reduce social isolation and loneliness in older adults. The studies had to report the effect of digital interventions and could come from any region.

### 1.5 What are the main findings of this gap map?

The evidence is unevenly distributed geographically, with most from high‐income countries and none from low‐income countries. Over 70% of the systematic reviews have critically low quality and 25% have been published since the pandemic began.

The most common interventions are digital interventions to enhance social interactions with family, friends and the community via videoconferencing and telephone calls. Digital interventions to enhance social support, particularly socially assistive robots and virtual pets, were also common.

Most interventions focus on reducing loneliness and depression and improving quality of life of older adults.

Community level outcomes and process indicators are hardly reported, and no included studies or reviews assess affordability or lack of accessibility, although the value of accessibility and barriers caused by lack of accessibility were discussed in three studies and three reviews. Adverse effects are reported in very few studies and reviews.

Participants from the LGBTQIA2S+ community are not included in any study or review and only one study restricted participants to 80 years and older. Very few studies or reviews describe how at‐risk populations were recruited or conduct any equity analysis to assess differential effects for populations experiencing inequities across PROGRESS‐Plus factors.

### 1.6 What do the findings of the map mean?

This map is the first step towards identifying which digital interventions are effective for reducing social isolation and loneliness in older adults. The EGM contributes to the longer‐term aim of building an evidence architecture for the field, whereby the use of evidence becomes institutionalized in policy and practice.

Although the evidence is relatively large and recent, it is unevenly distributed and there is need for more high‐quality research. This map can guide researchers and funders to consider areas of major gaps as priorities for further research.

### 1.7 How up‐to‐date is this EGM?

The authors searched for studies published up to May 2021.

## 2 BACKGROUND

### 2.1 Introduction

#### 2.1.1 The problem, condition or issue

A large body of research shows that social isolation and loneliness are associated with a serious impact on older adult's well‐being, mental health, physical health, and longevity (Leigh‐Hunt, 2017; Menec, 2020). Their effect on mortality is comparable to, or even greater, than other well‐established risk factors such as smoking, obesity, and physical inactivity (Holt‐Lunstad, 2015; Ibarra, 2020; Menec, 2020; Windle, 2012).

Social isolation and loneliness are more common in older adults and are described as multidimensional concepts with different methods of measurement leading to variations in the prevalence. The prevalence ranges from 5% to 43% depending on the study and region (Chen, 2016; Donovan, 2020; Ibarra, 2020; Leigh‐Hunt, 2017). Risk factors include living alone, impaired mobility, experiencing a major life transition change (e.g., loss of spouse or other primary network members), limited income or resources, cognitive impairment, inadequate social support, and geographic location (Cohen‐Mansfield, 2015; Donovan, 2020; Findlay, 2003; Ibarra, 2020).

Although they are related, social isolation and loneliness are two distinct concepts and one may occur without the other. Social isolation is the objective state of lack of interactions with others and the wider community or lack of social relationships (Donovan, 2020; Ibarra, 2020; Leigh‐Hunt, 2017; Menec, 2020). Loneliness is the subjective painful feeling of the absence of a social network or a companion or perception of unmet emotional and social needs resulting from a mismatch between the desired and actual experience of the quality or quantity of social relationships (Cacioppo, 2009; Cacioppo, 2014; Menec, 2020; Perlman, 1981; Prohaska, 2020; WHO, 2021). Therefore, an individual can have a social network and be lonely or a socially isolated individual may not feel lonely. An understanding of the differences in these concepts is important for research in the development of appropriate and effective interventions, and standardizing outcome measurements and also to guide the choice of appropriate interventions for socially isolated or lonely individuals (Fakoya, 2020; WHO, 2021).

Social isolation and loneliness among older adults are priority public health problems, as well as national and international policy issues, due to the negative impact on their mental and physical health and longevity (Cattan, 2005; Gardiner, 2018; Shah, 2020; Shah, 2021; WHO, 2020; WHO, 2021). The World Health Organization (WHO) decided, as part of the Decade of Healthy Ageing, to address social isolation and loneliness as a priority issue that cuts across the main action areas of the Decade (WHO, 2020). It is also increasingly being recognized as a public health concern due to the social distancing measures during the COVID‐19 pandemic (Brooke, 2020; Shah, 2020; Shah, 2021; Williams, 2021). For example, the average person's daily number of contacts was reduced by up to 74% and almost one quarter of adults in the UK experienced loneliness when living under lockdown (Williams, 2021). Hence the need for digital technology tools to enable remotely delivered interventions to alleviate the impact of social isolation and loneliness during the COVID‐19 restrictions.

There are challenges associated with access to digital interventions and the use of remotely delivered interventions to reduce social isolation and loneliness. Disparities in access to digital interventions and the use of remotely delivered interventions is a growing concern, especially for older adults and during the COVID‐19 restrictions (Budd, 2020; Jopling, 2020; Watts, 2020; Williams, 2021). Many older adults lack digital skills and the confidence to access online services and support. Other barriers are affordability and accessibility of technology, broadband or Wi‐Fi, data poverty (i.e., lack of accessibility to wireless internet connection), geographic divide (rural and urban, high income and low‐ and middle‐income countries). Concerns with digital technology use have also been raised regarding privacy invasion, legal, ethical and clinical data governance through data sharing and access to information (Budd, 2020; NASEM, 2020). Ethical concerns with any intervention to prevent social isolation and loneliness include issues with accessibility, acceptability, cost, feasibility, autonomy, and informed concern. Privacy concerns may intersect with accessibility issues due to cognitive impairment or involvement of other family members or caregivers. Informed consent provided at the beginning of research may not cover the future use of data created, raising issues with ongoing consent and the ability to withdraw consent. There is also the risk of digital technology exacerbating rather than reducing social isolation, for example, social robots replacing meaningful human contact can result in increased sense of loneliness, deception and infantilization of older adults (NASEM, 2020). Equitable access and support are key in addressing the digital divide.

#### 2.1.2 The intervention

A wide variety of interventions have been developed to reduce social isolation or loneliness among older adults. These interventions use different strategies and target different aspects such as facilitating social connections or service provision. They are implemented at different levels such as one‐on‐one or group focused. Although several systematic reviews have evaluated the effectiveness of different types of interventions for social isolation and loneliness in older adults, their findings have sometimes been conflicting (Cattan, 2005; Cohen‐Mansfield, 2015; Dickens, 2011; Findlay, 2003; Gardiner, 2018; Hagan, 2014; Victor, 2018).

Digital interventions have become a particular focus of interest, due partly to the social distancing and lock‐down measures introduced to combat the COVID‐19 pandemic and to the rapidly increasing role technology – particularly the internet, mobile devices, social media and Internet of things (IoT) – has played in the last 10–15 years in mediating social relations (Boulton, 2020; Brooke, 2020; Budd, 2020; Falk, 2021; Shah, 2020; UCLG, 2020; WHO, 2021; Zanella, 2020). They have been used in different sectors (e.g., health care, social services, the community) and in various ways, including digital epidemiological surveillance, rapid case identification, interruption of community transmission, public communication, and provision of clinical care and income support and livelihood opportunities in the COVID‐19 crisis.

Digital interventions have also been used to mitigate social isolation or loneliness in older adults by facilitating social interaction or by delivering programs or services (Boulton, 2020; Chen, 2016; Chipps, 2017; Findlay, 2003; Ibarra, 2020; Khosravi, 2016; Noone, 2020; Shah, 2021; Thangavel, 2022). They have generally been described as technology‐based interventions to improve communication and social connection among older adults and there is no clear framework for their categorization (Fakoya, 2020). For example, they have been categorized as one‐on‐one or group‐based interventions (Cohen‐Mansfield, 2015; Dickens, 2011; Masi, 2011; Poscia, 2018) or based on four strategies or type (Masi, 2011) as:

- interventions for improving social skills (e.g., computer and internet training and use with a focus on reducing social isolation or loneliness, online university of the third age);
- interventions for enhancing social support that offer regular contacts, care, or companionship (e.g., telecare with a component to improve social connections, personal reminder information and social management systems (PRISMS), social robots or virtual pets, video games, 3D virtual environments or virtual spaces with trained coaches, conversational agents, or messaging capabilities);
- interventions for enhancing social interaction (videoconferencing, supported video communication, internet chat facilities, social networking sites, online discussion groups and forums, telephone befriending)
- interventions for social cognitive training (low intensity psychosocial interventions, internet‐delivered cognitive behavioral therapy (CBT), mindfulness interventions).

In mapping the body of available evidence, we categorized interventions by strategies to enable exclusive coding of interventions in categories and subcategories such that an intervention will fit into a single subcategory and not overlap with another on the evidence and gap map.

#### 2.1.3 Why it is important to develop the EGM

Several recent reviews of digital interventions for reducing social isolation and loneliness among older adults indicate there is growing research in this topic area most likely due to the ageing population (Boulton, 2020; Chen, 2016; Chipps, 2017; Findlay, 2003; Ibarra, 2020; Khosravi, 2016; Noone, 2020; Shah, 2021). In addition, the COVID‐19 pandemic restrictions have led to a dramatic expansion in the demand for digital technology interventions by people without access including older adults, for the provision of basic services like healthcare, education, and connections with other people (UCLG, 2020). Although there is a very wide range of such interventions, findings on their effectiveness, have sometimes been inconsistent (WHO, 2021). The body of evidence supporting their use is rapidly expanding, dispersed and uneven with lack of consistent terminology. Therefore, the best use of resources at this point for building the evidence architecture needed would be to develop an evidence and gap map on digital interventions to reduce social isolation and loneliness among older adults. This evidence and gap map will collate the evidence and display clusters of evidence and gaps in evidence that will serve as a resource to guide prioritization of further research and increase the accessibility and use of evidence for informed decision making by stakeholders including citizens, patients, caregivers, health and social care providers, policy makers and researchers.

#### 2.1.4 Existing EGMs and/or relevant systematic reviews

Recent reviews of digital interventions suggest that (a) there is a very wide range of such interventions; (b) findings on their effectiveness, although sometimes positive, are frequently mixed, inconclusive or uncertain; and (c) the technologies involved are developing rapidly (e.g., artificial intelligence, conversational agents, 3D virtual environments, video‐games, social networking tools) (Boulton, 2020; Chen, 2016; Chipps, 2017; Ibarra, 2020; Khosravi, 2016; Noone, 2020; Shah, 2021).

There is an evidence and gap map on specific remotely delivered interventions (i.e., befriending, social support, and low intensity psychosocial interventions) to reduce social isolation and loneliness among older adults (Boulton, 2020). It is based on a rapid review of reviews with systematic review evidence on befriending, social support, and low intensity psychosocial interventions that are delivered remotely to older adults, excluding caregivers. Study‐level evidence is limited to 18 individual studies in the 5 included systematic reviews.

Our evidence and gap map will be more comprehensive with a broader scope of all types of digitial interventions for older adults including older caregivers. It will examine up to date evidence from systematic reviews as well as primary studies and map available evidence to identify gaps and clusters in interventions and outcomes assessed.

## 3 OBJECTIVES

The aim is to map available evidence on the effects of digital interventions to mitigate social isolation and/or loneliness in older adults in all settings except hospital settings.

Specific objectives are as follows:

1. To identify existing evidence from primary studies and systematic reviews on the effects of digital interventions to reduce social isolation and/or loneliness in older adults.
2. To identify research evidence gaps for new high‐quality primary studies and systematic reviews.
3. To highlight evidence of health equity considerations from included primary studies and systematic reviews.

## 4 METHODS

We followed the Campbell Collaboration guidance for producing an evidence and gap map (White, 2020) described in the evidence and gap map protocol for this project (Welch, 2022).

### 4.1 Evidence and gap map: Definition and purpose

Evidence and gap maps are a systematic evidence synthesis product with a visual presentation of existing evidence relevant to a specific research question (Snilstveit, 2013; White, 2020). They display areas with collections or gaps in evidence and the quality of available evidence.

The evidence and gap map is typically a two dimensional matrix with interventions as row headings and outcomes as column headings (Snilstveit, 2016; White, 2020). Each cell within the matrix shows the studies with evidence on the corresponding intervention and outcome. This map identifies areas of evidence as well as any gaps in research related to using digital interventions for social isolation and/or loneliness among older adults.

### 4.2 Framework development and scope

We developed an intervention‐outcome framework for this evidence and gap map through a consultative process with stakeholders and adaptation of existing frameworks from systematic reviews, conceptual papers, and reports from stakeholder organizations.

A refined version of the WHO Classification of Digital Health Interventions framework (WHO, 2018) was initially considered at the Stakeholder consultation meeting on April 8, 2021. The WHO framework was developed to categorize the different ways in which digital and mobile technologies are used to support healthcare. The stakeholders found the typology of interventions to be too healthcare focused. The consensus was that a more user intuitive typology of interventions was needed to ensure the useability of this evidence and gap map for a larger audience including older adults. A needs‐based approach was preferred as interventions are most effective when they meet the needs and specific circumstances of the older adults (Abdi, 2019; Findlay, 2003; ten Bruggencate, 2019; WHO, 2020).

We identified other relevant frameworks from existing reviews and conceptual papers. We chose two frameworks which used a needs‐based approach (Jopling, 2020) and a strategy‐based approach (Masi, 2011) to address social isolation and loneliness and adapted them for our evidence and gap map.

**Figure 1:**
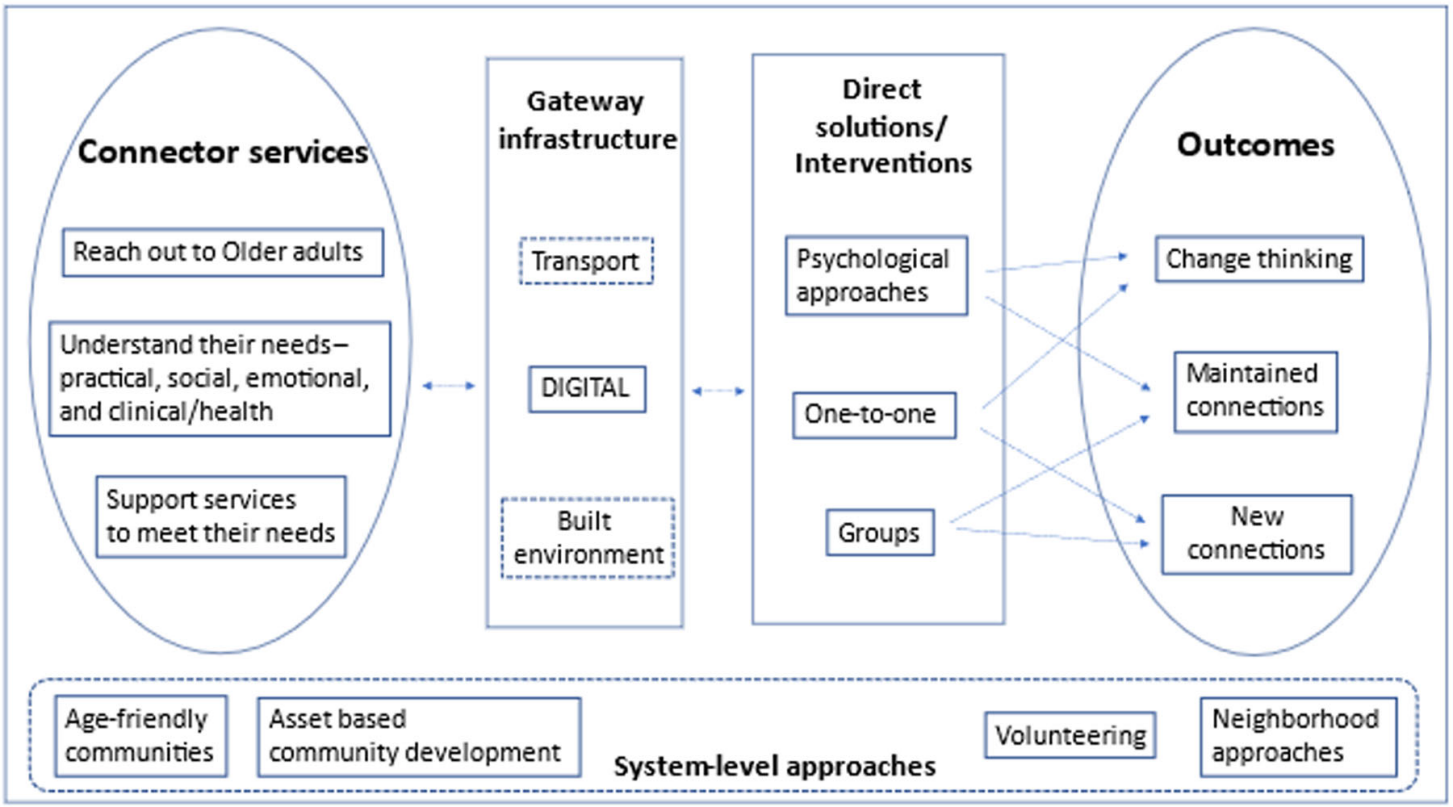
Needs‐based approach framework (Adapted from Jopling et al., 2020).

The needs‐based framework (Jopling, 2020) considers approaches to address loneliness and social isolation that are used in communities to achieve three outcomes: maintain and improve existing relationships or connections, support people to develop new connections, and to change negative thinking about their relationships. The approaches include connector services that reach out to understand the needs of older adults and provide support to meet the needs, gateway infrastructures through which people can connect with others, direct solutions or interventions to reduce loneliness and social isolation, and system‐level approaches that create environments in communities to facilitate tackling loneliness and social isolation (Figure 1).

The intervention categories in this framework do not provide mutually exclusive categorization of digital interventions. For example, many digital interventions such as computer and internet training, video chats, online CBT may be one‐to‐one, or group based. Hence the need for the second framework.

**Figure 2:**
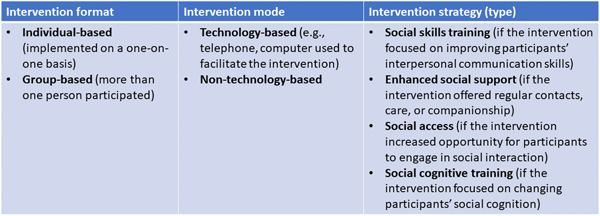
Strategy‐based approach framework (Adapted from Masi et al., 2011).

The strategy‐based model (Masi, 2011) describes strategies used in loneliness reduction interventions based on the understanding of the nature of loneliness and social isolation and how they affect people (Figure 2). Interventions were also categorized based on the format or level of delivery (as one‐on‐one or group interventions) or mode of delivery (technology‐based and non‐technology‐based interventions).

We used an intervention‐outcome framework where digital interventions of interest will be coded by the strategies to reduce loneliness and social isolation: strategies for (1) improving social skills, (2) enhancing social support, (3) enhancing social interaction, (4) social cognitive training, and (5) multicomponent strategies; as well as by the type of intervention (e.g., computer and internet training to reduce social isolation and loneliness, video chats, telephone befriending, telecare with a component to improve social connections, online CBT). See Supporting Information: Appendix [Link], [Link] for the glossary of key concepts.

Since the framework was bi‐dimensional (interventions and outcomes), the needs of socially isolated and lonely older adults were used as a filter on the map and coded interventions were mapped to the needs.

### 4.3 Outcomes

The impacts of interventions to prevent social isolation and loneliness have been measured at different levels – individual, community or societal, and process and implementation (Windle, 2012). In our framework, we considered outcomes that have been identified as indicators of social connection and they were categorized based on the impact and level of influence of the interventions:

- individual outcomes – loneliness, social isolation, social connectedness, quality of life, anxiety/depression, confidence level, information, communication and technologies (ICT) knowledge and experience, adverse effects;
- community outcomes – social support, social engagement, social cohesion, social capital, digital divide (disparities in access to technological interventions);
- process indicators – acceptance, adherence, technology use, feasibility, cost‐effectiveness, affordability, barriers.

### 4.4 Stakeholder engagement

We convened an advisory board of 30 stakeholders from organizations such as the International Red Cross, Canadian Red Cross, Agewell, Canadian Frailty Network, HelpAge, CanAge, Centre for Ageing Better, United Nation Department of Social and Economic Affairs, United Nations Fund for Population Activities (UNFPA), and the WHO. The group of stakeholders included representatives of these key organizations, policymakers, and academics with an interest in mitigating social isolation and loneliness in older adults. The advisory board provided comments on the intervention‐outcome framework. The WHO Classification of Digital Health Interventions framework was considered. Stakeholders suggested a simplified framework to fit the purpose of this evidence and gap map. The framework was revised based on their feedback, and stakeholders were consulted by email for their feedback on the revised framework included in this review.

We consulted with four citizens in two citizen focus groups between June and August 2021. Some iterations were suggested, that is, coding for interventions related to the need of finding purpose in later life, and capturing interventions related to recreation and physical activity. Affordability and access to technology were recommended for consideration as outcomes in the framework.

We created an anonymous survey with four questions and invited stakeholders including citizens by email to respond to the survey questions and provide their feedback on the revised framework and draft map.

### 4.5 Conceptual framework

**Figure 3:**
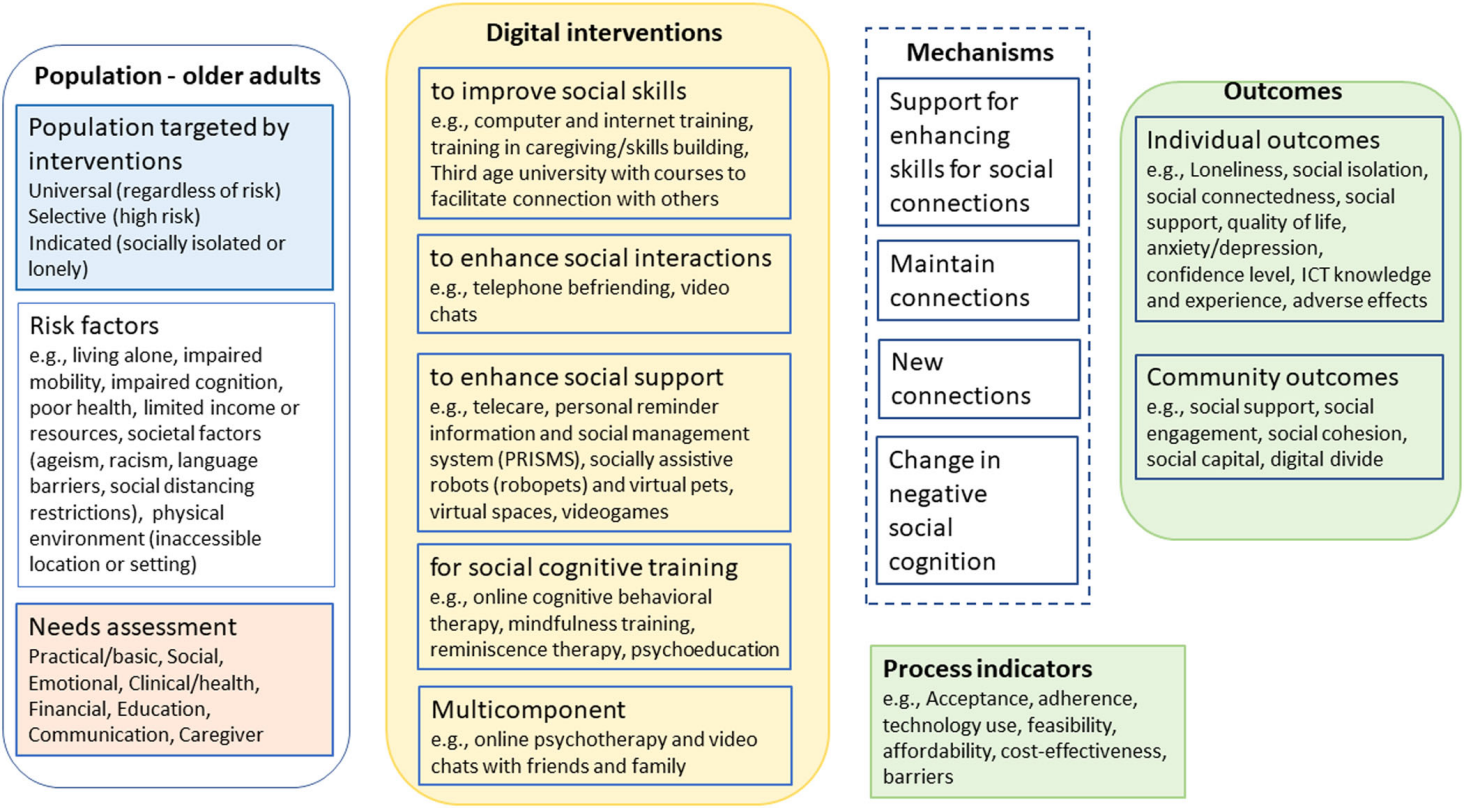
Conceptual framework.

Our conceptual framework (Figure 3) was based on the understanding of the needs of older adults, how social isolation and loneliness can occur and how they affect older adults' well‐being. The relationship between these variables can be explained by a potential pathway of effect illustrated in the conceptual framework going from risk factors and needs assessment for older adults to interventions, the mechanisms of change, and process indicators and outcomes.

#### 4.5.1 Population targeted by interventions

Older adults are more susceptible to experiencing social isolation and loneliness, but risk exposure may vary with individual contexts. Older adults at risk of isolation and loneliness have been identified by their age, gender, place of residence or other factors (Elder, 2012; Fakoya, 2020; NASEM, 2020). Interventions may target all older adults regardless of their risk (universal) or a subpopulation of older adults who are at risk, for example, those living in nursing homes (selective) or may target those who are socially isolated or lonely (indicated).

#### 4.5.2 Risk factors for social isolation and loneliness

Social isolation and loneliness have been associated with low social support and a disruption in social interactions established with other people at any level (individual, group, community, and societal or system) which can lead to unmet needs (Abdi, 2019; Donovan, 2020; Tomaka, 2006). Major changes in life such as change or loss of social network, social participation or role, physical health, mental health and financial resources can also lead to social isolation and loneliness (Donovan, 2020; Newall, 2019; Victor, 2005). Other risk factors for social isolation and loneliness include living alone, societal factors (racism, language barriers, ageism, social distancing and restrictions) and the physical environment (inaccessible location or community setting) (Berkman, 2000; DeGood, 2011; Donovan, 2020).

#### 4.5.3 Needs assessment for older adults

Ageing is associated with a decline in physical and cognitive health, difficulty with mobility, activities of daily living and household routines which put older adults at risk of experiencing needs that require health and social support. These needs include social and emotional needs (social connections and companionship), civic engagement (meaningfulness and status, the need for having a purpose in later life or being able to contribute usefully to society), healthcare, housing, home modifications and maintenance, domestic assistance, mobility, nutrition and food security, personal care, education (skills development and learning), financial management, respite care, caregiver support, communication (language support or interpreters, information and assistance/referral services) (Abdi, 2019; Bedney, 2010; Henderson, 2008; Jopling, 2020; WHO, 2020).

Support services have been developed to satisfy the needs of older adults and to promote wellbeing and healthy ageing through social networks or relations (Abdi, 2019; Jopling, 2020; ten Bruggencate, 2019; WHO, 2020).

Social isolation and loneliness may be caused by multiple factors and people respond differently depending on their age and coping skills. It is therefore important to reach out to the older adults to understand their circumstances (the risk factors they are facing and their needs) to be able to provide tailored support for social connections or for accessing services as approaches to reduce social isolation and loneliness (Jopling, 2020; ten Bruggencate, 2019).

#### 4.5.4 Digital interventions

Different approaches have been used to reduce social isolation and loneliness including facilitating social connections and providing social support. By providing social support services to meet their needs, opportunities for social connections could be created which could reduce social isolation and loneliness in older adults. Support for social connections and companionship or for accessing services can be provided through digital technology.

Based on the understanding of the nature and impact of social isolation and loneliness, different strategies have been used in digital interventions to mitigate social isolation and loneliness (Masi, 2011). We used these strategies as the typology of intervention categories. Since multiple factors may be involved, multicomponent strategies may also be used to address social isolation and loneliness. The categories include:

1. interventions to improve social skills,
2. interventions to enhance social interactions,
3. interventions to enhance social support,
4. interventions for social cognitive training, and
5. multicomponent interventions.

### 4.6 Mechanisms

The impact of digital interventions can be achieved by four mechanisms of change:

1. providing support to building skills for social connections (e.g., computer and internet training and use, online university of the third age),
2. maintaining existing connections (e.g., video chat with family and friends, PRISMS to engage family and friends in helping receive care),
3. creating new connections (e.g., telephone befriending programs, social networking sites, robots and virtual pets, videogames), and
4. by changing negative social cognition (e.g., online CBT to teach lonely people to identify and free themselves from negative thoughts and feelings about their relations such as a perception of lack of intimate attachment to their friends or family).

These mechanisms do not map onto the four strategies since some interventions may reduce social isolation or loneliness through more than one mechanism. For example, social networking sites may be used to reduce social isolation and loneliness by maintaining existing connections and by creating new connections. Computer and internet training can be used to maintain connection with family and friends or to create new connections.

### 4.7 Process indicators and outcomes

The effects of interventions depend on how well the interventions were implemented. Process indicators measure activities or outputs that indicate whether the intervention was implemented as planned (Milstein, 1999). They are preconditions that contribute to the outcomes and are therefore considered proximal indicators of implementation processes or intermediate outcomes (Proctor, 2011).

Different levels of outcome measurements for the effects of digital interventions include:

- process indicators – acceptance, adherence, technology use, feasibility, costs, barriers.
- individual level outcomes – loneliness, social isolation, social connectedness, quality of life, anxiety/depression, confidence level, information, communication and technologies (ICT) knowledge and experience, adverse effects.
- community level outcomes – social support, social engagement, social cohesion, social capital, digital divide (disparities in access to technological interventions).

We used this conceptual framework to define and code the dimensions (categories and subcategories of interventions and outcomes as well as filters) for the evidence and gap map.

### 4.8 Dimensions

#### 4.8.1 Types of study design

Eligible study designs to be included weree completed or on‐going systematic reviews, and primary studies with any form of control group including randomized controlled trials (RCTs), and evaluative quasi‐experimental designs with a control group.

We included systematic and scoping reviews based on their PICO question if they explicitly described adequate search methods used to identify studies, eligibility criteria for selection of included studies, methods of critical appraisal of included studies and synthesis or analysis of included studies (Moher, 2015).

Quasi‐experimental design studies were considered eligible if the assignment of participants was based on allocation rules such as alternate assignment (quasi‐randomized studies), inclusion of a threshold on a continuous variable (regression discontinuity designs), exogenous variation in the treatment allocation (natural experiments) or other rules including self‐selection by investigators or participants, provided data were collected contemporaneously in a comparison group (non‐equivalent comparison group design), or an interrupted series design with at least three data points both before and after a discrete intervention (six‐period interrupted time series) (Waddington, 2014).

We excluded all studies that used less than six period interrupted time series design, or primary studies without a comparison group design like longitudinal cohort studies with no controls, and cross‐sectional studies. We also excluded literature reviews that were not sysematic reviews. However, systematic reviews which also include studies without a comparison group design will be included.

We did not include qualitative research.

#### 4.8.2 Types of intervention/problem

We defined digital interventions as technology‐based interventions to improve communication and social connection. We considered all types of digital interventions with the aim to reduce social isolation and loneliness. These digital interventions were either one‐to‐one, or group based. They may focus on loneliness, social isolation, or both. We considered any frequency or duration of administration.

We included the following types of digital interventions categorized by strategies.

- Interventions to improve social skills: these are interventions that focus on training in interpersonal social skills such as conversational skills with the aim to enable individuals to form and maintain meaningful relationships. Examples are computer and internet training and use to communicate with others, or online university of the third age which includes courses to facilitate communication with others. We excluded studies that assess computer and internet training for digital literacy and do not assess the use of internet to reduce social isolation or loneliness by focusing on improving social skills.
- Interventions to enhance social support: these are interventions that offer support (e.g., regular contacts, care, or companionship) and guidance in finding and attending new activities or groups. They aim to help individuals make and maintain social connections. Examples include telecare with a component to improve social connections, PRISMS, online support groups and forums, social robots or virtual pets, video games, 3D virtual environments. We excluded studies that assess interventions for care without a communication component or a component to improve connecting with other people, for example, smart home technologies like sensors for monitoring falls, e‐health for clinical need only, online CBT for dementia care only, online referral systems for healthcare coordination.
- Interventions to enhance social interactions: these are interventions that focus on improving the quality of relationships and increase opportunities for social interactions. They aim to promote connections with family/friends or community and include internet chat facilities, social networking sites, online discussion groups and forums or telephone befriending. Although telephone befrienders could also provide social support, we classify telephone befriending as an intervention to enhance social interactions since the main aim for the service is to connect regularly and build friendship with an older person (Boulton, 2020; Gardiner, 2018).
- Interventions for social cognitive training: these are interventions that focus on changing negative thinking and feelings about social relationships. They aim to change behaviors, reduce maladaptive cognitions, and increase social connections. Examples include low intensity psychosocial interventions, internet‐delivered CBT, or mindfulness interventions.

See Table 1 for categories and other examples.

**Table 1:** Intervention categories.

Strategy‐based categories and subcategories	Examples
Interventions to improve social skills	Training in how to use digital technology for communication – for example, Computer and Internet training and use Digitally delivered training (e.g., about caregiving/skills building) Digitally delivered learning – for example, learning a new language, Third age university with courses to facilitate connection with others
Skills development Learning a new activity/language or learning about social skills	
Interventions to enhance social interaction	Social connections with family/friends – for example, video chats Social connections with community – for example, telephone befriending with volunteers from community
Maintain connections New connections	
Interventions to enhance social support	Digital/remote ehealth services – for example, telecare with a component to improve social connections (HomMed Health Telemonitoring system with a communication component) Digital social and health care coordination with family/friends – for example, Personal reminder information and social management system (PRISMS) with a communication component Geolocating/identifying older adults who need services (e.g., Age UK loneliness heat maps) Socially assistive robots (robopets) and virtual pets Virtual spaces Virtual assistants (e.g., Google home, Alexa) Virtual social support groups Digital intergenerational approaches Digital games (e.g., scrabble, chess, cards, exergames) Digitally delivered activities(e.g., exercise – tai chi, yoga,) to mitigate social isolation and loneliness Digital coordination of health or social care services (e.g., online referrals with a component to improve social connections)
Healthcare support Social care support	
Social cognitive training interventions	Digital cognitive behavioral therapy Digital mindfulness training Digital psychoeducation Digital reminiscence therapy Digital cognitive behavioral coaching
Multicomponent interventions	Including any of the above in a mixed format (e.g., computer training, messaging, and chat groups)

Comparators were no interventions, other interventions, or usual care.

#### 4.8.3 Types of population

We included older adults, defined as 60 years of age or older (WHO, 2020). If studies included younger and older adults, we included the studies if data could be disaggregated. If data could not be disaggregated, we included studies if the mean age of all participants was at least 65 years of age. To be inclusive, studies or reviews which stated a focus on older adults without providing the age of participants were included.

#### 4.8.4 Types of outcome measures

Outcomes included loneliness, social isolation, as well as other indicators of social connections. Potential harms such as ethical concerns, privacy violations, liability and cyber‐attacks as well as unintended consequences such as increase in social isolation and loneliness, were also included. Community outcomes such as social support, social engagement, social cohesion, social capital, and digital divide (disparities in access to technological interventions) as well as process indicators (acceptability, adherence, technology use, feasibility, affordability, cost‐effectiveness, and barriers), especially for vulnerable populations, were included (See Table 2).

**Table 2:** Outcome categories.

Outcomes	Acceptable measurements
*Individual outcomes*	
Loneliness	UCLA loneliness scale, de Jong‐Gierveld loneliness scale, other scales, for example, Social and Emotional Loneliness Scale, Hughes loneliness scale
Social isolation	Lubben's Social Network Scale, Social Network Index, PROMIS social isolation 6‐I scale
Social connectedness/interactions/networks or life satisfaction	Lee and Robin's Social Connectedness Scale; Number of contacts; Frequency of social interactions; Satisfaction with interaction; Index of support satisfaction; Support network satisfaction; Companionship scale satisfaction
Social support	Duke‐UNC Functional Social Support Questionnaire; Social support scale by Schuster et al.; Hsiung's Social Support Behaviors Scale; Family and Friendship Contacts Scale; Personal Resource Questionnaire; Interpersonal Support Evaluation List (ISEL); e‐Diabetes Social Support Scale; a bespoke six‐item scale measuring women's perception of emotional and instrumental support
Well‐being/Quality of life	MOS SF‐36 Health Survey; Work and Social Adjustment Scale (WSAS);
Anxiety/depression	Beck Depression Inventory (BDI); Depression Adjective Check List (DACL) Form E; Geriatric depression scale; The Centre for Epidemiological Studies Depression Scale (CES‐D)
Confidence level	Rosenberg Self‐Esteem Scale
Information, communication and technology (ICT) knowledge and experience	Questionnaire
Adverse effects	Privacy violations, liability, cyber‐attacks, negative effect on well‐being from emotional attachment to devices
*Community outcomes*	
Social support	Duke‐UNC Functional Social Support Questionnaire, Social support scale, social Provisions scale
Social engagement	Engagement in Meaningful Activities Survey (EMAS)
Social cohesion	The Group Cohesion Scale‐Revised; Group Therapy Experience Scale, Group Environment Questionnaire
Social capital	The World Bank's integrated questionnaire for the measurement of social capital (SC‐IQ)
Digital divide	Lack of affordability/access to technology, lack of affordability/access to broadband or Wi‐Fi, data poverty, lack of digital skills or confidence to access services and support online
*Process indicators*	
Acceptability (technology adoption)	Various survey tools to measure acceptability
Adherence (training adherence)	Various survey tools to measure adherence
Technology use	Frequency of use
Feasibility	Various survey tools to measure feasibility
Affordability	Various survey tools to measure affordability
Cost‐effectiveness	Cost‐effectiveness analysis
Barriers	For example, language and cultural barriers, financial accessibility, hearing or vision impairments, personal barriers such as dislike of robopets, digital literacy, lack of familiarity with digital technologies, Lack of confidence in using digital technologies

Outcomes were not used as eligibility criteria. However, eligible studies and systematic reviews did have a focus on social isolation and loneliness. Studies and reviews assessing interventions with a stated aim to reduce social isolation and loneliness were eligible. Those that assessed the effects of interventions on social isolation and/or loneliness as a primary outcome or considered other indicators of social connections including quality of life, anxiety/depression, social support, social engagement, social cohesion, and social capital were also included.

Studies and reviews assessing the effect of interventions on anxiety or depression with a focus on mental health rather than social isolation or loneliness were excluded.

#### 4.8.5 Other eligibility criteria

##### Types of location/situation

We included all country settings as defined by the WHO regions (African Region, Region of the Americas, South‐East Asian Region, European Region, Eastern Mediterranean Region, Western Pacific Region) (WHO, 2019) and the World Bank classification by income: low‐income economies, lower‐middle income economies, upper‐middle income economies, high‐income economies (World Bank, 2021).

Primary studies and systematic reviews that did not report the countries were not excluded.

##### Types of settings

All settings except hospital settings were included, that is, people living in supportive care institutions (nursing home or long‐term care and assisted living facilities) and in the community (residential or personal home).

### 4.9 Search methods and sources

We designed a search strategy with an information scientist (DS) in consultation with Tomas Allen (WHO information specialist). We searched the following databases from inception to May 16, 2021 with no language restrictions: Ovid MEDLINE, Embase, APA PsycInfo via Ovid, CINAHL via EBSCO, Web of Science via Clarivate, ProQuest (all databases), International Bibliography of the Social Sciences (IBSS) via ProQuest, EBSCO (all databases except CINAHL), Global Index Medicus, and Epistemonikos. The full search strategies are in Supporting Information: Appendix [Link], [Link].

We screened reference lists of all included systematic reviews in Eppi‐Reviewer to identify additional studies. We also contacted stakeholders for information about ongoing studies.

### 4.10 Analysis and presentation

#### 4.10.1 Report structure

The report has the standard sections: abstract, plain language summary, background, methods, results, discussion, and conclusion.

The report includes the flow of studies, included studies, excluded studies and any studies awaiting assessment, as well as synthesis of included studies. We presented the PRISMA flowchart and conceptual framework. We also included tables and figures that provide a summary of the distribution of primary studies and systematic reviews across the coding categories such as the type of studies, quality of the systematic reviews, types of interventions, needs, types of populations, outcomes, settings, and geographic distribution.

**Figure 4:**
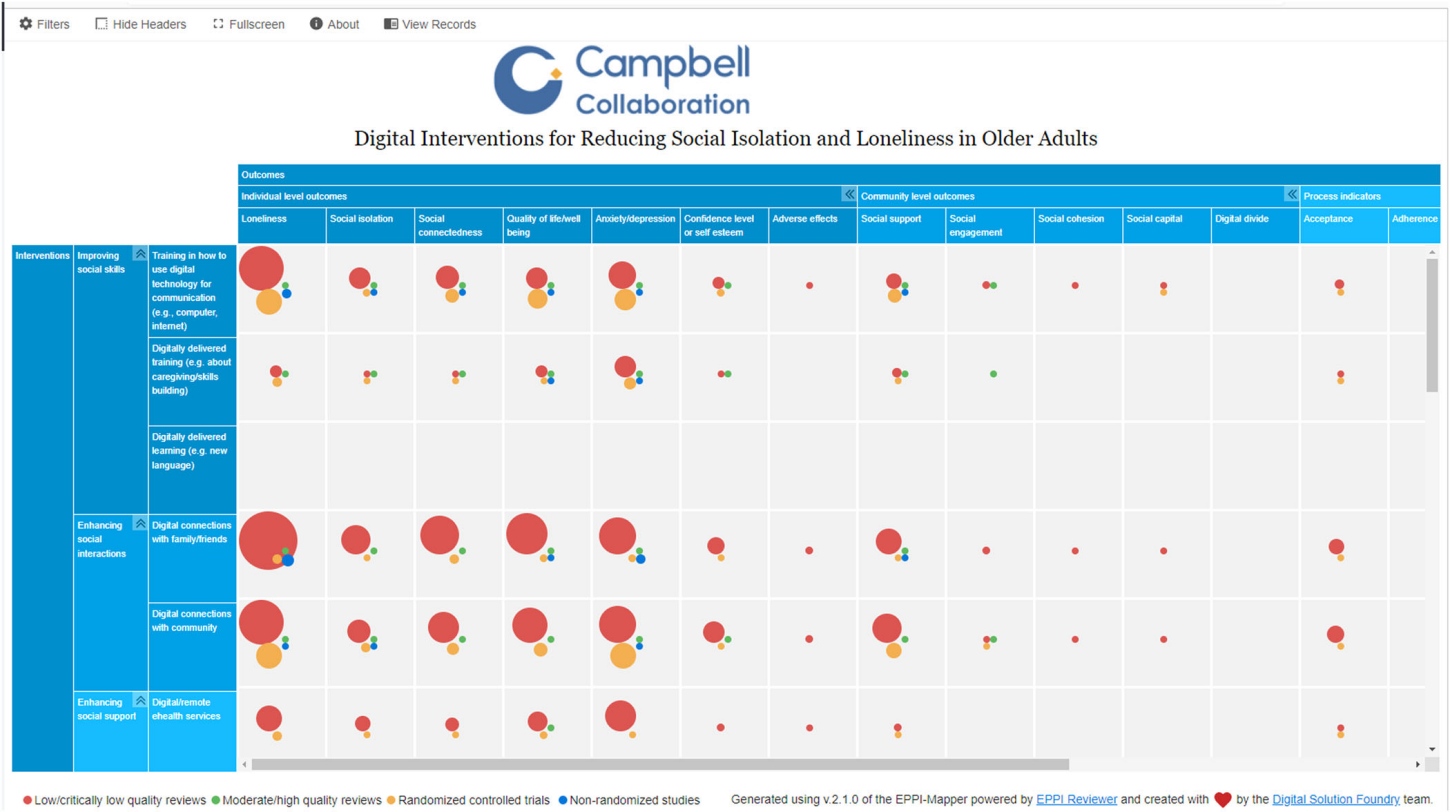
Sample map.

The evidence and gap map has interventions as the row dimension and outcomes as the column dimension. Bubbles of different sizes represent included studies and different colors are used to identify the primary studies and methodological quality of the systematic reviews. The filters used in the map depend on the number of included studies and coded information. See a sample of the map in Figure 4.

#### 4.10.2 Filters for presentation

Additional dimensions of interest that were used as filters included the publication status of included studies, study design, World Bank classification by income (low‐income economies, lower‐middle income economies, upper‐middle income economies, high income economies), and WHO regions (African Region, Region of the Americas, South‐East Asian Region, European Region, Eastern Mediterranean Region, Western Pacific Region), setting (personal home, independent living/residential home, assisted living, long‐term care/nursing home), health status/condition.

We documented which needs beyond mitigating social isolation and/or loneliness of older adults are being met by digital interventions, using a framework developed from our citizen and stakeholder engagement consultation, which includes social and emotional needs, civic engagement and social participation, healthcare, housing, home modifications and maintenance, domestic assistance, mobility, nutrition and food security, personal care, education, financial management, respite care, caregiver support, communication.

We documented the focus of the intervention as aimed at social isolation, loneliness, or both. We also had filters for the intervention format, how technologies are used, as well as how training on how to use digital technology was delivered.

##### Equity analysis

We documented whether studies were focused on populations who are at risk or experiencing barriers to health and social care or health inequities across age, gender or sex, ethnicity, income, or other factors. We used the PROGRESS‐Plus acronym to describe factors associated with health inequities (O'Neill, 2014). For these studies, we documented how potentially vulnerable older adults are defined and identified (e.g., using case finding, outreach, screening).

In addition, for each study, we assessed whether studies analyzed differences in effects for populations experiencing inequities, using the PROGRESS factors (Place of residence (urban/rural), Race/ethnicity/culture and language, Occupation, Gender or sex, Religion, Education, Socioeconomic status, Social capital. We also assessed analysis across additional “Plus” factors which are known to be important for older adults, including age, disability, social frailty, health status, being socially isolated or at risk, being lonely or at risk, living situation, and digital literacy.

#### 4.10.3 Dependency

Multiple reports of the same study were treated as one study. A study with multiple interventions or outcomes was shown multiple times on the map (for each intervention or outcome identified). Systematic reviews were mapped to the interventions and outcomes as defined by the question of the systematic review. Primary studies that met the eligibility criteria were mapped as well regardless of whether they are included in one or more systematic reviews.

### 4.11 Data collection and analysis

#### 4.11.1 Screening and study selection

Titles and abstracts and full text of potentially eligible articles were screened independently following the eligibility criteria in duplicate (by VW, EG, VB, PG, TH, SA, NE, JE, HW and OD) using the Eppi‐Reviewer web‐based software program (Thomas, 2020). We screened systematic reviews based on their PICO questions. Disagreements were resolved by discussion. See Supporting Information: Appendix [Link], [Link] for detailed eligibility criteria.

We used machine learning text mining to support screening at the title and abstract stage. After screening approximately 10% of the titles and abstracts, we used the priority screening function which developed a classifier based on the probability of inclusion determined from the preliminary screening results. We, however, double screened all the search results to ensure all potentially eligible studies were captured for the full text screening stage.

We also screened reference lists of eligible systematic reviews to identify additional studies.

#### 4.11.2 Data extraction and management

We developed and pilot tested a data extraction code set in Eppi‐Reviewer for data collection of the dimensions for the map (see extraction code in Supporting Information: Appendix [Link], [Link]). After the pilot test, members of the team (EG, VB, PG, TH, EB, AW, AA, and SD) individually extracted and coded data. Automation and text mining were not used for coding.

The coding categories included study characteristics (study design, publication status, methodological quality assessment of systematic reviews), intervention categories and subcategories, intervention focus (loneliness, social isolation, or both), intervention format, how technologies are used, how training on how to use digital technology was delivered, outcome domains and subdomains, population characteristics, needs, setting, and location (countries, WHO regions and World Bank classification by income) (Supporting Information: Appendix [Link], [Link]).

We coded the location (country) if it was reported for the primary study or the included studies for reviews. If a review had multiple studies conducted in the same country, it received only one code for the country. For example, a review with six included studies conducted in the USA and two studies conducted in Australia, had only two codes – one for USA and one for Australia.

We coded description of the population characteristics using the PROGRESS‐Plus framework, defined as Place of residence (urban/rural), Race/ethnicity/culture and language, Occupation, Gender/sex, Religion, Education, Socioeconomic status, Social capital (marital status) and additional (plus) factors such as age groups, health status/condition, frailty, disability, living situations, digital literacy, social isolation, and loneliness.

We considered how the study population was selected based on whether they are disadvantaged across any PROGRESS‐Plus factors.

We also coded whether there was any analysis that aimed to understand potential differences across any PROGRESS‐Plus factors.

Given the expected size of the map (over 200 studies), we did not contact organizations or authors of studies and systematic reviews for missing information.

#### 4.11.3 Tools for assessing risk of bias/study quality of included reviews

We assessed the methodological quality of systematic reviews in duplicate (by EG, SD, VB, TH, NE and AW) using the AMSTAR 2 tool (Shea, 2017). Any disagreements were resolved by discussion. As per guidance for evidence maps, primary studies were not assessed for risk of bias or methodological quality (Snilstveit, 2016; White, 2020). A modified AMSTAR2 assessment was conducted for 20 scoping reviews; we did not assess items about risk of bias of included studies since it is optional for scoping reviews (Peters, 2021).

#### 4.11.4 Methods for mapping

We used the EPPI‐Mapping tool (Digital Solution Foundry and EPPI_Centre, 2020) to develop the evidence and gap map.

## 5 RESULTS

### 5.1 Description of studies

#### 5.1.1 Results of the search

**Figure 5:**
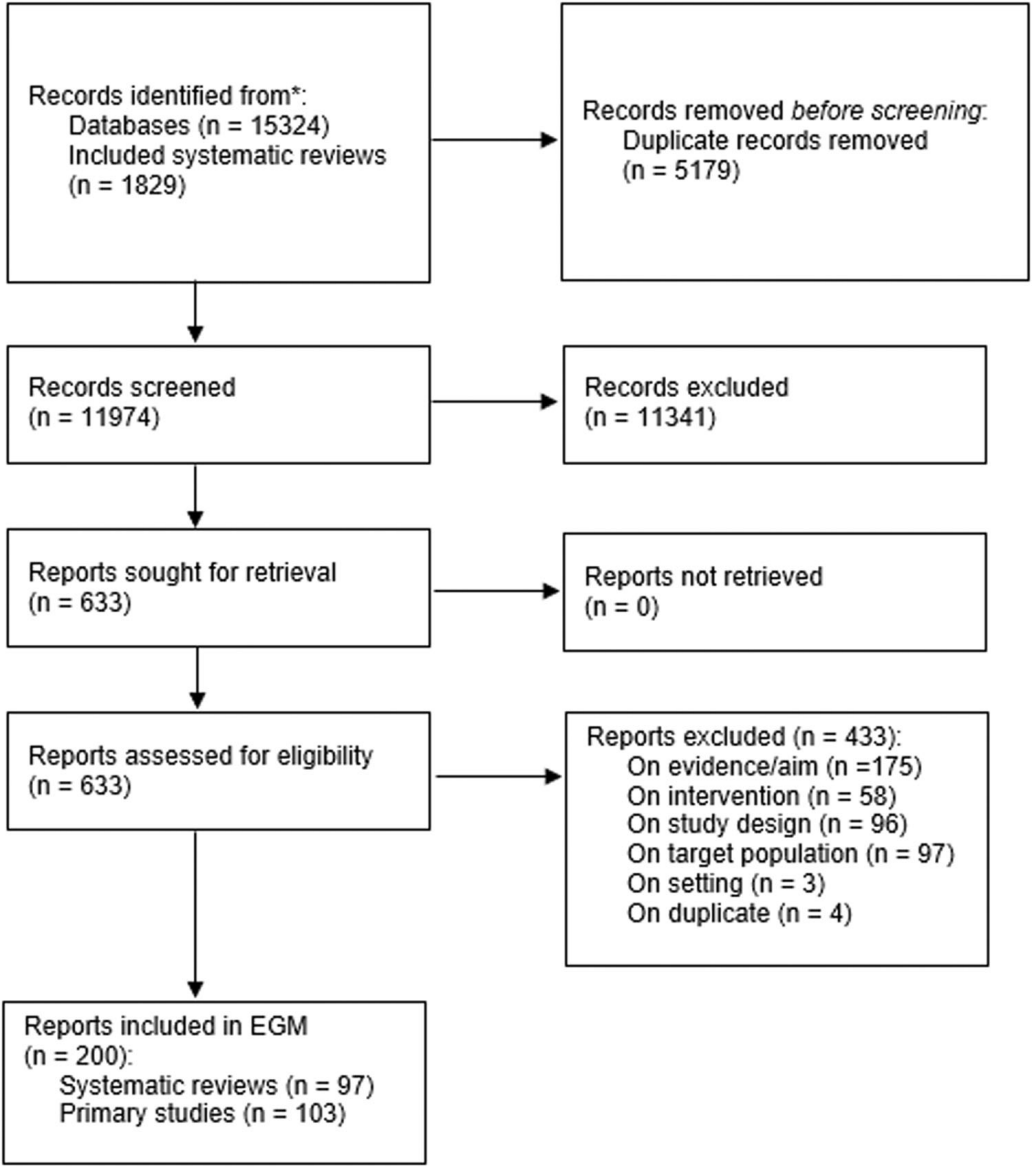
PRISMA flow diagram.

Our search retrieved 15,324 records from databases and 1829 records from included systematic reviews. After duplicate records were removed, 11,974 articles were screened by title and abstract in duplicate. From this, 633 articles were assessed for eligibility, in duplicate. Two hundred articles were included for the purpose of this EGM. See Figure 5 for the PRISMA flow diagram.

#### 5.1.2 Excluded studies

A total of 433 studies were excluded at the full‐text review phase due to inappropriate evidence/aim (*n* = 175), inappropriate intervention (*n* = 58), inappropriate study design (*n* = 96), inappropriate target population (*n* = 97), inappropriate setting (*n* = 3), or duplicate (*n* = 4). See Figure 5 for the PRISMA flow diagram.

See key excluded studies in the table for Characteristics of excluded studies. Three studies (Bolle, 2015; Clarkson, 2018; Sumner, 2021) were excluded because the aim was not to reduce social isolation or loneliness. Bolle (2015) assessed online health information tools to improve health outcomes. Clarkson (2018) assessed home support interventions to inform dementia care and Sumner (2021) assessed co‐designed interventions to support ageing in place.

Six studies did not assess digital interventions (Dickens, 2011; Gine‐Garriga, 2017; Jones, 2019; Nicholson, 2012; Toh, 2016; Zeppegno, 2018).

Eight studies were excluded for wrong study design; five (Fan, 2016; Forsman, 2018; Gorenko, 2021; Selak, 2019; Winterton, 2011) were not systematic reviews of effectiveness and three were primary studies with no control groups (Perkins, 2012; Preston, 2019; Rebollar, 2015).

Six studies (Burkow, 2015; Cooper, 2014; Dam, 2017; Erfani, 2018; Lara, 2016; Nijman, 2019) included people less than 60 years old and did not provide disaggregated data for people 60 years and older.

### 5.2 Synthesis of included studies

We included 200 articles that utilized digital interventions to reduce social isolation and/or loneliness; 103 were primary studies and 97 were systematic reviews. See interactive EGM  [https://onlinelibrary.wiley.com/pb-assets/assets/18911803/Campbell%20map-Oct5_WITH-1697552065.html].

The primary studies included both RCTs (*n* = 81) and non‐randomized studies (NRSIs) (*n* = 22). The systematic reviews included both systematic reviews (*n* = 77) and scoping reviews (*n* = 20) that explored the effectiveness of interventions to reduce social isolation and loneliness. Most of the included publications were completed (*n* = 170, 78 primary studies and 92 reviews) while others were ongoing registered trials and protocols (*n* = 25, 21 primary studies and 4 reviews). There were five conference abstracts included (*n* = 5, 4 primary studies and 1 review). See Table 3 (Characteristics of included articles).

**Table 3:** Characteristics of included articles.

Characteristics of included articles		Number of articles
Study design	Primary studies – randomized studies – non‐randomized studies	**103** 81 22
	Reviews – systematic reviews – scoping reviews	**97** 77 20
Publication status	Completed On‐going Conference abstracts	78 primary studies/92 reviews 21 primary studies/4 reviews 4 primary studies/1 review
Intervention focus	Social isolation Loneliness Both social isolation and loneliness	34 primary studies/54 reviews 41 primary studies/23 reviews 34 primary studies/54 reviews
Intervention format	One‐on‐on Group‐based One‐on‐one and group based Unspecified	29 primary studies/10 reviews 56 primary studies/23 reviews 11 primary studies/28 reviews 7 primary studies/26 reviews
Intervention strategies to reduce social isolation and loneliness	Social cognitive training Improving social skills Enhancing social interactions Enhancing social support Multicomponent	21 primary studies/19 reviews 37 primary studies/40 reviews 30 primary studies/58 reviews 49 primary studies/81 reviews 16 primary studies/3 reviews
Outcomes	Individual level outcomes Community level outcomes Process indicators	95 primary studies/96 reviews 40 primary studies/40 reviews 28 primary studies/31 reviews
Most reported population sociodemographic characteristics	Gender/sex Age range (70–80 years old) Education Health condition (dementia)	83 primary studies/78 reviews 78 primary studies/55 reviews 50 primary studies/6 reviews 16 primary studies/30 reviews
Settings	Personal home Long‐term care (nursing homes) Independent living (residential homes) Assisted living	36 primary studies/45 reviews 22 primary studies/45 reviews 12 primary studies/23 reviews 5 primary studies/10 reviews
Most common locations for included studies	USA The Netherlands UK Canada Australia	42 primary studies/44 reviews 8 primary studies/25 reviews 6 primary studies/22 reviews 6 primary studies/16 reviews 5 primary studies/22 reviews

#### 5.2.1 Interventions

The intervention focus for included publications was on addressing social isolation and loneliness together (*n* = 88, 34 primary studies and 54 reviews), or on loneliness (*n* = 64, 41 primary studies and 23 reviews) or social isolation (*n* = 48, 28 primary studies and 20 reviews) alone.

Included publications reported delivery of interventions as one‐on‐one (*n* = 79, 56 primary studies and 23 reviews), group‐based (*n* = 39, 29 primary studies and 10 reviews), or both one‐on‐one and group‐based (*n* = 49, 11 primary studies and 38 reviews). The mode of delivery was unspecified in some publications (*n* = 33, 7 primary studies and 26 reviews).

**Figure 6:**
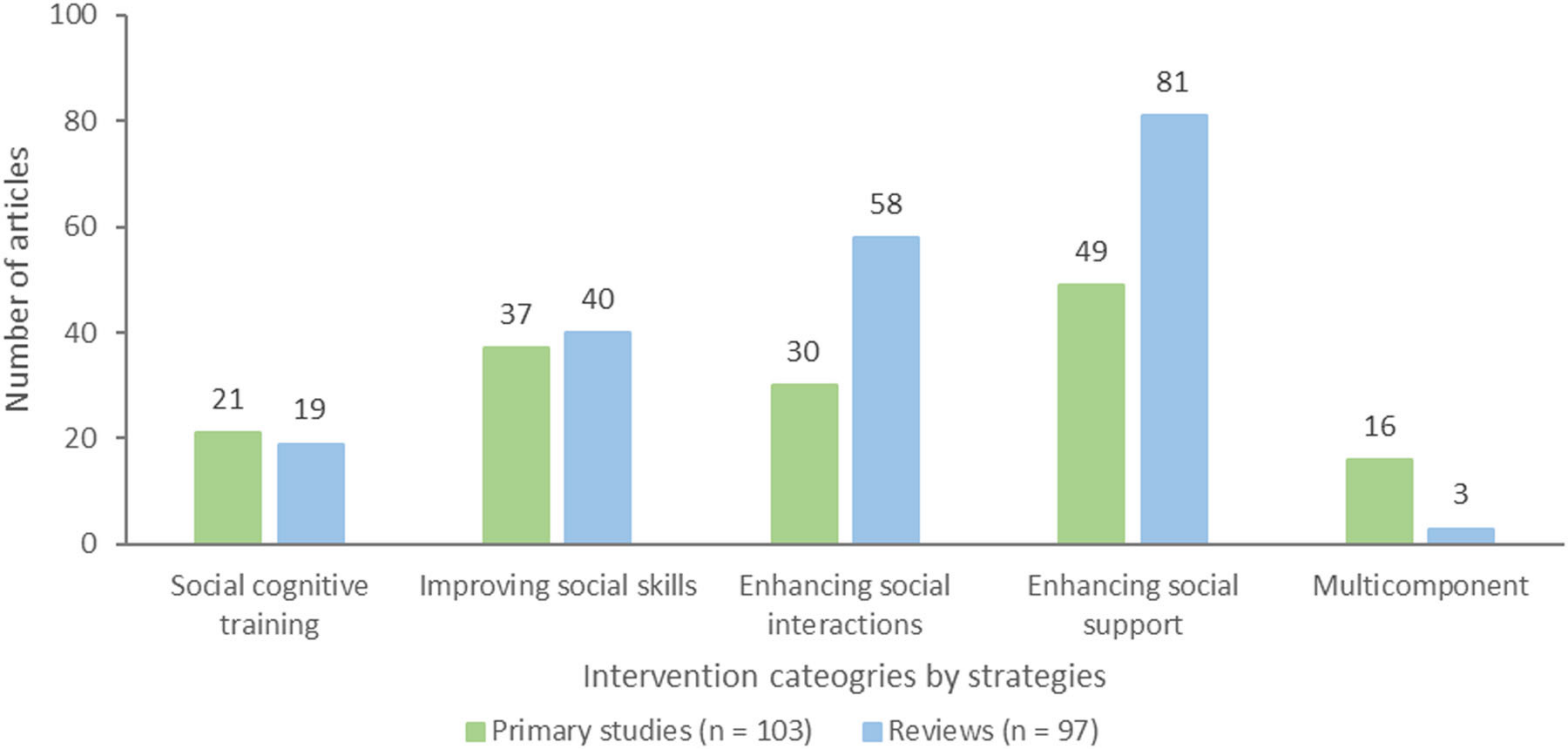
Intervention strategies to reduce social isolation and loneliness.

Interventions were categorized based on their strategies to reduce social isolation or loneliness: interventions for social cognitive training (*n* = 40, 21 primary studies and 19 reviews), improving social skills (*n* = 77, 37 primary studies and 40 reviews), enhancing social interactions (*n* = 88, 30 primary studies and 58 reviews), enhancing social support (*n* = 130, 49 primary studies and 81 reviews), and multicomponent interventions (*n* = 19, 16 primary studies and 3 reviews) (Figure 6).

**Figure 7:**
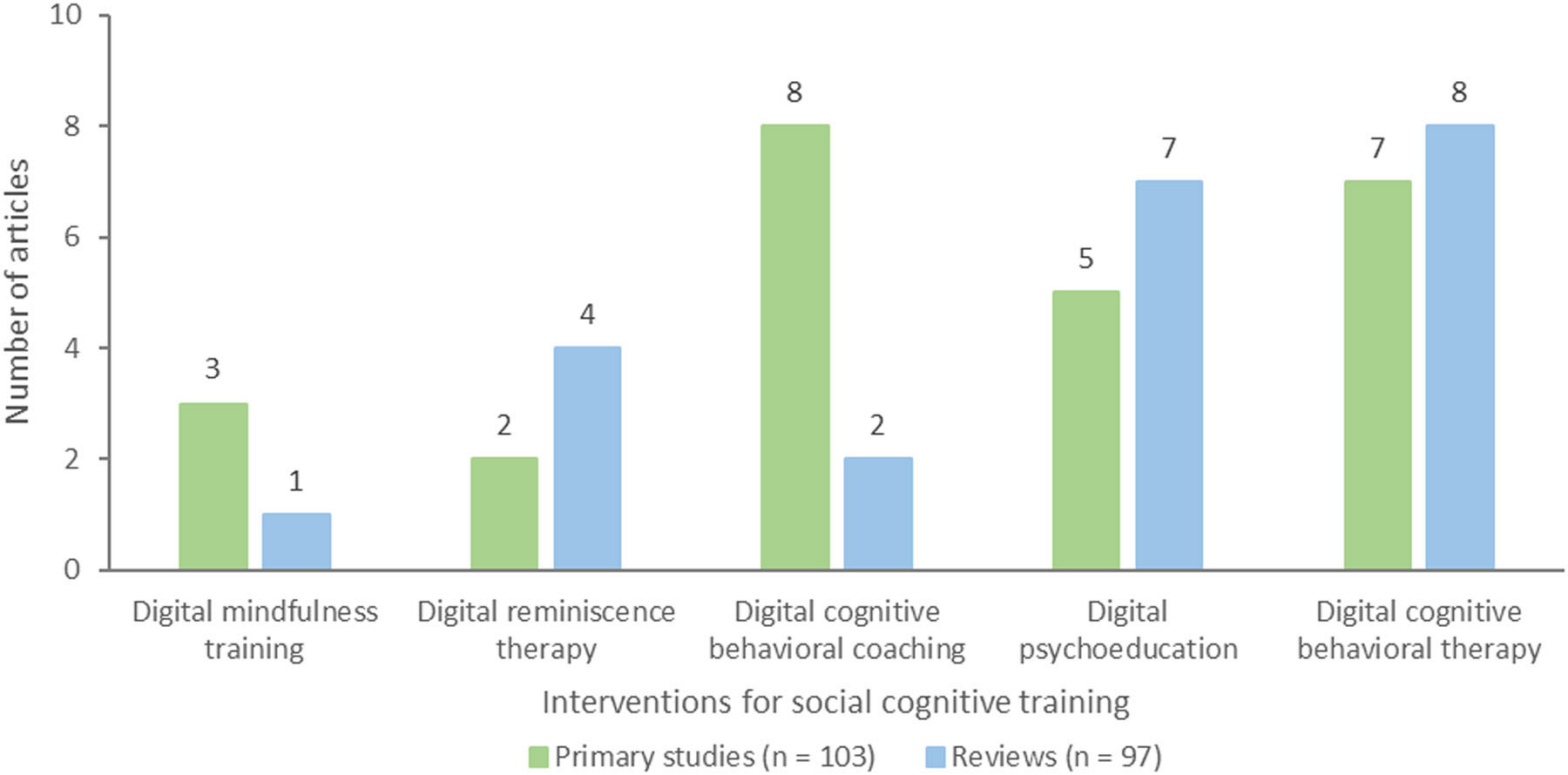
Interventions for social cognitive training.

Each coded intervention category included multiple subcategories. Interventions for social cognitive training consisted of five subcategories: digital CBT (*n* = 15, 7 primary studies and 8 reviews), digital psychoeducation (*n* = 12, 5 primary studies and 7 reviews), digital cognitive behavioral coaching (*n* = 10, 8 primary studies and 2 reviews), digital reminiscence therapy (*n* = 6, 2 primary studies and 4 reviews), and digital mindfulness training (*n* = 4, 3 primary studies and 1 review) (Figure 7).

CBT approaches help individuals recognize and change negative thinking whereas cognitive behavioral coaching approaches help individuals reach their desired goals. Reminiscence therapy involves recalling past events and encourages older adults to communicate and interact with someone in the present. Mindfulness training teaches people to consciously pay attention to their thoughts and feelings and to disengage from negative thoughts, and unhealthy habits that may render them vulnerable. Psychoeducation therapy develops knowledge and understanding of psychological conditions to help individuals cope with them.

**Figure 8:**
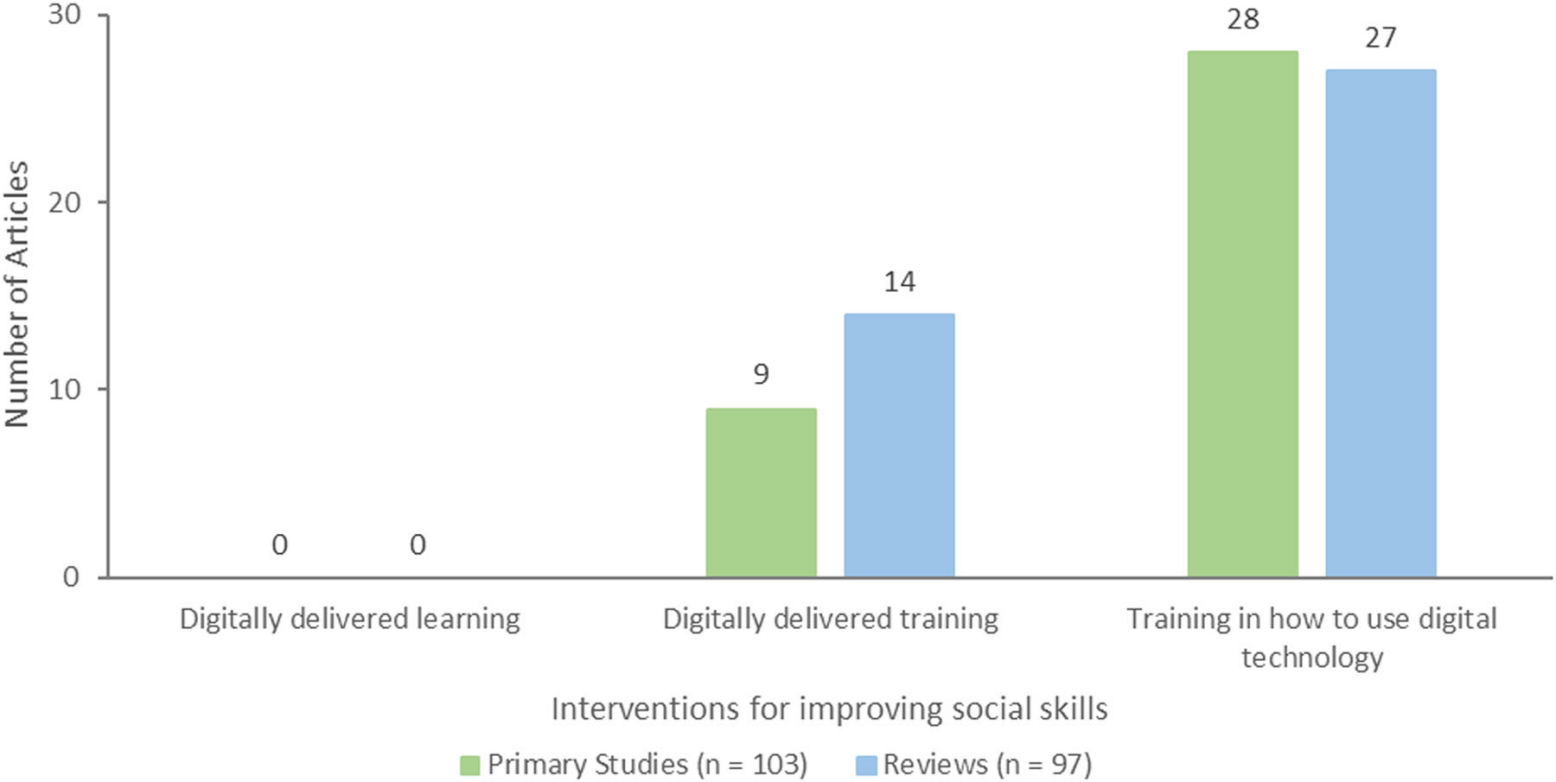
Interventions for improving social skills.

Interventions for improving social skills had three subcategories: training in how to use digital technology for communication was most frequent (*n* = 55, 28 primary studies and 27 reviews), followed by digitally delivered training (*n* = 23, 9 primary studies and 14 reviews), and no studies or reviews for digitally delivered learning (Figure 8). Training in how to use digital technology was mainly about computer and internet training for communication with family and friends. Half of the primary studies and reviews on digitally delivered training interventions were technology‐based interventions for caregivers of older adults with dementia.

**Figure 9:**
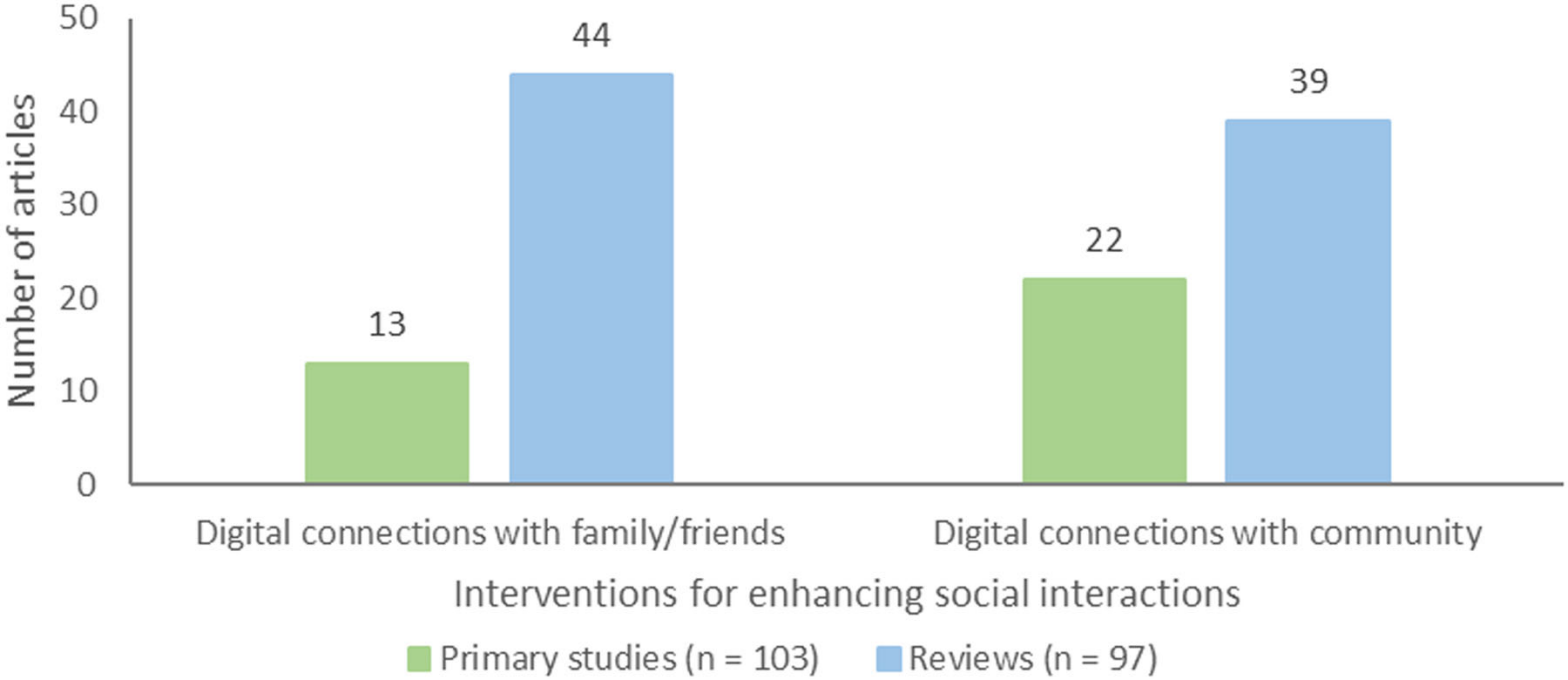
Interventions for enhancing social interactions.

The interventions for enhancing social interactions comprised two subcategories: digital connections with family/friends (*n* = 57, 13 primary studies and 44 reviews), and digital connections with community including healthcare and social workers and volunteers (*n* = 61, 22 primary studies and 39 reviews) (Figure 9).

Interventions for enhancing social support had 11 subcategories with most studies involving the following 6 subcategories: socially assistive robots and virtual pets (*n* = 51, 10 primary studies and 41 reviews), digital games (*n* = 29, 9 primary studies and 20 reviews, virtual spaces (*n* = 28, 11 primary studies and 16 reviews), digital/remote e‐health services that also provided companionship or social connections (*n* = 28, 6 primary studies and 22 reviews), virtual social support groups (*n* = 27, 10 primary studies and 17 reviews) and digitally delivered activities, mostly physical exercise and exergaming activities (*n* = 19, 9 primary studies and 20 reviews). Few studies and reviews assessed digital social and healthcare coordination with family/friends (*n* = 5, 1 primary study and 4 reviews), geolocating/identifying older adults who need services (*n* = 2 reviews), virtual assistants (*n* = 3, 1 primary study and 2 reviews), digital intergenerational approaches (*n* = 3 reviews), and digital coordination of health or social care services (*n* = 4, 2 primary studies and 2 reviews) (Table 4).

**Table 4:** Interventions for enhancing social support.

Interventions for enhancing social support	Primary studies (*n* = 103)	Reviews (*n* = 97)
Geolocating/identifying older adults who need services	0	2
Digital intergenerational approaches	0	3
Virtual assistants	1	2
Digital social and health care coordination with family/friends	1	4
Digital coordination of health or social care services	2	2
Digital/Remote e‐health services	6	22
Digitally delivered activities	9	10
Digital games	9	20
Virtual social support groups	10	17
Socially assistive robots and virtual pets	10	41
Virtual spaces	12	16

#### 5.2.2 Outcomes

**Figure 10:**
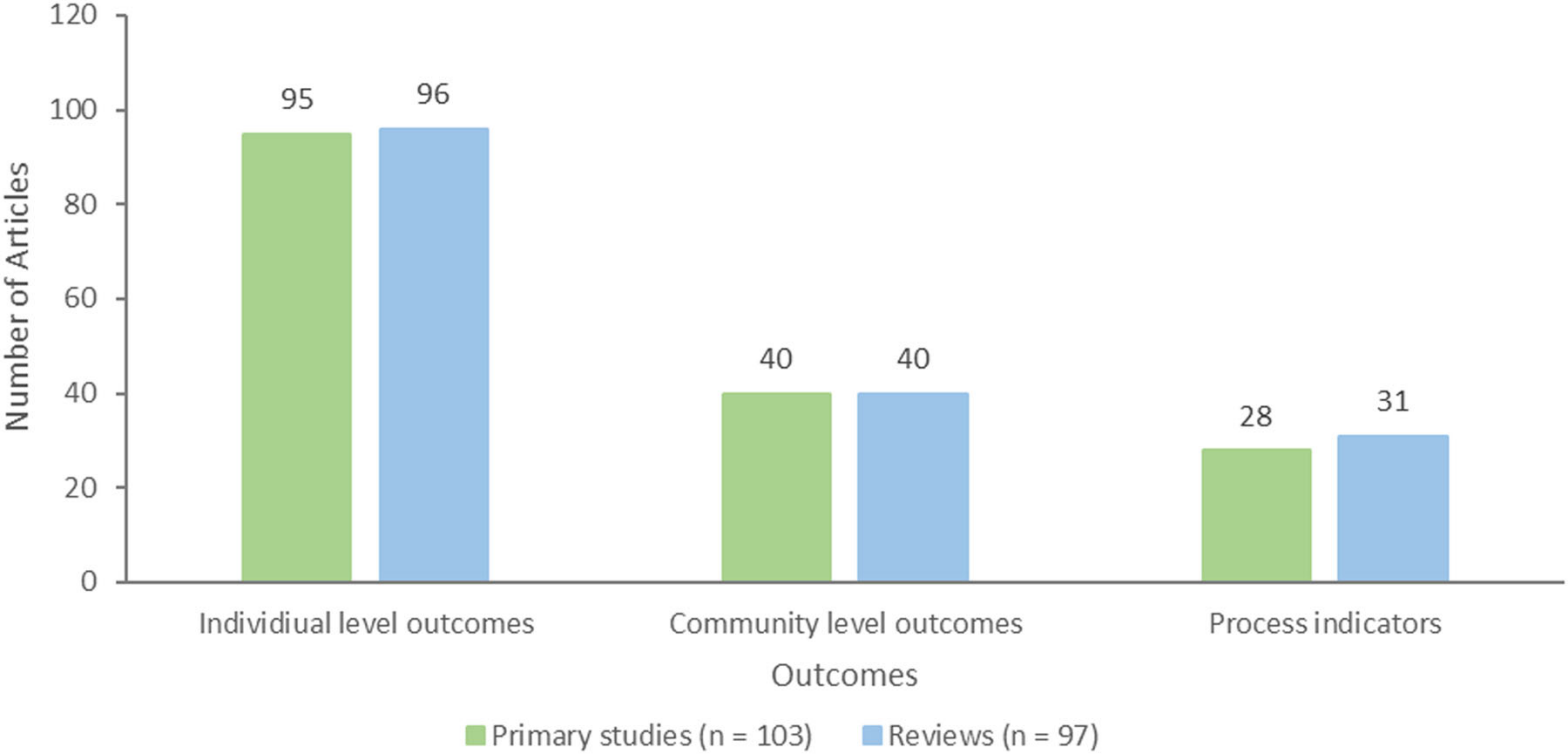
Outcome categories.

Three outcome categories were used to code publications: individual level outcomes (*n* = 191, 95 primary studies and 96 reviews), community level outcomes (*n* = 80, 40 primary studies and 40 reviews), and process indicators (*n* = 59, 28 primary studies and 31 reviews) (Figure 10).

**Figure 11:**
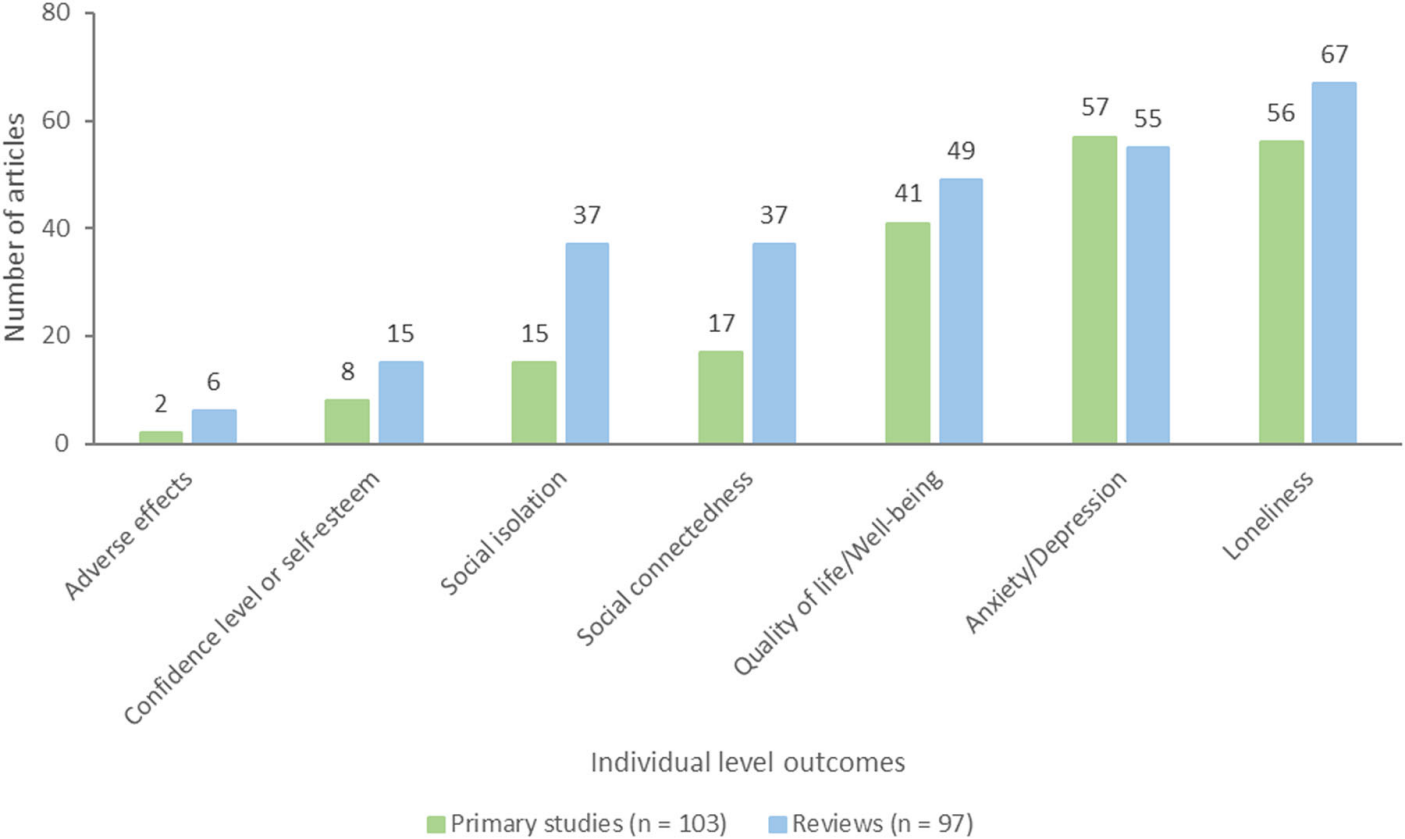
Individual level outcomes.

Many publications reported multiple outcomes and all outcomes of interest were coded. Individual level outcomes included: loneliness (*n* = 123, 56 primary studies and 67 reviews), anxiety/depression (*n* = 112, 57 primary studies and 55 reviews), quality of life/wellbeing (*n* = 90, 41 primary studies and 49 reviews), social connectedness (*n* = 54, 17 primary studies and 37 reviews), social isolation (*n* = 52, 15 primary studies and 37 reviews), confidence level or self‐esteem (*n* = 23, 8 primary studies and 15 reviews), and adverse effects (*n* = 8, 2 primary studies and 6 reviews) (Figure 11).

Adverse effects were mostly privacy and ethical issues reported in three reviews, excessive attachment to robopets with detrimental effects in one review, aggravated musculoskeletal symptoms and fall risks from exergaming in one review, negative impact of adverse media in social networking sites reported in one review, and increase in loneliness in one review. The two primary studies were on‐going and planned to measure adverse effects but did not indicate which ones were anticipated.

**Figure 12:**
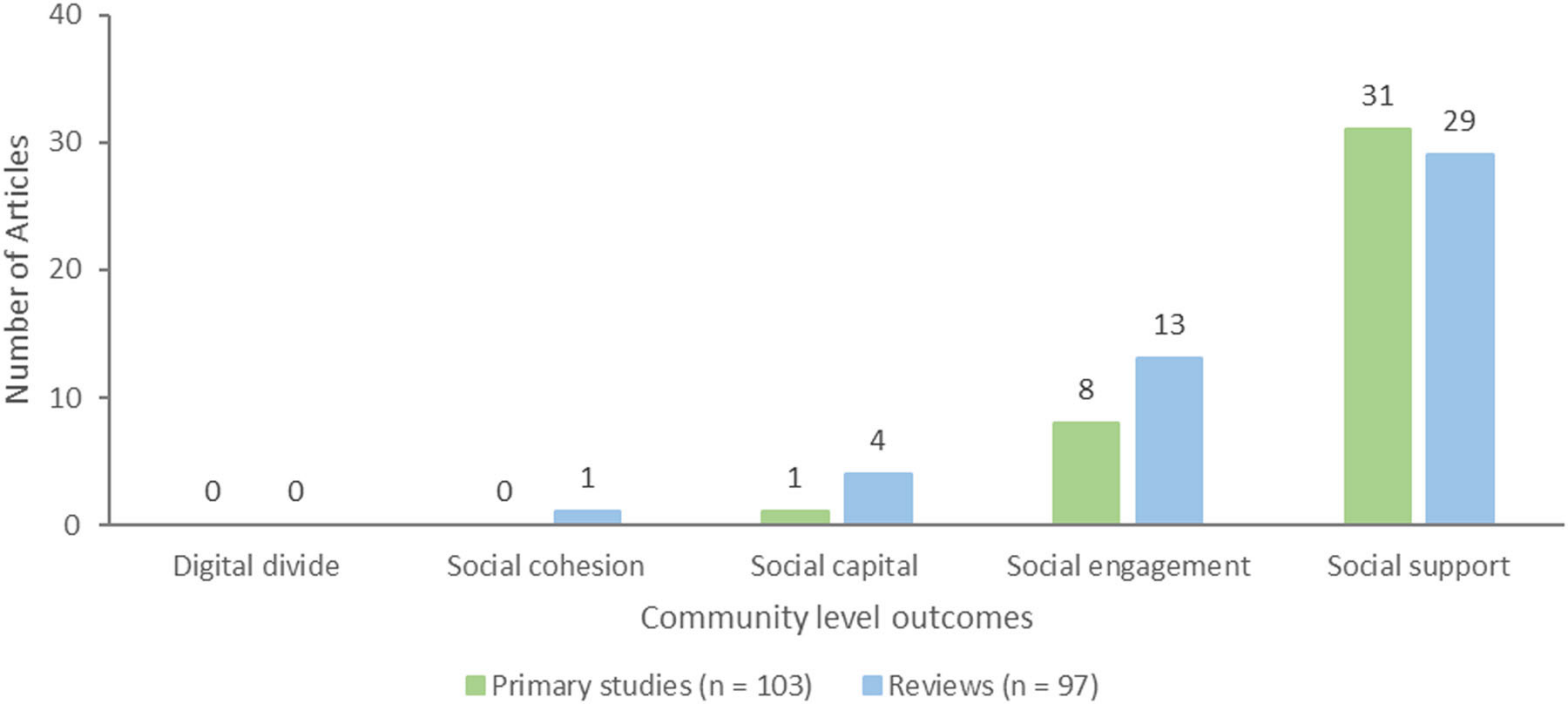
Community level outcomes.

Community level outcomes consisted of the following: social support defined as the actual or perceived availability of resources (e.g., tangible, informational, emotional help) from others, typically one's social network (*n* = 60, 31 primary studies and 29 reviews), social engagement which reflects participation in meaningful activities with others (*n* = 21, 8 primary studies and 13 reviews), social capital which refers to an appraisal of the social resources or networks people can access in their community (*n* = 5, 1 primary study and 4 reviews), social cohesion, a measure of mutual community trust and solidarity (*n* = 1 review). Digital divide, defined as disparities in access to technological interventions (e.g., smartphones, computers, and the internet) which may be due to lack of affordability or access to technology, broadband or Wi‐Fi, data poverty or geographic location, lack of digital skills and the confidence to access online services and support (*n* = 0) was not measured in any of the included articles (Figure 12). However, one review discussed the barriers caused by digital divide and three primary studies and three reviews each discussed the value of accessibility of digital interventions.

**Figure 13:**
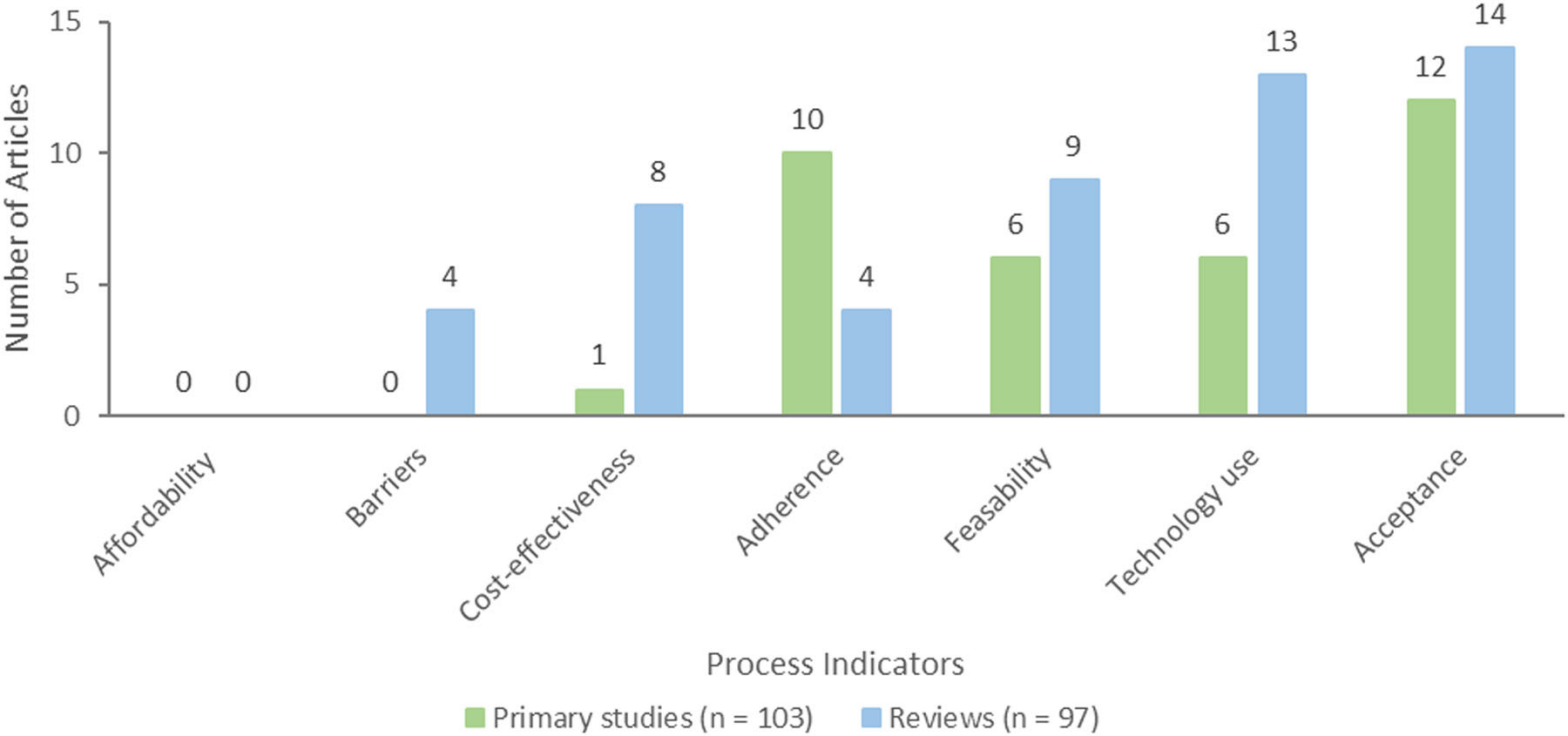
Process indicators.

Lastly, process indicators consisted of seven outcomes: acceptance (*n* = 26, 11 primary studies and 14 reviews), technology use (*n* = 19, 6 primary studies and 13 reviews), feasibility (*n* = 15, 6 primary studies and 9 reviews), adherence (*n* = 14, 10 primary studies and 4 reviews), cost‐effectiveness (*n* = 9, 1 primary study and 8 reviews), barriers (*n* = 4 reviews), and affordability (*n* = 0) which was not measured in any of the included studies or reviews (Figure 13).

### 5.3 Risk of bias in included reviews

**Figure 14:**
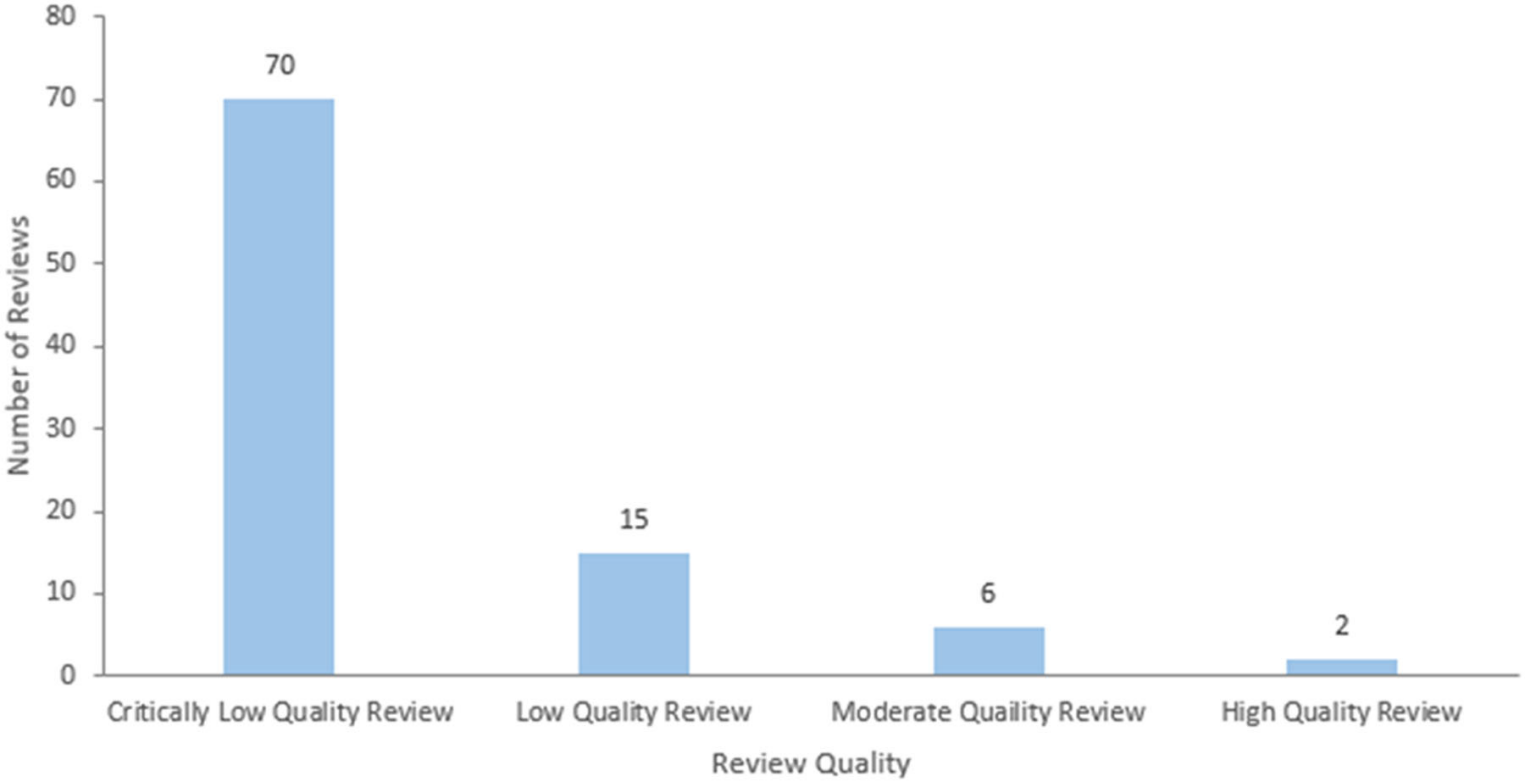
Quality assessment of reviews.

Most reviews were classified as critically low quality on the AMSTAR2 Quality Assessment tool due to critical flaws including not reporting prior establishment of a protocol; not providing a list of all excluded studies and justifying the exclusion of each study; as well as not assessing the risk of bias of included studies. The 97 included systematic reviews were classified as follows: critically low (*n* = 70), low (*n* = 15), moderate (*n* = 6), and high (*n* = 2) quality. Protocols (*n* = 4) were not assessed (Figure 14).

### 5.4 Additional dimensions

#### 5.4.1 How technologies are used

Technology was used in various ways, and multiple methods were considered in some publications. The most utilized method was videoconferencing (*n* = 65, 23 primary studies and 42 reviews), followed by telephone calls (*n* = 55, 20 primary studies and 35 reviews) and robots or virtual companions (*n* = 51, 10 primary studies and 41 reviews). Mobility tools (*n* = 1 review), listening to or creating music (*n* = 3 reviews) and virtual assistants (*n* = 4, 1 primary study and 3 reviews) were among the least ways in which technology was utilized, and 10 publications (5 primary studies and 5 reviews) did not specify how technologies were used (Table 5).

**Table 5:** How technologies are used.

How technologies are used	Primary studies (*n* = 103)	Reviews (*n* = 97)
Mobility tools	0	1
Listening to or creating Music	0	3
Virtual assistants	1	3
Discussion forums	3	11
Participating in an activity	15	6
Social networking sites	12	16
Digital games	10	20
Messaging	14	17
E‐mail	13	22
Virtual spaces or classrooms with messaging capabilities	16	28
Robots and virtual companions	10	41
Telephone calls^a^	20	35
Videoconferencing	23	42
Unspecified	5	5

^a^ Smartphones were specified in 32 of 55 primary studies and systematic reviews. For this EGM, we considered all telephone calls as a strategy for remotely delivered interventions.

#### 5.4.2 Region

Publications included in this EGM come from different countries worldwide. We coded the countries where primary studies were conducted. For reviews, if multiple included studies were conducted in the same country, we coded the country just once. However, if primary studies were included in multiple reviews, the countries where they were conducted were coded multiple times (i.e., once for each review).

**Figure 15:**
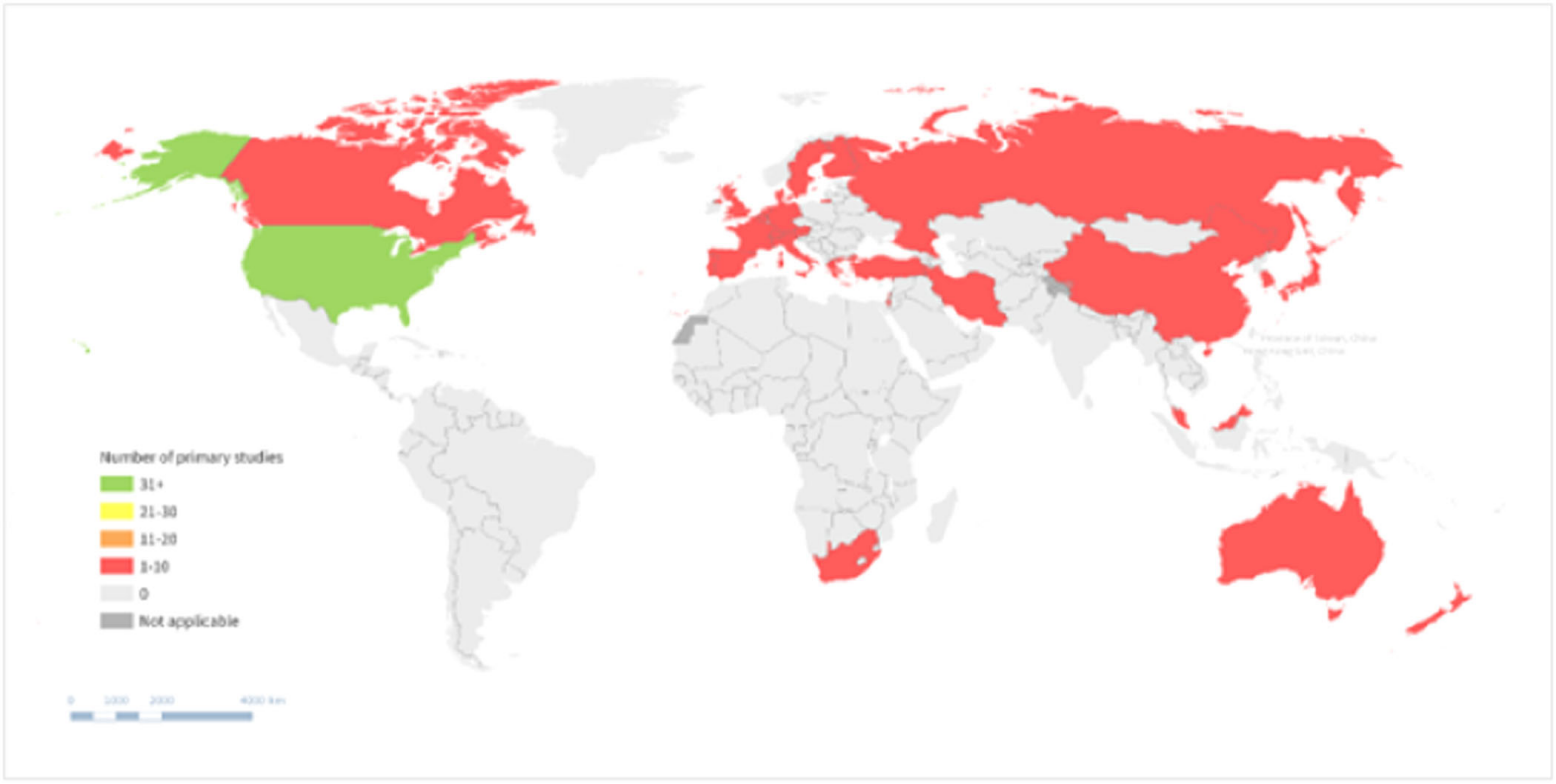
Geographic heat map for primary studies.

Many of the included publications had at least one study conducted in the USA (*n* = 86, 42 primary studies and 44 reviews) followed by the Netherlands (*n* = 33, 8 primary studies and 25 reviews), UK (*n* = 28, 6 primary studies and 22 reviews), Canada (*n* = 22, 6 primary studies and 16 reviews) and Australia (*n* = 27, 5 primary studies and 22 reviews). (See geographic heat map for primary studies in Figure 15).

**Figure 16:**
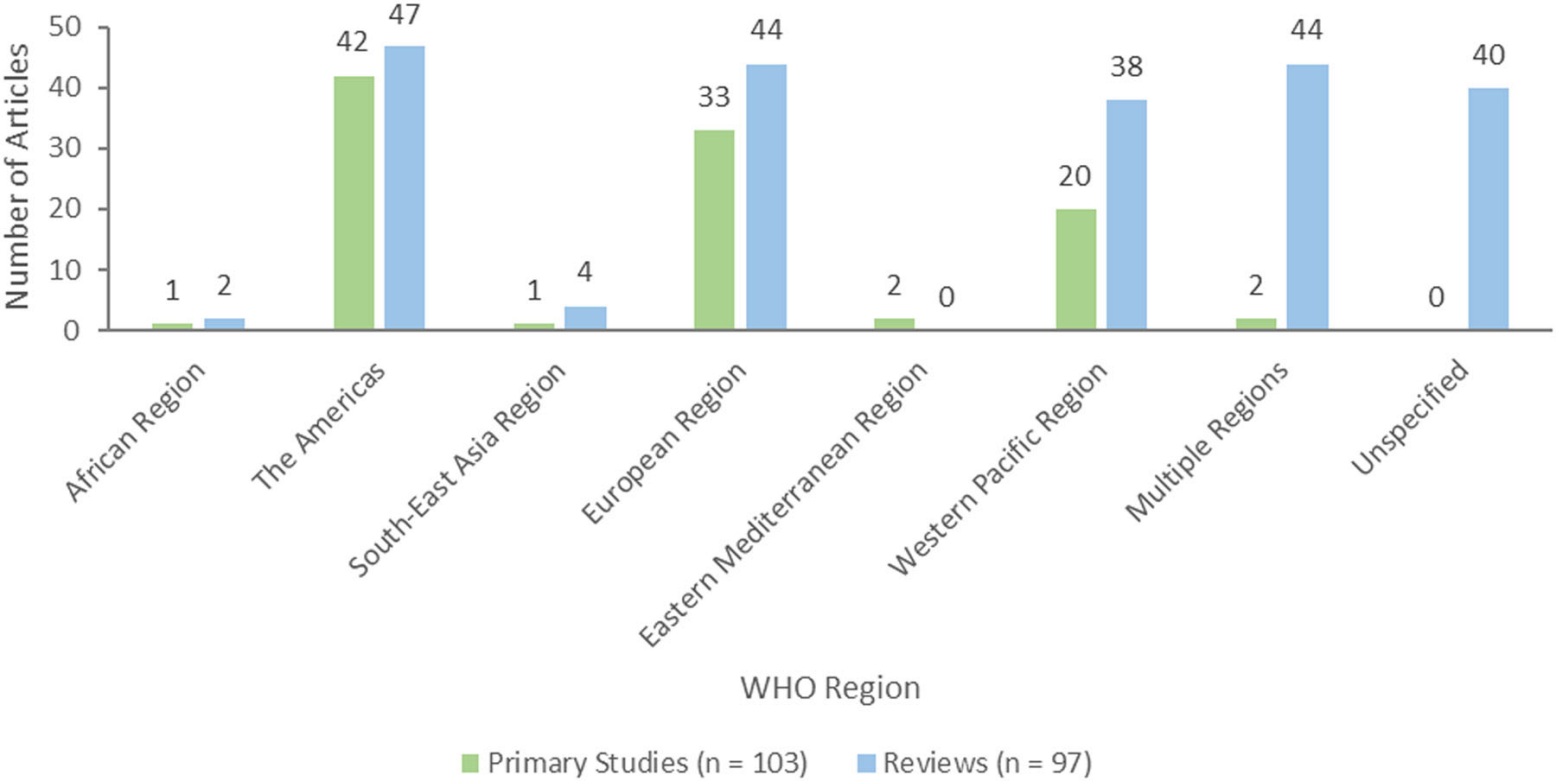
World Health Organization regions.

In terms of classification by WHO regions (Figure 16) most publications were from the Americas (*n* = 95, 48 primary studies and 47 reviews), the European Region (*n* = 77, 33 primary studies and 44 reviews) or Western Pacific Region (*n* = 58, 20 primary studies and 38 reviews), while very few were from the South‐East Asian (*n* = 5, 1 primary study and 4 reviews), African (*n* = 3, 1 primary study and 2 reviews), and Eastern Mediterranean Regions (*n* = 2 primary studies). Forty reviews did not specify the country where included primary studies were conducted, thus the WHO region and World Bank income region, could not be determined.

**Figure 17:**
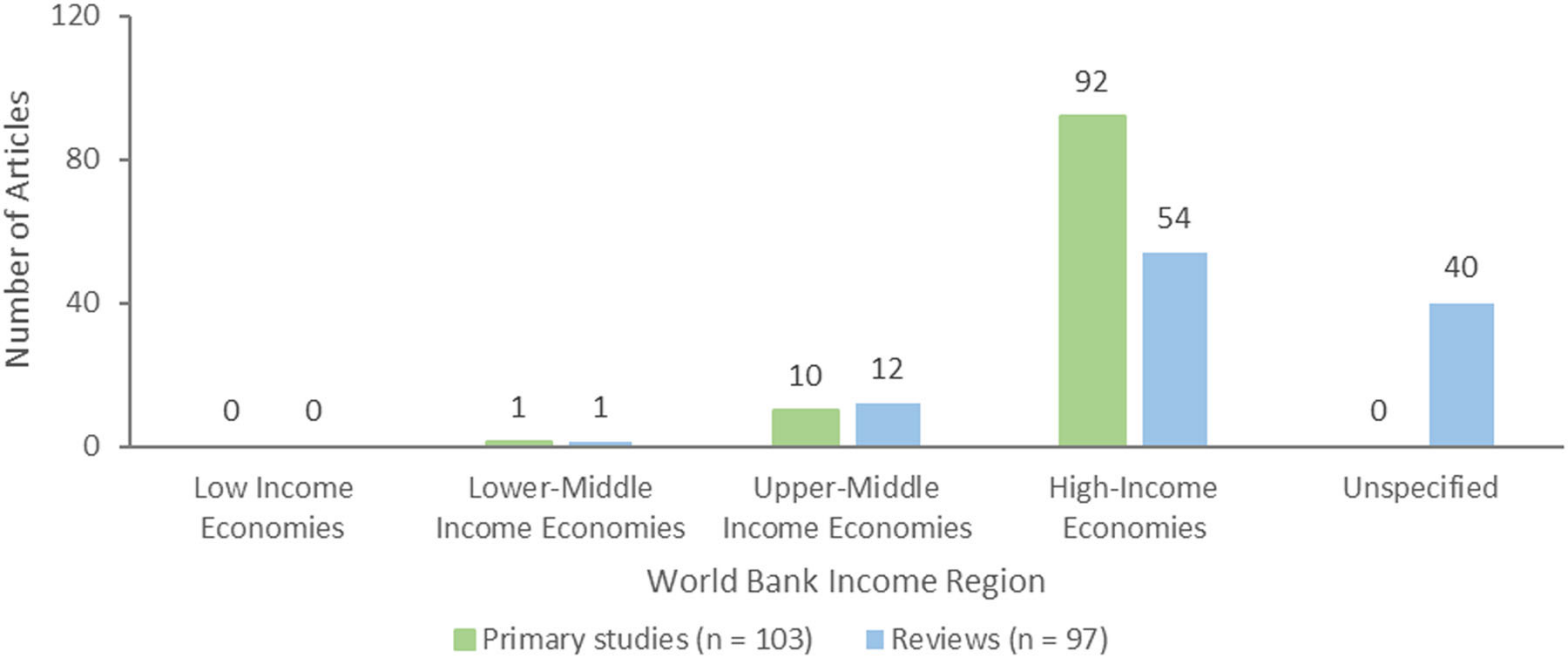
World Bank income classification.

Following the World Bank income classification, most publications were from high‐income (*n* = 146, 92 primary studies and 54 reviews) or upper‐middle‐income countries (*n* = 22, 10 primary and 12 reviews), with none from low‐income countries (Figure 17).

#### 5.4.3 Settings

**Figure 18:**
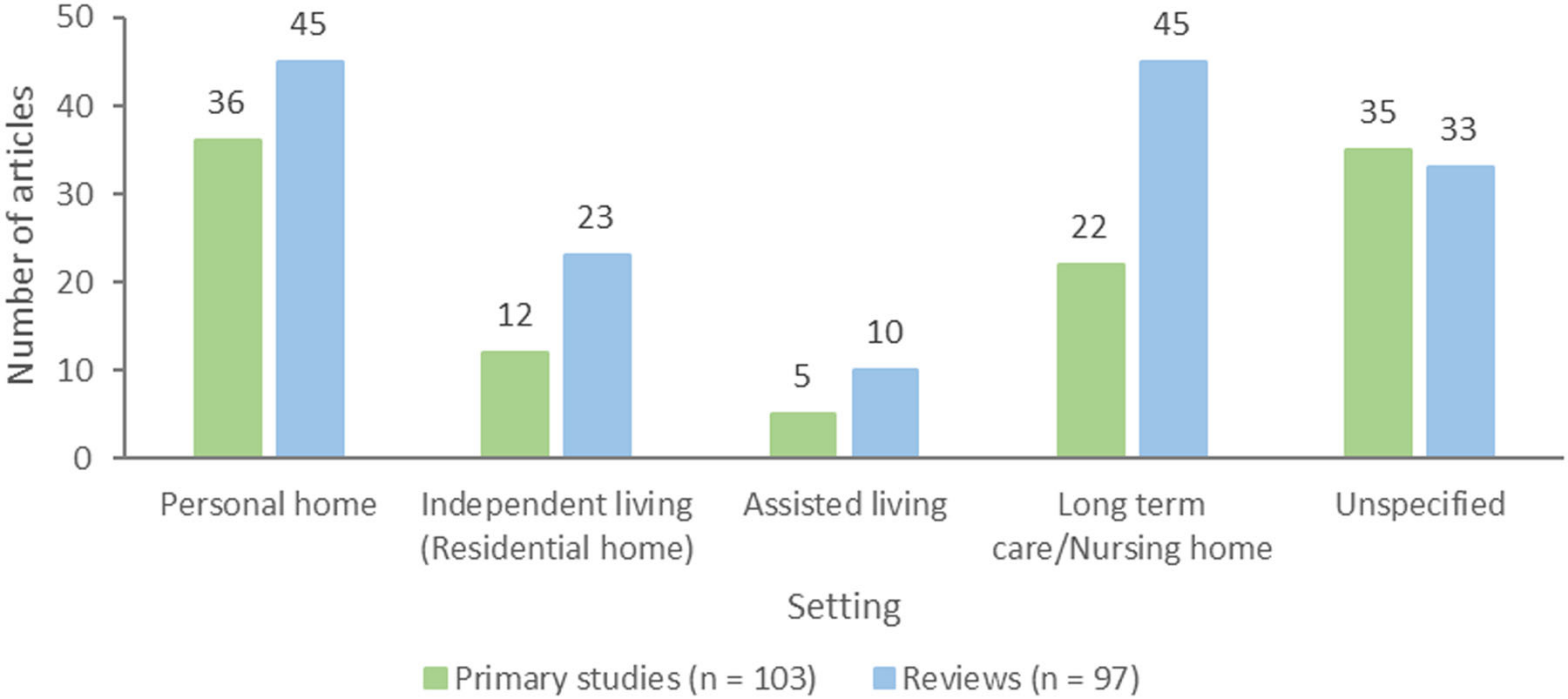
Setting.

The setting for most interventions described was the personal home of participants (*n* = 81, 36 primary studies and 45 reviews), followed by long‐term care/nursing homes (*n* = 67, 22 primary studies and 45 reviews). Few took place in independent living (*n* = 35, 12 primary studies and 23 reviews) or assisted living settings (*n* = 15, 5 primary studies and 10 reviews). Again, some publications included more than one setting for the intervention, and all reported settings were coded as such. Sixty‐eight publications (35 primary studies and 33 reviews) did not specify the setting (Figure 18).

#### 5.4.4 Participant needs

Different types of participant needs beyond social isolation and loneliness were identified. Social and emotional needs (*n* = 191, 97 primary studies and 94 reviews) were the most identified, followed by clinical/health needs (*n* = 19, 5 primary studies and 14 reviews) and caregiver support (*n* = 22, 8 primary studies and 14 reviews). Some publications reported interventions targeting more than one need, while no included article targeted participants' accommodation or financial assistance needs (Table 6).

**Table 6:** Participant needs.

Participant needs	Primary studies (*n* = 103)	Reviews (*n* = 97)
Social and emotional needs	97	94
Caregiver support	8	14
Clinical/health needs	5	14
Mobility	7	4
Purpose in Life	5	2
Personal care needs	1	5
Skills development	2	3
Meals	2	0
Respite care	1	2
Communication (language support/interpreters)	1	1
Domestic assistance	1	1
Care navigation support or task orientation	1	0
Learning	0	5
Accommodation	0	0
Financial assistance	0	0

#### 5.4.5 Participant characteristics (PROGRESS‐Plus)

**Figure 19:**
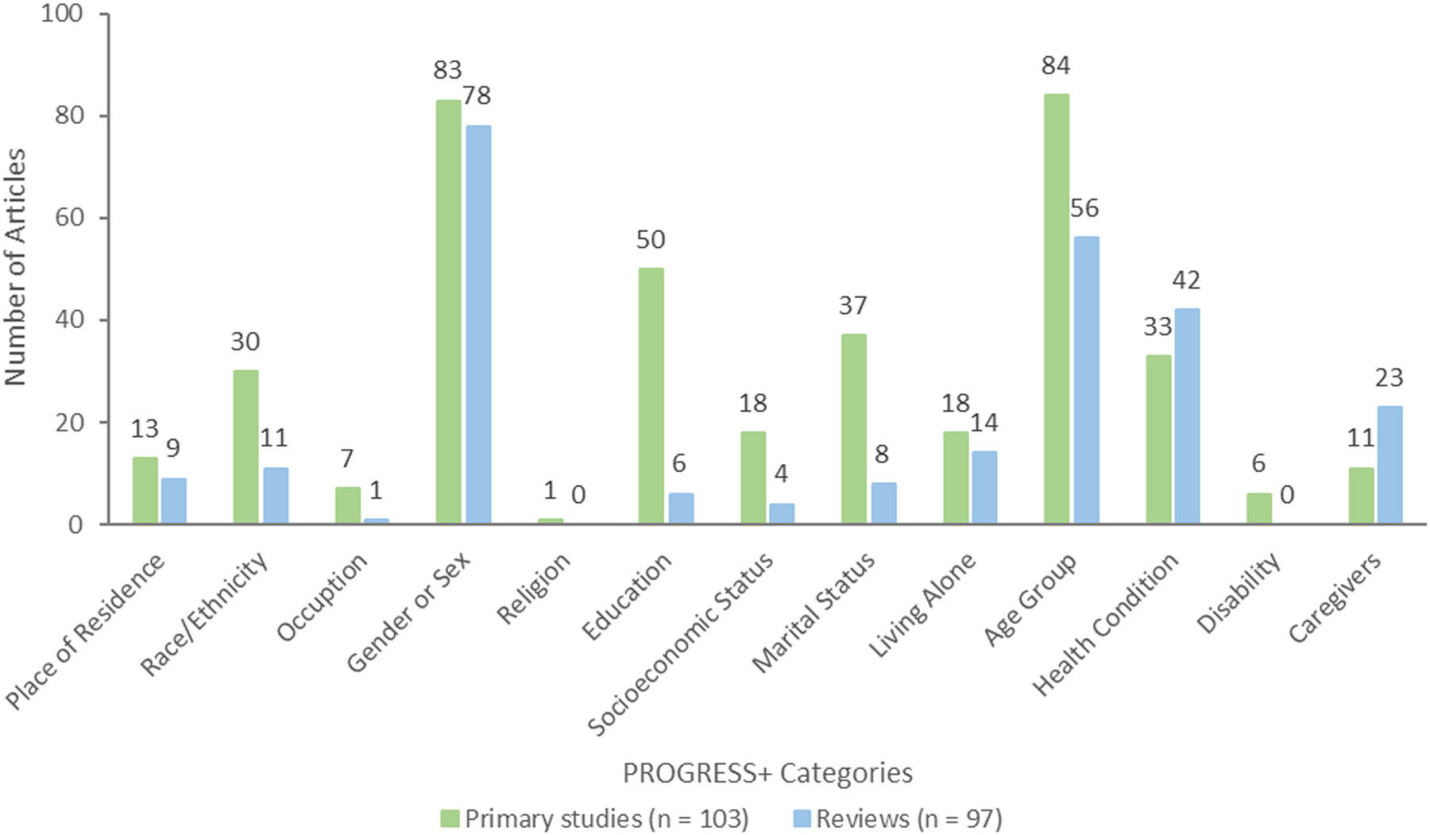
PROGRESS‐Plus categories used to describe participants.

Every PROGRESS‐Plus factor was used to describe participants in at least one included article (Figure 19). The PROGRESS acronym stands for Place of residence, Race/ethnicity, Occupation, Gender or sex, Religion, Education, Socioeconomic status, Social capital (marital status). For the purpose of this EGM, we also considered living alone, age group, disability, health condition, and being caregivers as plus factors.

Gender or sex (*n* = 161, 83 primary studies and 78 reviews) and age (*n* = 140, 84 primary studies and 56 reviews) were the most reported characteristics, while occupation (*n* = 8, 7 primary studies and 1 review), and religion (*n* = 1 primary study) were the least reported.

Of the 161 articles specifying gender or sex, most included male and female participants. Two reviews included some studies with only male participants and 8 reviews included some studies with only female participants; 4 primary studies included only female participants, and gender or sex was unspecified in 39 articles (20 primary studies and 19 reviews). No articles included individuals from the LGBTQIA2S+ (lesbian, gay, bisexual, transgender, queer [or sometimes questioning], and two‐spirited) community.

**Figure 20:**
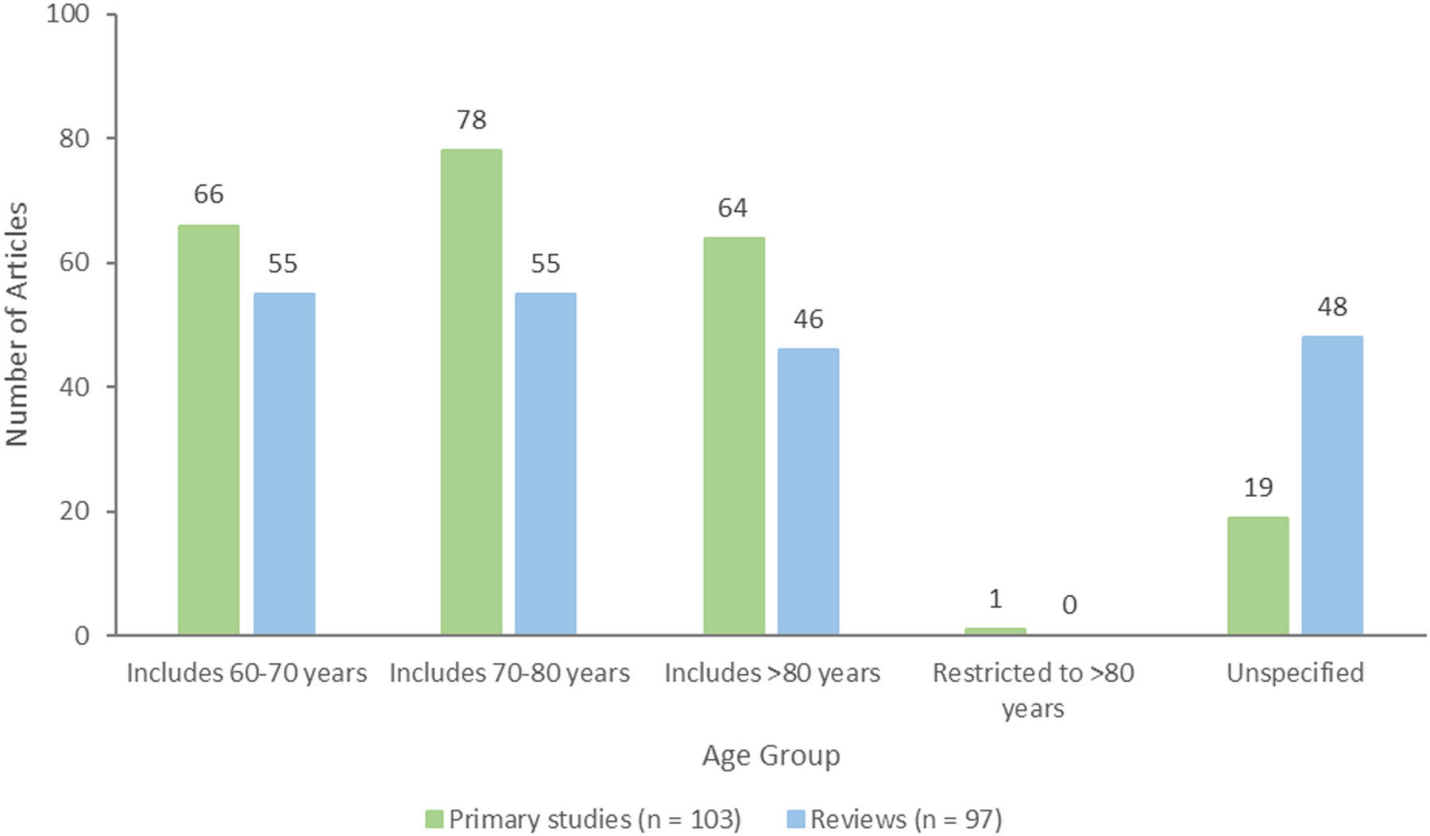
Participant age groups.

Of the articles that reported age, the 70–80‐year‐old range was most reported (*n* = 133, 78 primary studies and 55 reviews), and the age range restricted to older than 80 was least reported (*n* = 1 primary study). Age of participants was not specified in 67 articles (19 primary studies and 48 reviews) (Figure 20).

#### 5.4.6 Health condition of participants

**Figure 21:**
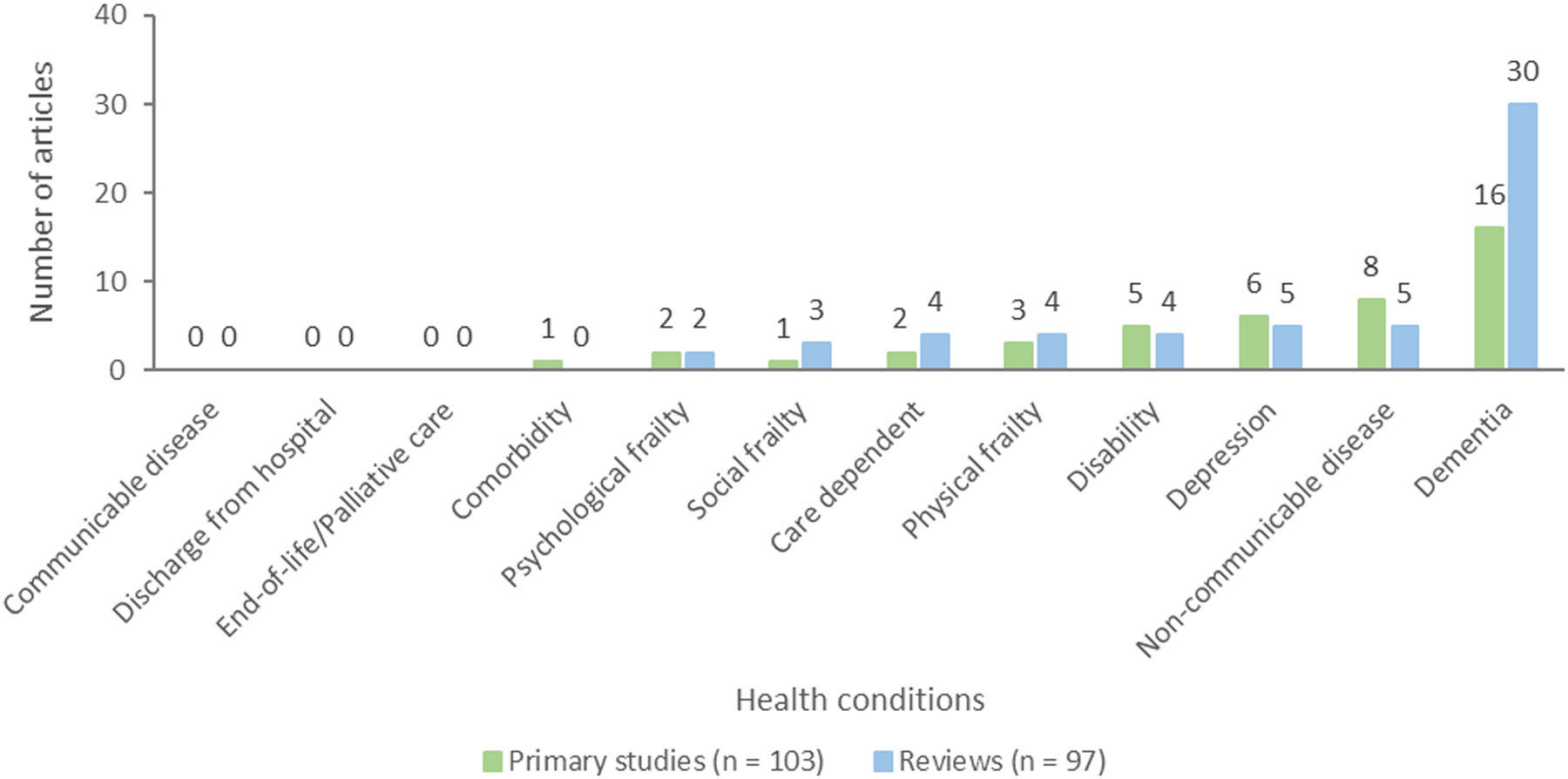
Health conditions of participants.

The health condition of participants was reported in 75 articles (33 primary studies and 42 reviews). Dementia was most common (*n* = 46, 16 primary studies and 30 reviews), followed by other non‐communicable diseases (*n* = 13, 8 primary studies and 5 reviews). Included articles did not report on communicable diseases, discharge from hospital, and end‐of‐life/palliative care (Figure 21).

#### 5.4.7 Equity analysis

**Figure 22:**
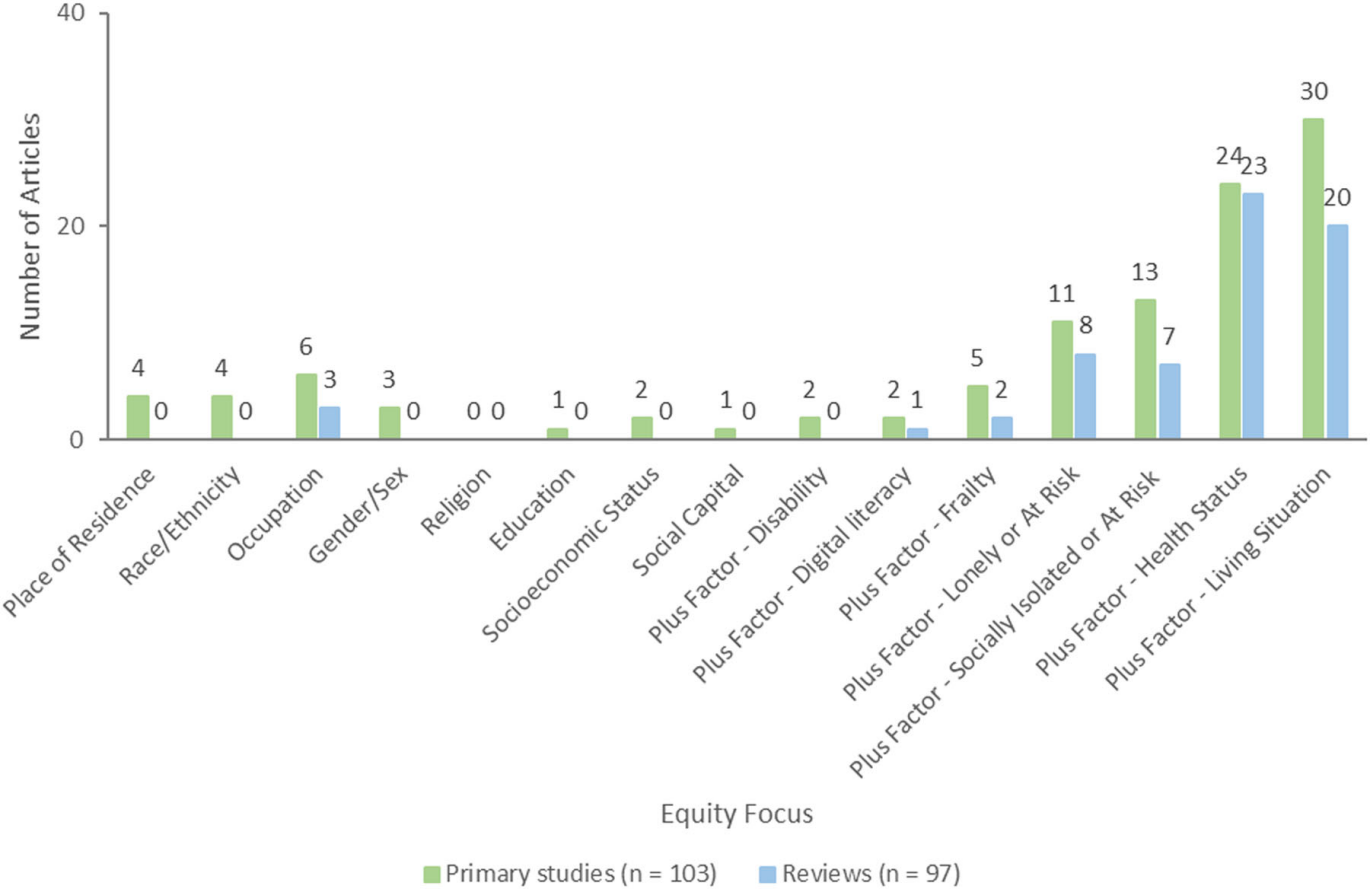
Equity focus of vulnerability across PROGRESS‐Plus factors.

Of the 200 included articles, 78 primary studies and 52 reviews focused on recruiting older adults vulnerable across one or more PROGRESS‐Plus factors (Figure 22). Most included articles focused on living situation, that is, older adults living alone or in a nursing home (*n* = 50, 30 primary studies and 20 reviews), health status (including dementia, depression and other chronic conditions) of older adults (*n* = 47, 24 primary studies and 23 reviews), socially isolated older adults or at risk of social isolation (*n* = 20, 13 primary studies and 7 reviews), or lonely older adults or at risk of loneliness (*n* = 19, 11 primary studies and 8 reviews). Few articles focused on other equity relevant factors including occupation (*n* = 9, 6 primary studies and 3 reviews), frailty (*n* = 7, 5 primary studies and 2 reviews), ethnicity (*n* = 4 primary studies), place of residence, urban or rural (*n* = 4 primary studies), gender/sex (*n* = 3 primary studies), digital literacy (*n* = 3, 2 primary studies and 1 review), disability (*n* = 2 primary studies), socioeconomic status (*n* = 2 primary studies), education level (*n* = 1 primary study), or social capital (*n* = 1 primary study). No articles focused on religion.

Very few studies focused on multiple vulnerability categories, for example, frail and socially isolated older adults (*n* = 1), rural isolated family caregivers (*n* = 1), Chinese socially isolated immigrants (*n* = 1), low‐income women living alone (*n* = 1), populations at risk of both social isolation and loneliness (*n* = 9).

**Figure 23:**
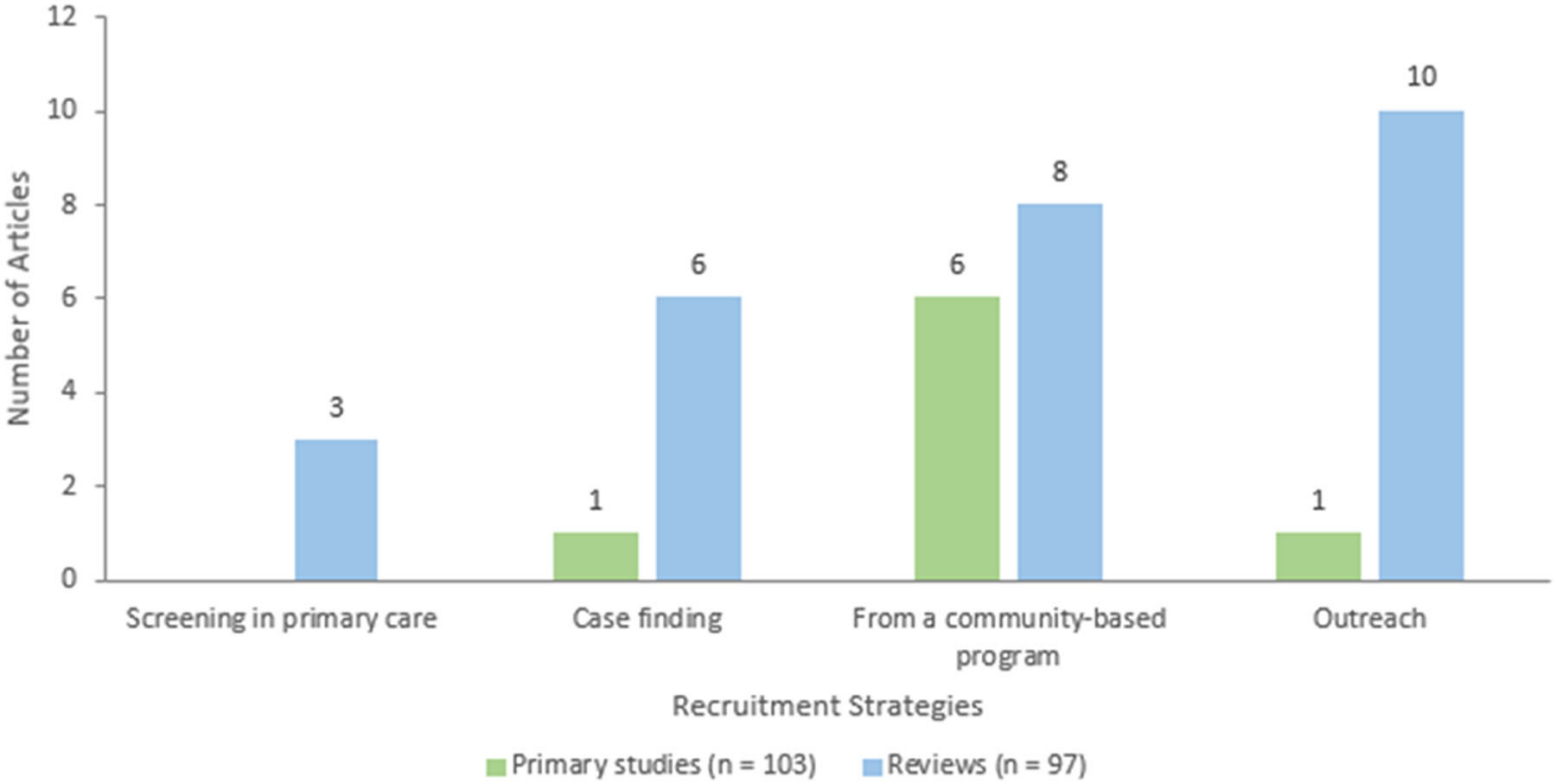
Recruitment strategies for at‐risk populations.

Very few studies described how at‐risk populations were recruited (Figure 23). They were identified through outreach (*n* = 11, 10 primary studies and 1 review), community‐based programs (*n* = 11, 8 primary studies and 3 reviews), case finding (*n* = 7, 6 primary studies and 1 review), or through screening in primary care (*n* = 3 primary studies). One primary study used all four approaches to identify at‐risk populations.

**Figure 24:**
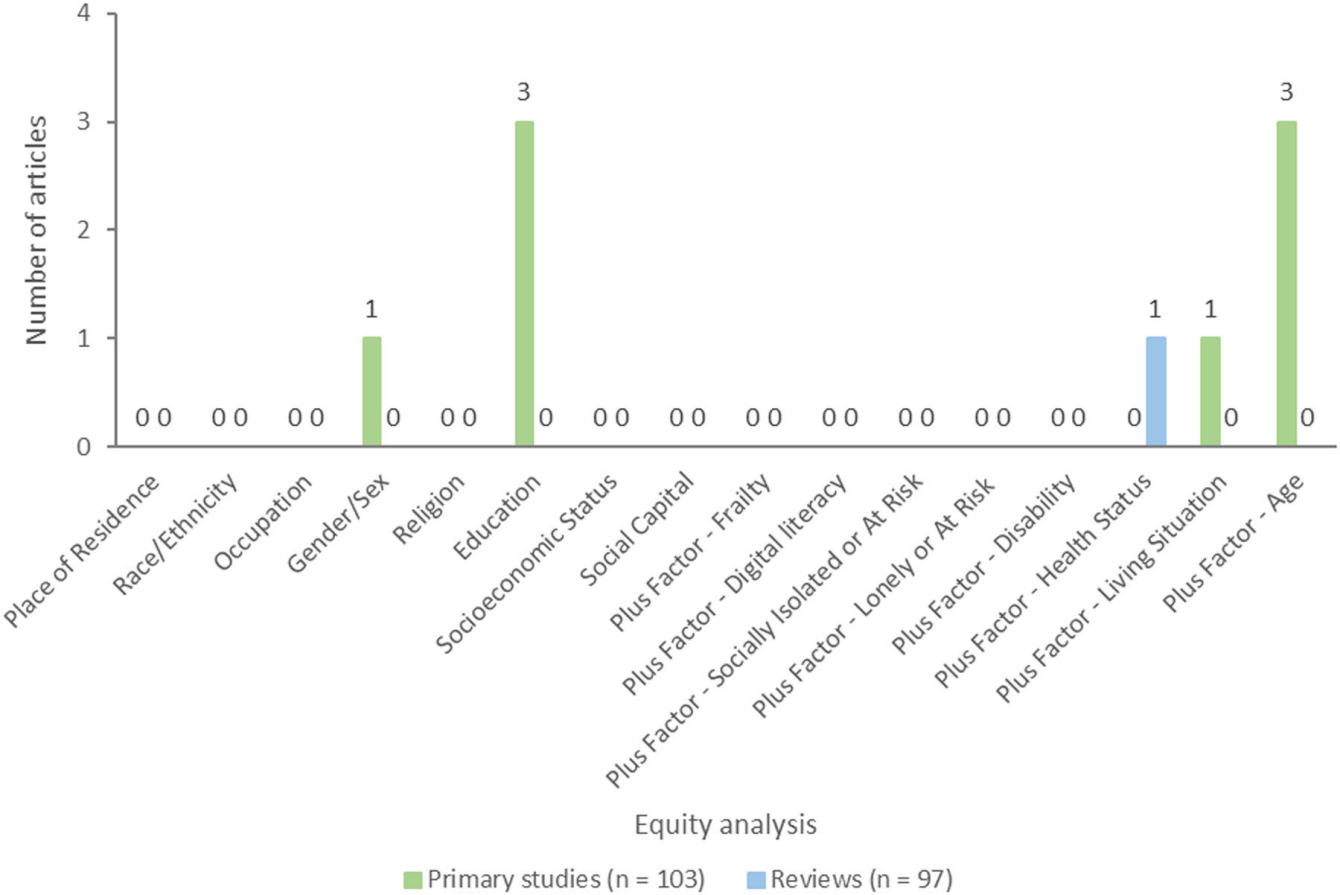
Equity analyses to assess differences in effects across any PROGRESS‐Plus factor.

We also assessed whether studies analyzed differences in effects for populations experiencing inequities across PROGRESS‐Plus categories. Despite the large number of articles that considered at least one equity focus, only five articles (4 primary studies and 1 review) assessed the differences in effects across any PROGRESS‐Plus factor including gender or sex (*n* = 1 study), education level (*n* = 3 primary studies), age (*n* = 3 primary studies), living situation (*n* = 1 primary study), and health status (*n* = 1 review). No equity analyses were completed for place of residence, race/ethnicity, occupation, religion, socioeconomic status, social capital, frailty, digital literacy, being socially isolated or at risk, being lonely or at risk, or disability (Figure 24).

#### 5.4.8 Stakeholder feedback on framework and draft map

We surveyed 30 people and got 10 (33%) responses. Eight of the 10 (80%) people found that the intervention categories made sense, one (1%) person was neutral and one (1%) disagreed with the category social skills training. We decided to keep social skills training based on the Masi framework (Masi, 2011).

Six (60%) people found the outcome categories made sense, three (30%) were neutral and one (1%) disagreed with including depression/anxiety, quality of life and confidence level as outcomes. We kept these outcomes because they were indicators of social connections (Elder, 2012; Windle, 2012) and were reported in many included articles – 112/200 studies measured anxiety/depression, 90/200 studies measured quality of life, and 23/200 studies measured confidence. Although six (60%) people easily found the information they were looking for on the draft map and one (16.67%) person was neutral, they provided helpful suggestions for knowledge translation activities and improving the usability of the map such as creating a short video of how to use the map.

## 6 DISCUSSION

### 6.1 Summary of main results

This evidence and gap map has 200 included articles and there are almost as many primary studies (*n* = 103) as reviews (*n* = 97), with most conducted in the Americas, the European and Western Pacific regions. The high ratio of reviews to primary studies indicates a surge in research with over 25% of the reviews conducted since the COVID‐19 pandemic but most reviews are of critically low quality.

The evidence is unevenly distributed across intervention and outcome subcategories with a similar trend across primary studies and reviews. The majority of included primary studies and reviews assessed digital interventions to enhance social interactions with family and friends and the community via videoconferencing and telephone calls. Digital interventions to enhance social support, particularly socially assistive robots, and virtual pets were the next most assessed subcategory of interventions. The social cognitive training strategy was the least assessed of the four intervention strategies although it is considered the most effective (Mann, 2017; Masi, 2011).

The most assessed outcomes were reducing loneliness and depression and improving the quality of life of older adults. Only six reviews reported adverse events and two on‐going primary studies planned to report adverse effects. The most reported age range was 70–80 years old and most common needs addressed were social and emotional needs.

Although many primary studies and reviews focused on recruiting older adults vulnerable across one or more PROGRESS‐Plus factors, very few described how at‐risk populations were recruited or conducted any equity analysis to assess differences in effects for populations experiencing inequities across PROGRESS‐Plus categories.

### 6.2 Areas of major gaps in the evidence

Major gaps were identified in outcome measures including adverse effects, community level outcomes and process indicators. Only six reviews reported adverse effects. No included studies or reviews assessed affordability or digital divide, which refers to disparities in access to technological interventions, although three primary studies and three reviews discussed the value of accessibility of digital interventions and one of the reviews discussed the barriers caused by digital divide. There were no primary studies or reviews conducted in low‐income countries. Only one primary study restricted participants to 80 years and older and no study or review included participants from the LGBTQIA2S+ community.

### 6.3 Potential biases in the mapping process

The search strategy was comprehensive and covered published literature until May 16, 2021. We used rigorous methods including duplicate screening of all the identified records and reference lists of included reviews and assessment of methodological quality of reviews. Since our research question is about the effectiveness of digital interventions for social isolation or loneliness, we included only study designs that are appropriate for assessing effectiveness. We did not use outcomes as eligibility criteria. We included studies that assessed the effects of digital interventions on social isolation and loneliness as well as other indicators of social connections such as quality of life, depression.

With the growing research in this area, we may have missed more recent primary studies and reviews. Although there were no language restrictions applied, we may have missed studies and reviews published in non‐English language since mainly English databases were searched.

Mapping the interventions into mutually exclusive categories in the evidence and gap map was challenging due to the lack of consistent terminology and a standardized framework for the classification of interventions for social isolation and loneliness.

### 6.4 Limitations of the EGM

The map does not include evidence from studies with no control groups or qualitative research. Although these studies might provide useful information about digital interventions for social isolation and loneliness in older adults, they do not assess the effectiveness of interventions.

Most studies and reviews do not focus on reaching the most vulnerable older adults including specific groups such as lonely/socially isolated or at‐risk, low socioeconomic status, low literacy, ethnic minority groups, LGBTQIA2S+ community or populations in low‐income countries where access to digital technology is limited. No included studies or reviews assessed affordability or accessibility of digital interventions, but three primary studies and three reviews discussed the value of accessibility of digital interventions and one of the reviews discussed the barriers caused by digital divide.

### 6.5 Stakeholder engagement throughout the EGM process

Citizens were involved in the development of this EGM, and they provided constructive and valuable feedback that was incorporated in the process. Only 29% of stakeholders including citizens provided feedback on the framework and draft map.

Although one stakeholder disagreed with social skills training as an intervention category, we decided to keep it since it is included in the Masi framework as interventions that focused on improving interpersonal communication skills (Masi, 2011). One stakeholder disagreed with including depression/anxiety, quality of life and confidence level as outcomes but these outcomes have been described as indicators of social connections (Elder, 2012; Windle, 2012) and were reported in many included articles.

## 7 AUTHORS' CONCLUSIONS

### 7.1 Implications for research, practice and/or policy

Social isolation and loneliness are growing public health concerns, which have been highlighted during the COVID‐19 pandemic. This evidence and gap map shows available evidence on effectiveness of digital interventions for reducing social isolation or loneliness in older adults. Over 70% of the systematic reviews have critically low quality, 2% have high quality, and 25% have been published since the pandemic. Clusters of evidence of critically low quality exist mainly for digital interventions to enhance social interactions with family and friends and the community via videoconferencing and telephone calls as well as digital interventions to enhance social support, particularly socially assistive robots, and virtual pets. Most of the digital interventions were focused on reducing loneliness and depression and improving quality of life of older adults.

Major gaps were lack of evidence on equity analysis, community level outcomes and process indicators. Affordability and the digital divide were not assessed in any of the included studies or reviews although barriers caused by the digital divide may increase health inequities and have been key challenges for organizations and governments concerning digital interventions during the COVID‐19 pandemic restrictions. Many studies did not focus on reaching the most vulnerable population groups. The evidence is predominantly from high‐income countries with none in low‐income countries. Other challenges faced by organizations and governments are related to the adverse effects of digital interventions such as privacy and ethical issues. However, very few primary studies and reviews have assessed adverse effects.

This evidence and gap map is the starting point for building the evidence architecture and will guide a future research agenda to standardize definitions of concepts, intervention characteristics, and outcome measurements as well as identify which digital interventions are effective for reducing social isolation and loneliness in older adults taking into consideration their specific needs and contexts.

Although there is proliferation of evidence in recent years, it is unevenly distributed and the systematic reviews are of poor quality highlighting the need for high quality research. Registration of reviews may help to reduce duplication. This map can guide researchers and funders to consider areas of major gaps as priorities for further research.

## SOURCES OF SUPPORT

**Internal sources**

- None, Other

**External sources**

- World Health Organization, Switzerland

WHO funding – Purchase Order Number: 202666968

Feedback

## CONTRIBUTIONS OF AUTHORS

Content: PH, CM, VW, EG, RS, SB, NH, EB, DK

EGM methods: VW, EG, VB, EB, DK, SD, RD, NE, TH, AW, EBev, PD, JH

Information retrieval: DS

All authors read and approved the review.

## DECLARATIONS OF INTEREST

Vivian Welch is editor in chief of the Campbell Collaboration. The editorial process was handled by an independent editor and VW had no input in the editorial process or decisions.

Elisabeth Boulton and Dylan Kneale are joint lead authors of a previous systematic review of systematic reviews which may be eligible for inclusion for the map.

Elizabeth Ghogomu, Victoria Barbeau, Sierra Dowling, Rebecca Doyle, Ella Beveridge, Payaam Desai, Jimmy Huang, Nour Elmestekawy, Tarannum Hussain, Arpana Wadhwani, Sabrina Boutin, Niobe Haitas, Douglas M. Salzwedel, Roger Simard, Paul Hebert, Christopher Mikton have no conflicts of interest.

## PLANS FOR UPDATING THE EGM

The EGM will be updated every 2 years.

## DIFFERENCES BETWEEN PROTOCOL AND REVIEW

We simplified the conceptual framework in the review.

## PUBLISHED NOTES

**Characteristics of studies**

Characteristics of included studies

**Table:** 

Abbott 2019
**Notes**
Risk of bias table
Abdi 2017
**Notes**
Risk of bias table
Abdi 2018
**Notes**
Risk of bias table
Abou 2020
**Notes**
Risk of bias table
Aggarwal 2020
**Notes**
Risk of bias table
Ali 2020
**Notes**
Risk of bias table
Alves 2020
**Notes**
Risk of bias table
Archer 2019
**Notes**
Risk of bias table
Baez 2017
**Notes**
Risk of bias table
Baker 2018
**Notes**
Risk of bias table
Ballesteros 2014
**Notes**
Risk of bias table
Banks 2008
**Notes**
Risk of bias table
Bedaf 2015
**Notes**
Risk of bias table
Beentjes 2020
**Notes**
Risk of bias table
Bemelmans 2012
**Notes**
Risk of bias table
Beneito‐Montagut 2018
**Notes**
Risk of bias table
Bermeja 2018
**Notes**
Risk of bias table
Bethell 2019
**Notes**
Risk of bias table
Boekhout 2021
**Notes**
Risk of bias table
Bornemann 2014
**Notes**
Risk of bias table
Boston 2020
**Notes**
Risk of bias table
Brodbeck 2019
**Notes**
Risk of bias table
Bruce 2020
**Notes**
Risk of bias table
Buitenweg 2019
**Notes**
Risk of bias table
Cacciata 2019
**Notes**
Risk of bias table
Campos 2016
**Notes**
Risk of bias table
Casanova 2021
**Notes**
Risk of bias table
Cattan 2005
**Notes**
Risk of bias table
Chang 2013
**Notes**
Risk of bias table
Chen 2016
**Notes**
Risk of bias table
Chen 2018
**Notes**
Risk of bias table
Chipps 2017
**Notes**
Risk of bias table
Chiu 2019
**Notes**
Risk of bias table
Choi 2012
**Notes**
Risk of bias table
Choi 2020
**Notes**
Risk of bias table
Choi 2020a
**Notes**
Risk of bias table
Choi 2021
**Notes**
Risk of bias table
Chua 2013
**Notes**
Risk of bias table
Cohen‐Mansfield 2015
**Notes**
Risk of bias table
Coll‐Planas 2017
**Notes**
Risk of bias table
Courtin 2015
**Notes**
Risk of bias table
Czaja 2015
**Notes**
Risk of bias table
Czaja 2018
**Notes**
Risk of bias table
D'Cunha 2021
**Notes**
Risk of bias table
Da‐Eun 2018
**Notes**
Risk of bias table
Damant 2017
**Notes**
Risk of bias table
Dequanter 2020
**Notes**
Risk of bias table
Dermody 2020
**Notes**
Risk of bias table
Dichter 2020
**Notes**
Risk of bias table
Dickens 2011 a
**Notes**
Risk of bias table
Dodge 2015
**Notes**
Risk of bias table
Dodge 2018
**Notes**
Risk of bias table
Duggleby 2020
**Notes**
Risk of bias table
El 2020
**Notes**
Risk of bias table
Engelbrecht 2015
**Notes**
Risk of bias table
**Etxeberria**
**Notes**
Risk of bias table
Fakoya 2020
**Notes**
Risk of bias table
Feng 2020
**Notes**
Risk of bias table
Ferreira 2015
**Notes**
Risk of bias table
Fields 2021
**Notes**
Risk of bias table
Fokkema 2007
**Notes**
Risk of bias table
Forsman 2018
**Notes**
Risk of bias table
Francis 2019
**Notes**
Risk of bias table
Gardiner 2018
**Notes**
Risk of bias table
Godwin 2013
**Notes**
Risk of bias table
Gongora 2019
**Notes**
Risk of bias table
Gustafson 2019
**Notes**
Risk of bias table
Gustafson 2021
**Notes**
Risk of bias table
Gustafson 2021a
**Notes**
Risk of bias table
Haase 2021
**Notes**
Risk of bias table
Hagan 2014
**Notes**
Risk of bias table
Hartke 2003
**Notes**
Risk of bias table
Health Quality Ontario 2008
**Notes**
Risk of bias table
Heller 1991
**Notes**
Risk of bias table
Hernández‐Ascanio 2020
**Notes**
Risk of bias table
Heyn 2001
**Notes**
Risk of bias table
Hicken 2017
**Notes**
Risk of bias table
Hoang 2021
**Notes**
Risk of bias table
Hopwood 2018
**Notes**
Risk of bias table
Hung 2019
**Notes**
Risk of bias table
Hung 2021
**Notes**
Risk of bias table
Ibarra 2020
**Notes**
Risk of bias table
Ibrahim 2021
**Notes**
Risk of bias table
Iman 2015
**Notes**
Risk of bias table
Isabet 2021
**Notes**
Risk of bias table
Jackson 2016
**Notes**
Risk of bias table
Jarvis 2019
**Notes**
Risk of bias table
Jarvis 2020
**Notes**
Risk of bias table
Jones 2015
**Notes**
Risk of bias table
Jones 2020
**Notes**
Risk of bias table
Kachouie 2014
**Notes**
Risk of bias table
Kachouie 2017
**Notes**
Risk of bias table
Kahlon 2021
**Notes**
Risk of bias table
Kamalpour 2020
**Notes**
Risk of bias table
kazazi 2021
**Notes**
Risk of bias table
Keogh 2014
**Notes**
Risk of bias table
Khosravi 2016
**Notes**
Risk of bias table
Khosravi 2016a
**Notes**
Risk of bias table
Koh 2021
**Notes**
Risk of bias table
Koh 2021a
**Notes**
Risk of bias table
Koo 2019
**Notes**
Risk of bias table
Kramer 2021
**Notes**
Risk of bias table
Kubra 2020
**Notes**
Risk of bias table
Lady Lady Davis Institute 2021
**Notes**
Risk of bias table
Laganà 2013
**Notes**
Risk of bias table
Lai 2020
**Notes**
Risk of bias table
Lappalainen 2021
**Notes**
Risk of bias table
Larsson 2016
**Notes**
Risk of bias table
Lauriks 2007
**Notes**
Risk of bias table
Lawson Health Research Institute 2021
**Notes**
Risk of bias table
Lee 2021
**Notes**
Risk of bias table
Li 2017
**Notes**
Risk of bias table
Li 2018
**Notes**
Risk of bias table
Liang 2017
**Notes**
Risk of bias table
Lin 2020
**Notes**
Risk of bias table
Lins 2014
**Notes**
Risk of bias table
Lopez‐Hartmann 2012
**Notes**
Risk of bias table
Masi 2010
**Notes**
Risk of bias table
Matson 2019
**Notes**
Risk of bias table
Matz‐Costa 2018
**Notes**
Risk of bias table
McKechnie 2014
**Notes**
Risk of bias table
Mikkelsen 2019
**Notes**
Risk of bias table
Milbury 2020
**Notes**
Risk of bias table
Miller 2014
**Notes**
Risk of bias table
Mittelman 2006
**Notes**
Risk of bias table
Montana 2020
**Notes**
Risk of bias table
Morris 2014
**Notes**
Risk of bias table
Morton 2018
**Notes**
Risk of bias table
Mountain 2014
**Notes**
Risk of bias table
Moyle 2017
**Notes**
Risk of bias table
Myhre 2015
**Notes**
Risk of bias table
Myhre 2017
**Notes**
Risk of bias table
Neal 2021
**Notes**
Risk of bias table
Nef 2013
**Notes**
Risk of bias table
New Study 2
**Notes**
Risk of bias table
Nijhof 2009
**Notes**
Risk of bias table
Nikitina 2018
**Notes**
Risk of bias table
Nilsson 2021
**Notes**
Risk of bias table
Noone 2020
**Notes**
Risk of bias table
O'Rourke 2018
**Notes**
Risk of bias table
Ollevier 2020
**Notes**
Risk of bias table
Papadopoulos 2021
**Notes**
Risk of bias table
Parkinson 2018
**Notes**
Risk of bias table
Pedrozo 2019
**Notes**
Risk of bias table
Pepin 2019
**Notes**
Risk of bias table
Pereira 2018
**Notes**
Risk of bias table
Peres 2021
**Notes**
Risk of bias table
Pinto‐Bruno 2017
**Notes**
Risk of bias table
Portz 2017
**Notes**
Risk of bias table
Poscia 2018
**Notes**
Risk of bias table
Pu 2019
**Notes**
Risk of bias table
Queiros 2017
**Notes**
Risk of bias table
Quinn 2021
**Notes**
Risk of bias table
Rainero 2021
**Notes**
Risk of bias table
Rienzo 2019
**Notes**
Risk of bias table
Robbins 2019
**Notes**
Risk of bias table
Robinson 2013
**Notes**
Risk of bias table
Robinson 2019
**Notes**
Risk of bias table
Ronzi 2018
**Notes**
Risk of bias table
Roth 2014
**Notes**
Risk of bias table
**Salehi**
**Notes**
Risk of bias table
Schulz 2002
**Notes**
Risk of bias table
Schwindenhammer 2014
**Notes**
Risk of bias table
Sciamanna 2021
**Notes**
Risk of bias table
Scoglio 2019
**Notes**
Risk of bias table
Shah 2021
**Notes**
Risk of bias table
Shapira 2007
**Notes**
Risk of bias table
Shapira 2021
**Notes**
Risk of bias table
Shapira 2021a
**Notes**
Risk of bias table
Shishehgar 2018
**Notes**
Risk of bias table
Shishehgar 2019
**Notes**
Risk of bias table
Smallfield 2018
**Notes**
Risk of bias table
Smith 2006
**Notes**
Risk of bias table
Smith 2011
**Notes**
Risk of bias table
Smith 2017
**Notes**
Risk of bias table
Song 2009
**Notes**
Risk of bias table
Song 2019
**Notes**
Risk of bias table
Sosa 2012
**Notes**
Risk of bias table
Su 2020
**Notes**
Risk of bias table
Tanaka 2012
**Notes**
Risk of bias table
Thodberg 2016
**Notes**
Risk of bias table
Tomasino 2017
**Notes**
Risk of bias table
Tsai 2010
**Notes**
Risk of bias table
Tsai 2011
**Notes**
Risk of bias table
Tsai 2020
**Notes**
Risk of bias table
Tsai 2020a
**Notes**
Risk of bias table
Tyack 2017
**Notes**
Risk of bias table
University of Texas at Austin 2020
**Notes**
Risk of bias table
Uppsala University 2020
**Notes**
Risk of bias table
Van Den Heuvel 2019
**Notes**
Risk of bias table
Van Houwelingen‐Snippe 2021
**Notes**
Risk of bias table
Vanova 2018
**Notes**
Risk of bias table
VanRavenstein 2020
**Notes**
Risk of bias table
Vazquez 2019
**Notes**
Risk of bias table
Wasilewski 2017
**Notes**
Risk of bias table
White 1999
**Notes**
Risk of bias table
White 2002
**Notes**
Risk of bias table
Woodward 2011
**Notes**
Risk of bias table
Zaccaria 2020
**Notes**
Risk of bias table
Zhao 2020
Notes
Risk of bias table

**Characteristics of excluded studies**

**Table:** 

Bolle 2015	
**Reason for exclusion**	Evidence/aim (not to reduce social isolation or loneliness)
Burkow 2015	
**Reason for exclusion**	Target population (included <60 years old, and not disaggregated by age)
Clarkson 2018	
**Reason for exclusion**	Evidence/aim (not to reduce social isolation or loneliness)
Cooper 2014	
**Reason for exclusion**	Target population (included <60 years old, and not disaggregated by age)
Dam 2017	
**Reason for exclusion**	Target population (included <60 years old, and not disaggregated by age)
Dickens 2011	
**Reason for exclusion**	Intervention (not digital)
Erfani 2018	
**Reason for exclusion**	Target population (included <60 years old, and not disaggregated by age)
Fan 2016	
**Reason for exclusion**	Study design (not a systematic review of effectiveness)
Forsman 2017	
**Reason for exclusion**	Study design (not a systematic review of effectiveness)
Gine‐Garriga 2017	
**Reason for exclusion**	Intervention (not digital)
Gorenko 2021	
**Reason for exclusion**	Study design (not a systematic review of effectiveness)
Jones 2019	
**Reason for exclusion**	Intervention (not digital)
Lara 2016	
**Reason for exclusion**	Target population (included <60 years old, and not disaggregated by age)
Nicholson 2012	
**Reason for exclusion**	Intervention (not digital)
Nijman 2019	
**Reason for exclusion**	Target population (included <60 years old, and not disaggregated by age)
Perkins 2012	
**Reason for exclusion**	Study design (not a non‐comparative primary study)
Preston 2019	
**Reason for exclusion**	Study design (not a non‐comparative primary study)
Rebollar 2015	
**Reason for exclusion**	Study design (not a non‐comparative primary study)
Selak 2019	
**Reason for exclusion**	Study design (not a systematic review of effectiveness)
Sumner 2021	
**Reason for exclusion**	Evidence/aim (not to reduce social isolation or loneliness)
Toh 2016	
**Reason for exclusion**	Intervention (not digital)
Winterton 2011	
**Reason for exclusion**	Study design (not a non‐comparative primary study)
Zeppegno 2018	
**Reason for exclusion**	Intervention (not digital)

## Supporting information

Supporting information.

Supporting information.
