# Supplementary material for: Digital interventions to reduce social isolation and loneliness in older adults: An evidence and gap map
Source: Campbell Syst Rev. 2023 Nov 27;19(4):e1369. doi: 10.1002/cl2.1369 (PMC10681039; doi:10.1002/cl2.1369)
Supplement: Supplementary file 1 — Supporting information. [file CL2-19-e1369-s001.docx]

Appendices

## 1 Glossary of key concepts

### Key concepts related to intervention categories

**Interventions to improve social skills**: Interventions that focus on training in or education on one’s social skills, such as conversational ability. They aim to enable individuals to form and maintain meaningful relationships (Mann 2017; Masi 2011).

**Social skills** are any skills facilitating interaction and communication with others. They are socially acceptable learned behaviours that enable a person to interact with others in ways that elicit positive responses and assist in avoiding negative responses (Little 2017)**.**

**Interventions to enhance social support**: interventions where people are offered support (regular contacts, care, or companionship) and guidance in finding and attending new activities or groups. They aim to help individuals make and maintain social connections (Mann 2017; Masi 2011).

**Social support** is the actual or perceived availability of resources (e.g., informational, tangible, emotional help) from others, typically one’s social network (Berkman 2000; Bethell 2019; Kelly 2017; Mann 2017).

**Interventions to enhance social interactions**: interventions that focus on improving the quality of relationships and increase opportunities for social interactions. They aim to promote engagement (Masi 2011).

Cognitive behavioural therapy aims to change negative thinking

Cognitive behavioural coaching helps individuals reach desired goals

**Interventions for social cognitive training**: interventions that focus on changing negative thinking and feelings about social relationships. They aim to change behaviors, reduce maladaptive cognitions and increase social connections (Mann 2017; Masi 2011).

### Key concepts related to outcomes

**Social connection**: encompasses the structural, functional, and quality aspects of how individuals connect to each other (Donovan 2020; Zavaleta 2014).

**Social network**: denotes the number and type of social relationships with people (individuals or groups) (Berkman 2000; Bethell 2019; Kelly 2017; Mann 2017).

**Social support**: the actual or perceived availability of resources (e.g., informational, tangible, emotional help) from others, typically one’s social network (Berkman 2000; Bethell 2019; Kelly 2017; Mann 2017) to help them adapt to change and cope with stress (Elder 2012).

**Social engagement**: reflects participation in meaningful activities with others (Berkman 2000; Bethell 2019; Elder 2012).

**Social cohesion**: is the neighborhood-based resource of mutual community trust and solidarity (Elder 2012).

**Social capital**: refers to a series of resources that individuals earn as a result of their membership in social networks, and the features of those networks that facilitate individual or collective actions such as interpersonal trust, reciprocity and mutual aid (Bethell 2019; Elder 2012; Mann 2017; Zavaleta 2014).

**Digital divide**: refers to disparities in access to technological interventions (e.g., smartphones, computers and the internet) which may be due to lack of affordability or access to technology, broadband or Wi-Fi, data poverty or geographic location, lack of digital skills and the confidence to access online services and support (Budd 2020; UCLG 2020; Watts 2020).

## 2 Search strategies

**Database: Ovid MEDLINE(R) ALL <1946 to May 14, 2021>**

Search Date: 16 May 2021

Yield: 4685

--------------------------------------------------------------------------------

1 exp aged/ or middle aged/ (5245060)

2 (ageing or aging or care home resident* or community-dwelling or elder* or frail* or geriatric* or “late life” or “later life” or “mature adult*“ or “middle age*“ or “mid* life” or midlife or nonagenarian* or nursing home resident* or octogenarian* or old age* or “oldest old” or pensioner* or postmenopaus* or post-menopaus* or retired or retiree* or senior citizen* or seniors or (older adj3 adult*) or old age* or (older adj3 client*) or (older adj3 communit*) or (older adj3 female*) or (older adj3 individual*) or (older adj3 inpatient*) or (older adj3 in-patient*) or (older adj3 male*) or (older adj3 men) or (older adj3 outpatient*) or (older adj3 out-patient*) or (older adj3 patient*) or (older adj3 people) or (older adj3 person*) or (older adj3 population*) or (older adj3 women)).tw,kf. /freq=2 (422994)

3 or/1-2 (5385267)

4 *communication/ or *loneliness/ or *social isolation/ or social support/ (118907)

5 (befriend* or connectedness or (connect* adj2 other*) or (connect* adj2 people) or friendship* or interpersonal or loneliness or lonely or make friend* or making friend* or psychosocial or wellbeing or well-being).tw,kf. (256454)

6 ((enhanc* or improv* or increas* or maintain*) adj5 (communicat* or connect* or interaction*)).tw,kf. (90243)

7 ((social* adj2 contact*) or (social* adj2 connect*) or (social* adj2 distanc*) or (social* adj2 exclu*) or (social* adj2 inclu*) or (social* adj2 interact*) or (social* adj2 isolat*) or (social* adj2 participat*) or (social* adj2 relation*)).tw,kf. (75647)

8 or/4-7 (487286)

9 technology.hw. (96226)

10 *computers/ or *computer user training/ or *mobile applications/ or *social media/ or *user-computer interface/ or *wireless technology/ (57698)

11 (app or apps or audio* or chatbot* or chat forum* or chat interface* or chatroom* or chat room* or chat site* or chat software or computer* or digital* or DVD* or email* or e-mail* or electronic or facebook* or facetime or helpline* or help-line* or instant messag* or live chat* or messaging or mms or mobile app* or online* or phone* or phoning or robot* or short messag* service* or skyp* or smartphone* or sms or social network* or social media or support line* or technolog* or telephon* or texting or text messag* or tweets or twitter or video conferenc* or videoconferenc* or video record* or videorecord* or virtual* or web app* or web-based or webcam* or webcast* or web portal* or website* or web site* or “web 2.0” or “web 3.0” or whatsapp or wireless or world wide web).ti,kf. (469878)

12 (app or apps or audio* or chatbot* or chat forum* or chat interface* or chatroom* or chat room* or chat site* or chat software or computer* or digital* or DVD* or email* or e-mail* or electronic or facebook* or facetime or helpline* or help-line* or instant messag* or live chat* or messaging or mms or mobile app* or online* or phone* or phoning or robot* or short messag* service* or skyp* or smartphone* or sms or social network* or social media or support line* or technolog* or telephon* or texting or text messag* or tweets or twitter or video conferenc* or videoconferenc* or video record* or videorecord* or virtual* or web app* or web-based or webcam* or webcast* or web portal* or website* or web site* or “web 2.0” or “web 3.0” or whatsapp or wireless or world wide web).ab. /freq=2 (480103)

13 (e-counsel* or ehealth* or e-health or e-intervention* or esupport* or e-support* or etherap* or e-therap* or mhealth* or m-health or mobile health* or online CBT or remote care or remote consult* or remote* deliver* or remote health* or self-care or self-guided or self-help or telecare or tele-care or teleconsult* or tele-consult* or telehealth* or tele-health* or telegroup* or tele-group* or telemed* or tele-med* or teletherap* or tele-therap* or virtual realit*).ti,kf. (46974)

14 (e-counsel* or ehealth* or e-health or e-intervention* or esupport* or e-support* or etherap* or e-therap* or mhealth* or m-health or mobile health* or remote care or remote consult* or remote* deliver* or remote health* or self-care or self-guided or self-help or telecare or tele-care or teleconsult* or tele-consult* or telehealth* or tele-health* or telegroup* or tele-group* or telemed* or tele-med* or teletherap* or tele-therap* or virtual realit*).ab. /freq=2 (26582)

15 or/9-14 (859494)

16 (benefit* or change or changes or contribut* or decreas* or develop* or effect or effects or effectiveness or enhance* or evaluat* or experience* or experiment* or impact* or implement* or increas* or intervention* or method* or outcome* or pilot* or program* or provid* or reduc* or study or support* or system* or target* or technolog* or training or trial or “use of “).ti,kf. (9457713)

17 systematic review.mp,pt. (214838)

18 meta analysis.mp,pt. (213822)

19 (cochrane or embase or medline or pubmed).ab. (248403)

20 randomized controlled trial.pt. (529685)

21 controlled clinical trial.pt. (94148)

22 pragmatic clinical trial.pt. (1735)

23 randomi*.tw,kf. (688878)

24 placebo.ab. (218033)

25 clinical trials as topic/ (195808)

26 (randomly adj2 (allocated or assigned)).ab. (149132)

27 trial.ti. (239911)

28 (group or groups).ab. /freq=2 (1953207)

29 ((quasi experiment* or quasiexperiment* or quasi randomi* or quasirandomi*) adj2 (design* or method* or study or trial)).ab,kf. (11681)

30 ((before adj5 after) or (controlled adj3 study) or (controlled adj3 trial) or control group* or effect? or evaluat* or experience* or impact? or intervention* or (pre adj5 post) or ((pretest or pre-test) and (posttest or post test))).ab,kf. /freq=2 (4635358)

31 controlled before-after studies/ (612)

32 (controlled before* adj2 after).ab,kf. (1116)

33 interrupted time series analysis/ (1215)

34 (time series adj5 (analys* or design* or interrupted or ITS or studies or study or trial)).ab,kf. (13874)

35 or/17-34 (6471051)

36 animals/ not (humans/ and animals/) (4791745)

37 35 not 36 (5374053)

38 3 and 8 and 15 and 16 and 37 (4685)

--------------------------------------------------------------------------------

**Database: Embase Classic+Embase <1947 to 2021 May 14>**

Search Date: 16 May 2021

Yield: 4003

--------------------------------------------------------------------------------

1 exp aged/ or middle aged/ (4423286)

2 (ageing or aging or care home resident* or community-dwelling or elder* or frail* or geriatric* or “late life” or “later life” or “mature adult*“ or “middle age*“ or “mid* life” or midlife or nonagenarian* or nursing home resident* or octogenarian* or old age* or “oldest old” or pensioner* or postmenopaus* or post-menopaus* or retired or retiree* or senior citizen* or seniors or (older adj3 adult*) or old age* or (older adj3 client*) or (older adj3 communit*) or (older adj3 female*) or (older adj3 individual*) or (older adj3 inpatient*) or (older adj3 in-patient*) or (older adj3 male*) or (older adj3 men) or (older adj3 outpatient*) or (older adj3 out-patient*) or (older adj3 patient*) or (older adj3 people) or (older adj3 person*) or (older adj3 population*) or (older adj3 women)).tw. /freq=2 (568588)

3 or/1-2 (4621214)

4 *communication/ or *loneliness/ or *social isolation/ or social support/ (146374)

5 (befriend* or connectedness or (connect* adj2 other*) or (connect* adj2 people) or friendship* or interpersonal or loneliness or lonely or make friend* or making friend* or psychosocial or wellbeing or well-being).tw. (328973)

6 ((enhanc* or improv* or increas* or maintain*) adj5 (communicat* or connect* or interaction*)).tw. (116475)

7 ((social* adj2 contact*) or (social* adj2 connect*) or (social* adj2 distanc*) or (social* adj2 exclu*) or (social* adj2 inclu*) or (social* adj2 interact*) or (social* adj2 isolat*) or (social* adj2 participat*) or (social* adj2 relation*)).tw. (93277)

8 or/4-7 (619385)

9 technology.hw. (198580)

10 exp *computers/ or *human computer interaction/ or exp *mobile application/ or *social media/ or *computer interface/ or *wireless communication/ (92346)

11 (app or apps or audio* or chatbot* or chat forum* or chat interface* or chatroom* or chat room* or chat site* or chat software or computer* or digital* or DVD* or email* or e-mail* or electronic or facebook* or facetime or helpline* or help-line* or instant messag* or live chat* or messaging or mms or mobile app* or online* or phone* or phoning or robot* or short messag* service* or skyp* or smartphone* or sms or social network* or social media or support line* or technolog* or telephon* or texting or text messag* or tweets or twitter or video conferenc* or videoconferenc* or video record* or videorecord* or virtual* or web app* or web-based or webcam* or webcast* or web portal* or website* or web site* or “web 2.0” or “web 3.0” or whatsapp or wireless or world wide web).ti. (499318)

12 (app or apps or audio* or chatbot* or chat forum* or chat interface* or chatroom* or chat room* or chat site* or chat software or computer* or digital* or DVD* or email* or e-mail* or electronic or facebook* or facetime or helpline* or help-line* or instant messag* or live chat* or messaging or mms or mobile app* or online* or phone* or phoning or robot* or short messag* service* or skyp* or smartphone* or sms or social network* or social media or support line* or technolog* or telephon* or texting or text messag* or tweets or twitter or video conferenc* or videoconferenc* or video record* or videorecord* or virtual* or web app* or web-based or webcam* or webcast* or web portal* or website* or web site* or “web 2.0” or “web 3.0” or whatsapp or wireless or world wide web).ab. /freq=2 (644410)

13 (e-counsel* or ehealth* or e-health or e-intervention* or esupport* or e-support* or etherap* or e-therap* or mhealth* or m-health or mobile health* or online CBT or remote care or remote consult* or remote* deliver* or remote health* or self-care or self-guided or self-help or telecare or tele-care or teleconsult* or tele-consult* or telehealth* or tele-health* or telegroup* or tele-group* or telemed* or tele-med* or teletherap* or tele-therap* or virtual realit*).ti. (39628)

14 (e-counsel* or ehealth* or e-health or e-intervention* or esupport* or e-support* or etherap* or e-therap* or mhealth* or m-health or mobile health* or online CBT or remote care or remote consult* or remote* deliver* or remote health* or self-care or self-guided or self-help or telecare or tele-care or teleconsult* or tele-consult* or telehealth* or tele-health* or telegroup* or tele-group* or telemed* or tele-med* or teletherap* or tele-therap* or virtual realit*).ab. /freq=2 (34397)

15 or/9-14 (1089295)

16 (benefit* or change or changes or contribut* or decreas* or develop* or effect or effects or effectiveness or enhance* or evaluat* or experience* or experiment* or impact* or implement* or increas* or intervention* or method* or outcome* or pilot* or program* or provid* or reduc* or study or support* or system* or target* or technolog* or training or trial or “use of “).ti. (11478419)

17 systematic review.tw. (228178)

18 meta analysis*.tw. (222815)

19 (cochrane or embase or medline or pubmed).ab. (314793)

20 randomized controlled trial/ (658114)

21 crossover procedure/ (67193)

22 double-blind procedure/ (186270)

23 randomi*.tw. (987166)

24 (crossover* or cross-over*).tw. (113780)

25 placebo.ab. (319158)

26 ((doubl* or singl*) adj blind*).tw. (250056)

27 (randomly adj2 (allocated or assigned)).ab. (188107)

28 (group or groups).ab. /freq=2 (2908853)

29 ((quasi experiment* or quasiexperiment* or quasi randomi* or quasirandomi*) adj2 (design* or method* or study or trial)).ab. (14180)

30 ((before adj5 after) or (controlled adj3 study) or (controlled adj3 trial) or control group* or effect? or evaluat* or experience* or impact? or intervention* or (pre adj5 post) or ((pretest or pre-test) and (posttest or post test))).ab. /freq=2 (6397976)

31 (controlled before* adj2 after).ab. (1257)

32 time series analysis/ (29066)

33 (time series adj5 (analys* or design* or interrupted or ITS or studies or study or trial)).ab. (15610)

34 or/17-33 (8761701)

35 (exp animal/ or animal.hw. or nonhuman/) not (exp human/ or human cell/ or (human or humans).ti.) (7392778)

36 34 not 35 (7091401)

37 3 and 8 and 15 and 16 and 36 (4003)

--------------------------------------------------------------------------------

**Database: APA PsycInfo <1806 to May Week 2 2021> via Ovid**

Search Date: 16 May 2021

Yield: 1214

--------------------------------------------------------------------------------

1 aged/ or exp aging/ or older adulthood/ or geriatrics/ or nursing home residents/ or middle adulthood/ (98247)

2 (ageing or aging or care home resident* or community-dwelling or elder* or frail* or geriatric* or “late life” or “later life” or “mature adult*“ or “middle age*“ or “mid* life” or midlife or nonagenarian* or nursing home resident* or octogenarian* or old age* or “oldest old” or pensioner* or postmenopaus* or post-menopaus* or retired or retiree* or senior citizen* or seniors or (older adj3 adult*) or old age* or (older adj3 client*) or (older adj3 communit*) or (older adj3 female*) or (older adj3 individual*) or (older adj3 inpatient*) or (older adj3 in-patient*) or (older adj3 male*) or (older adj3 men) or (older adj3 outpatient*) or (older adj3 out-patient*) or (older adj3 patient*) or (older adj3 people) or (older adj3 person*) or (older adj3 population*) or (older adj3 women)).tw. (235261)

3 or/1-2 (248253)

4 exp communication/ or loneliness/ or social isolation/ or social support/ or friendship/ or social interaction/ (393035)

5 (befriend* or connectedness or (connect* adj2 other*) or (connect* adj2 people) or friendship* or interpersonal or loneliness or lonely or make friend* or making friend* or psychosocial or wellbeing or well-being).tw. (306929)

6 ((enhanc* or improv* or increas* or maintain*) adj5 (communicat* or connect* or interaction*)).tw. (36081)

7 ((social* adj2 contact*) or (social* adj2 connect*) or (social* adj2 distanc*) or (social* adj2 exclu*) or (social* adj2 inclu*) or (social* adj2 interact*) or (social* adj2 isolat*) or (social* adj2 participat*) or (social* adj2 relation*)).tw. (113124)

8 or/4-7 (740119)

9 technology.hw. (53981)

10 exp *computers/ or exp digital technology/ or exp electronic communication/ or exp human computer interaction/ or wireless technologies/ (157495)

11 (app or apps or audio* or chatbot* or chat forum* or chat interface* or chatroom* or chat room* or chat site* or chat software or computer* or digital* or DVD* or email* or e-mail* or electronic or facebook* or facetime or helpline* or help-line* or instant messag* or live chat* or messaging or mms or mobile app* or online* or phone* or phoning or robot* or short messag* service* or skyp* or smartphone* or sms or social network* or social media or support line* or technolog* or telephon* or texting or text messag* or tweets or twitter or video conferenc* or videoconferenc* or video record* or videorecord* or virtual* or web app* or web-based or webcam* or webcast* or web portal* or website* or web site* or “web 2.0” or “web 3.0” or whatsapp or wireless or world wide web).ti. (133134)

12 (app or apps or audio* or chatbot* or chat forum* or chat interface* or chatroom* or chat room* or chat site* or chat software or computer* or digital* or DVD* or email* or e-mail* or electronic or facebook* or facetime or helpline* or help-line* or instant messag* or live chat* or messaging or mms or mobile app* or online* or phone* or phoning or robot* or short messag* service* or skyp* or smartphone* or sms or social network* or social media or support line* or technolog* or telephon* or texting or text messag* or tweets or twitter or video conferenc* or videoconferenc* or video record* or videorecord* or virtual* or web app* or web-based or webcam* or webcast* or web portal* or website* or web site* or “web 2.0” or “web 3.0” or whatsapp or wireless or world wide web).ab. /freq=2 (193359)

13 (e-counsel* or ehealth* or e-health or e-intervention* or esupport* or e-support* or etherap* or e-therap* or mhealth* or m-health or mobile health* or online CBT or remote care or remote consult* or remote* deliver* or remote health* or self-care or self-guided or self-help or telecare or tele-care or teleconsult* or tele-consult* or telehealth* or tele-health* or telegroup* or tele-group* or telemed* or tele-med* or teletherap* or tele-therap* or virtual realit*).tw. (35201)

14 or/9-13 (324427)

15 (benefit* or change or changes or contribut* or decreas* or develop* or effect or effects or effectiveness or enhance* or evaluat* or experience* or experiment* or impact* or implement* or increas* or intervention* or method* or outcome* or pilot* or program* or provid* or reduc* or study or support* or system* or target* or technolog* or training or trial or “use of “).ti. (1699614)

16 “systematic review”/ (602)

17 systematic review.md,tw. (38916)

18 meta analysis/ (5007)

19 (meta-analys* or metaanalys*).md,tw. (41526)

20 (cochrane or embase or medline or pubmed).ab. (27492)

21 exp randomized controlled trials/ (948)

22 randomi*.tw. (93517)

23 placebo.ab. (40571)

24 ((doubl* or singl*) adj blind*).tw. (26918)

25 (randomly adj2 (allocated or assigned)).ab. (42958)

26 trial.ti. (32761)

27 (group or groups).ab. (896743)

28 ((quasi experiment* or quasiexperiment* or quasi randomi* or quasirandomi*) adj2 (design* or method* or study or trial)).ab. (8935)

29 ((before adj5 after) or (controlled adj3 study) or (controlled adj3 trial) or control group* or effect? or evaluat* or experience* or impact? or intervention* or (pre adj5 post) or ((pretest or pre-test) and (posttest or post test))).ab. /freq=2 (1158826)

30 (controlled before* adj2 after).tw. (120)

31 time series/ (2321)

32 (time series adj5 (analys* or design* or interrupted or ITS or studies or study or trial)).tw. (4361)

33 or/16-32 (1828308)

34 3 and 8 and 14 and 15 and 33 (1214)

--------------------------------------------------------------------------------

**Database: CINAHL via EBSCO**

Search Date: 16 May 2021

Yield: 1704

--------------------------------------------------------------------------------

S33 S32 Limiters - Exclude MEDLINE records 1,704

S32 S3 AND S8 AND S13 AND S14 AND S31 3,725

S31 (S29 NOT S30) 2,424,463

S30 ( (MH (animal studies) OR MH animals+ OR TI (animal model* OR mouse model* OR murine model* OR rat model*)) NOT MH (human)) 194,541

S29 (S15 OR S16 OR S17 OR S18 OR S19 OR S20 OR S21 OR S22 OR S23 OR S24 OR S25 OR S26 OR S27 OR S28) 2,533,612

S28 TI ( (time series N5 (analys* OR design* OR interrupted OR ITS OR studies OR study OR trial))) OR AB ( (time series N5 (analys* OR design* OR interrupted OR ITS OR studies OR study OR trial))) 4,680

S27 MH (“Interrupted Time Series Analysis”) 575

S26 TI (controlled before* N2 after) OR AB (controlled before* N2 after) 852

S25 MH (“Controlled Before-After Studies”) 198

S24 AB ((before N5 after) OR (controlled N3 study) OR (controlled N3 trial) OR control group* OR effect? OR evaluat* OR experience* OR impact* OR intervention* OR (pre N5 post) OR ((pretest OR pre-test) and (posttest OR post test))) 1,911,746

S23 TI ( ((quasi experimental OR quasiexperimental OR quasi randomi* OR quasirandomi*) N2 (design* OR method* OR study OR trial))) OR AB ( ((quasi experimental OR quasiexperimental OR quasi randomi* OR quasirandomi*) N2 (design* OR method* OR study OR trial))) 12,524

S22 TI ( ((singl* N1 blind*) OR (doubl* N1 blind*))) OR AB ( ((singl* N1 blind*) OR (doubl* N1 blind*))) 50,223

S21 TI (intervention* OR trial) 207,899

S20 AB (allocat* OR assign* OR control* OR random*) 823,932

S19 (MH “Randomized Controlled Trials”) OR (MH “Single-Blind Studies”)OR (MH “Double-Blind Studies”) OR (MH “Triple-Blind Studies”) OR (MH “Random Assignment”) OR MH (“Intervention Trials”) OR MH (“Crossover Design”) OR MH (“Cluster Sample”) OR MH (“Placebos”) 204,576

S18 PT randomized controlled trial 128,144

S17 AB (cochrane OR embase OR medline OR pubmed OR searched OR searches OR group OR groups) 895,315

S16 TI ( (systematic N2 review) OR TI meta analy* OR TI metaanaly*) OR AB ( (systematic N2 review) OR TI meta analy* OR TI metaanaly*) 135,673

S15 (MH “Systematic Review”) OR (MH “Meta Analysis”) 119,987

S14 TI (benefit* OR change OR changes OR contribut* OR decreas* OR develop* OR effect OR effects OR effectiveness OR enhance* OR evaluat* OR experience* OR experiment* OR impact* OR implement* OR increas* OR intervention* OR method* OR outcome* OR pilot* OR program* OR provid* OR reduc* OR study OR support* OR system* OR target* OR technolog* OR training OR trial OR “use of “) 2,237,094

S13 (S9 OR S10 OR S11 OR S12) 269,987

S12 TI ( (e-counsel* OR ehealth* OR e-health OR e-intervention* OR esupport* OR e-support* OR etherap* OR e-therap* OR mhealth* OR m-health OR mobile health* OR online CBT OR remote care OR remote consult* OR remote* deliver* OR remote health* OR self-care OR self-guided OR self-help OR telecare OR tele-care OR teleconsult* OR tele-consult* OR telehealth* OR tele-health* OR telegroup* OR tele-group* OR telemed* OR tele-med* OR teletherap* OR tele-therap* OR virtual realit*)) OR AB ( (e-counsel* OR ehealth* OR e-health OR e-intervention* OR esupport* OR e-support* OR etherap* OR e-therap* OR mhealth* OR m-health OR mobile health* OR online CBT OR remote care OR remote consult* OR remote* deliver* OR remote health* OR self-care OR self-guided OR self-help OR telecare OR tele-care OR teleconsult* OR tele-consult* OR telehealth* OR tele-health* OR telegroup* OR tele-group* OR telemed* OR tele-med* OR teletherap* OR tele-therap* OR virtual realit*)) 47,907

S11 TI ( (app OR apps OR audio* OR chatbot* OR chat forum* OR chat interface* OR chatroom* OR chat room* OR chat site* OR chat software OR computer* OR digital* OR DVD* OR email* OR e-mail* OR electronic OR facebook* OR facetime OR helpline* OR help-line* OR instant messag* OR live chat* OR messaging OR mms OR mobile app* OR online* OR phone* OR phoning OR robot* OR short messag* service* OR skyp* OR smartphone* OR sms OR social network* OR social media OR support line* OR technolog* OR telephon* OR texting OR text messag* OR tweets OR twitter OR video conferenc* OR videoconferenc* OR video record* OR videorecord* OR virtual* OR web app* OR web-based OR webcam* OR webcast* OR web portal* OR website* OR web site* OR “web 2.0” OR “web 3.0” OR whatsapp OR wireless OR world wide web)) AND AB ( (app OR apps OR audio* OR chatbot* OR chat forum* OR chat interface* OR chatroom* OR chat room* OR chat site* OR chat software OR computer* OR digital* OR DVD* OR email* OR e-mail* OR electronic OR facebook* OR facetime OR helpline* OR help-line* OR instant messag* OR live chat* OR messaging OR mms OR mobile app* OR online* OR phone* OR phoning OR robot* OR short messag* service* OR skyp* OR smartphone* OR sms OR social network* OR social media OR support line* OR technolog* OR telephon* OR texting OR text messag* OR tweets OR twitter OR video conferenc* OR videoconferenc* OR video record* OR videorecord* OR virtual* OR web app* OR web-based OR webcam* OR webcast* OR web portal* OR website* OR web site* OR “web 2.0” OR “web 3.0” OR whatsapp OR wireless OR world wide web)) 89,765

S10 (MM “Computers and Computerization”) OR (MM “Computer Environment+“) OR (MM “Computer Systems+“) OR (MM “Mobile Applications”) OR (MM “Telecommunications+“) OR (MH “Therapy, Computer Assisted”) 120,310

S9 MW technology 73,772

S8 (S4 OR S5 OR S6 OR S7) 367,212

S7 TI ( ((social* N2 contact*) OR (social* N2 connect*) OR (social* N2 distanc*) OR (social* N2 exclu*) OR (social* N2 inclu*) OR (social* N2 interact*) OR (social* N2 isolat*) OR (social* N2 participat*) OR (social* N2 relation*))) OR AB ( ((social* N2 contact*) OR (social* N2 connect*) OR (social* N2 distanc*) OR (social* N2 exclu*) OR (social* N2 inclu*) OR (social* N2 interact*) OR (social* N2 isolat*) OR (social* N2 participat*) OR (social* N2 relation*))) 42,336

S6 TI ( ((enhanc* OR improv* OR increas* OR maintain*) N5 (communicat* OR connect* OR interaction*))) OR AB ( ((enhanc* OR improv* OR increas* OR maintain*) N5 (communicat* OR connect* OR interaction*))) 28,433

S5 TI ( (befriend* OR connectedness OR (connect* N2 other*) OR (connect* N2 people) OR friendship* OR interpersonal OR loneliness OR lonely OR make friend* OR making friend* OR psychosocial OR wellbeing OR well-being)) OR AB ( (befriend* OR connectedness OR (connect* N2 other*) OR (connect* N2 people) OR friendship* OR interpersonal OR loneliness OR lonely OR make friend* OR making friend* OR psychosocial OR wellbeing OR well-being)) 142,617

S4 (MH Communication) OR (MH “Social Isolation+“) OR (MH “Support, Psychosocial”) OR (MH “Interpersonal Relations”) OR (MH “Friendship”) OR (MH “Social Integration”) 215,242

S3 (S1 OR S2) 1,448,300

S2 TI ( (ageing OR aging OR care home resident* OR centenarian* OR community-dwelling OR elder* OR frail* OR geriatric* OR “late life” OR “later life” OR “mature adult*“ OR “middle age*“ OR “mid* life” OR midlife OR nonagenarian* OR nursing home resident* OR octogenarian* OR old age* OR “oldest old” OR pensioner* OR postmenopaus* OR post-menopaus* OR retired OR retiree* OR senior* OR septuagenarian OR sexagenarian* OR veteran*)) OR AB ( (ageing OR aging OR care home resident* OR centenarian* OR community-dwelling OR elder* OR frail* OR geriatric* OR “late life” OR “later life” OR “mature adult*“ OR “middle age*“ OR “mid* life” OR midlife OR nonagenarian* OR nursing home resident* OR octogenarian* OR old age* OR “oldest old” OR pensioner* OR postmenopaus* OR post-menopaus* OR retired OR retiree* OR senior* OR septuagenarian OR sexagenarian* OR veteran*)) 300,074

S1 (MH “Aged+“) OR (MH “Middle Age”) 1,326,993

--------------------------------------------------------------------------------

**Database: Web of Science via Clarivate (Indexes=SCI-EXPANDED, SSCI, CPCI-S, CPCI-SSH, ESCI)** Timespan=All years

Search Date: 17 May 2021

Yield: 853

--------------------------------------------------------------------------------

#8 853 #7 AND #6 AND #5 AND #4

#7 2,072,148 TS=(controlled before-after OR “controlled trial” OR “doubl* blind*“ OR “meta-analy*“ OR “metaanaly*“ OR randomi* OR randomly OR “singl* blind*“ OR “systematic review” OR “time series”)

#6 1,917,703 TI=(app OR apps OR audio* OR chatbot* OR “chat forum*“ OR “chat interface*“ OR chatroom* OR “chat room*“ OR “chat site*“ OR “chat software” OR computer* OR digital* OR DVD* OR “e-counsel*“ OR ehealth* OR “e-health” OR “e-intervention*“ OR email* OR e-mail* OR electronic OR esupport* OR “e-support*“ OR etherap* OR “e-therap*“ OR facebook* OR facetime OR helpline* OR “help line*“ OR “instant messag*“ OR “live chat*“ OR mhealth* OR “m-health” OR “mobile health*“ OR messaging OR mms OR “mobile app*“ OR online* OR phone* OR phoning OR “remote care” OR “remote consult*“ OR “remote* deliver*“ OR “remote health*“ OR robot* OR “self-care” OR “self-guided” OR “self-help” OR “short messag* service*“ OR skyp* OR smartphone* OR sms OR “social network*“ OR “social media” OR “support line*“ OR technolog* OR telecare OR “tele-care” OR teleconsult* OR “tele-consult*“ OR telehealth* OR “tele-health*“ OR telegroup* OR “tele-group*“ OR telemed* OR “tele-med*“ OR telephon* OR teletherap* OR “tele-therap*“ OR texting OR “text messag” OR tweets OR twitter OR “video conferenc*“ OR videoconferenc* OR virtual* OR “web app*“ OR “web-based” OR webcam* OR webcast* OR “web portal*“ OR website* OR “web site*“ OR “web 2.0” OR “web 3.0” OR whatsapp OR wireless OR “world wide web”)

#5 762,426 TI=(befriend* OR connected* OR loneliness OR lonely OR psychosocial* OR “social isolation” OR “social support*“ OR “socially isolated” OR “well-being” OR wellbeing) OR AB=(befriend* OR connected* OR loneliness OR lonely OR psychosocial* OR “social isolation” OR “social support*“ OR “socially isolated” OR “well-being” OR wellbeing)

#4 4,257,374 #3 OR #2 OR #1

#3 1,062,970 TI=((older NEAR/3 adult*) OR (older NEAR/3 client*) OR (older NEAR/3 communit*) OR (older NEAR/3 female*) OR (older NEAR/3 individual*) OR (older NEAR/3 inpatient*) OR (older NEAR/3 in-patient*) OR (older NEAR/3 male*) OR (older NEAR/3 men) OR (older NEAR/3 outpatient*) OR (older NEAR/3 out-patient*) OR (older NEAR/3 patient*) OR (older NEAR/3 people) OR (older NEAR/3 person*) OR (older NEAR/3 population*) OR (older NEAR/3 women)) OR AB=((older NEAR/3 adult*) OR (older NEAR/3 client*) OR (older NEAR/3 communit*) OR (older NEAR/3 female*) OR (older NEAR/3 individual*) OR (older NEAR/3 inpatient*) OR (older NEAR/3 in-patient*) OR (older NEAR/3 male*) OR (older NEAR/3 men) OR (older NEAR/3 outpatient*) OR (older NEAR/3 out-patient*) OR (older NEAR/3 patient*) OR (older NEAR/3 people) OR (older NEAR/3 person*) OR (older NEAR/3 population*) OR (older NEAR/3 women) OR (old age))

#2 302.219 SU=(aged OR ageing OR aging OR elder* OR geriatric* OR middle age OR older OR senior*)

#1 3,686,745 TI=(ageing OR aging OR “care home resident*“ OR centenarian* OR “community-dwelling” OR elder* OR frail* OR geriatric* OR “late life” OR “later life” OR “mature adult*“ OR “middle age*“ OR “mid* life” OR midlife OR nonagenarian* OR “nursing home resident*“ OR octogenarian* OR “old age*“ OR “oldest old” OR pensioner* OR postmenopaus* OR post-menopaus* OR retired OR retiree* OR senior* OR septuagenarian OR sexagenarian* OR veteran*) OR AB=(aged OR ageing OR aging OR “care home resident*“ OR centenarian* OR “community-dwelling” OR elder* OR frail* OR geriatric* OR “late life” OR “later life” OR “mature adult*“ OR “middle age*“ OR “mid* life” OR midlife OR nonagenarian* OR “nursing home resident*“ OR octogenarian* OR “old age*“ OR “oldest old” OR pensioner* OR postmenopaus* OR post-menopaus* OR retired OR retiree* OR senior* OR septuagenarian OR sexagenarian* OR veteran*)

--------------------------------------------------------------------------------

**Database: ProQuest (all databases)**

Search Date: 17 May 2021

Yield: 706

--------------------------------------------------------------------------------

ti((“care home resident*“ OR “community-dwelling” OR elder* OR frail OR geriatric* OR “late life” OR “later life” OR “middle-age*“ OR mid-life OR midlife OR “nursing home resident*“ OR “old age*“ OR older OR pensioner* OR retired OR retiree* OR “senior citizen*“ OR seniors)) AND (controlled before-after OR controlled trial OR “meta-analysis” OR randomi* OR randomly OR “systematic review” OR “time series”) AND ti((befriend* OR connected* OR loneliness OR lonely OR psychosocial* OR “social isolation” OR “social support*“ OR “socially isolated” OR “well-being” OR wellbeing)) AND noft((app OR apps OR audio* OR chatbot* OR chat forum* OR chat interface* OR chatroom* OR “chat room*“ OR chat site* OR chat software OR computer* OR digital* OR DVD* OR email* OR e-mail* OR electronic OR facebook* OR facetime OR helpline* OR help-line* OR instant messag* OR live chat* OR messaging OR mms OR mobile app* OR online* OR phone* OR phoning OR robot* OR short messag* service* OR skyp* OR smartphone* OR sms OR social network* OR social media OR support line* OR technolog* OR telephon* OR texting OR text messag* OR tweets OR twitter OR video conferenc* OR videoconferenc* OR video record* OR videorecord* OR virtual* OR web app* OR web-based OR webcam* OR webcast* OR “web portal*“ OR website* OR “web site*“ OR “web 2.0” OR “web 3.0” OR whatsapp OR wireless OR “world wide web”))

--------------------------------------------------------------------------------

**Database: International Bibliography of the Social Sciences (IBSS) via ProQuest**

Search Date: 17 May 2021

Yield: 275

--------------------------------------------------------------------------------

noft((“care home resident*“ OR “community-dwelling” OR elder* OR frail OR geriatric* OR “late life” OR “later life” OR “middle-age*“ OR mid-life OR midlife OR “nursing home resident*“ OR “old age*“ OR older OR pensioner* OR retired OR retiree* OR “senior citizen*“ OR seniors)) AND noft((app OR apps OR audio* OR chatbot* OR chat forum* OR chat interface* OR chatroom* OR “chat room*“ OR chat site* OR chat software OR computer* OR digital* OR DVD* OR email* OR e-mail* OR electronic OR facebook* OR facetime OR helpline* OR help-line* OR instant messag* OR live chat* OR messaging OR mms OR mobile app* OR online* OR phone* OR phoning OR robot* OR short messag* service* OR skyp* OR smartphone* OR sms OR social network* OR social media OR support line* OR technolog* OR telephon* OR texting OR text messag* OR tweets OR twitter OR video conferenc* OR videoconferenc* OR video record* OR videorecord* OR virtual* OR web app* OR web-based OR webcam* OR webcast* OR “web portal*“ OR website* OR “web site*“ OR “web 2.0” OR “web 3.0” OR whatsapp OR wireless OR “world wide web”)) AND noft((befriend* OR connected* OR loneliness OR lonely OR psychosocial* OR “social isolation” OR “social support*“ OR “socially isolated” OR “well-being” OR wellbeing)) AND (controlled before-after OR controlled trial OR “meta-analysis” OR randomi* OR randomly OR “systematic review” OR “time series”)

--------------------------------------------------------------------------------

**Database: EBSCO (all databases except CINAHL)**

Search Date: 17 May 2021

Yield: 578

--------------------------------------------------------------------------------

TX ( (“care home resident*“ OR “community-dwelling” OR elder* OR frail OR geriatric* OR “late life” OR “later life” OR “middle-age*“ OR mid-life OR midlife OR “nursing home resident*“ OR “old age*“ OR older OR pensioner* OR retired OR retiree* OR “senior citizen*“ OR seniors)) AND TI ( (befriend* OR connected* OR loneliness OR lonely OR psychosocial* OR “social isolation” OR “social support*“ OR “socially isolated” OR “well-being” OR wellbeing)) AND TI ( (app OR apps OR audio* OR chatbot* OR chat forum* OR chat interface* OR chatroom* OR “chat room*“ OR chat site* OR chat software OR computer* OR digital* OR DVD* OR email* OR e-mail* OR electronic OR facebook* OR facetime OR helpline* OR help-line* OR instant messag* OR live chat* OR messaging OR mms OR mobile app* OR online* OR phone* OR phoning OR robot* OR short messag* service* OR skyp* OR smartphone* OR sms OR social network* OR social media OR support line* OR technolog* OR telephon* OR texting OR text messag* OR tweets OR twitter OR video conferenc* OR videoconferenc* OR video record* OR videorecord* OR virtual* OR web app* OR web-based OR webcam* OR webcast* OR “web portal*“ OR website* OR “web site*“ OR “web 2.0” OR “web 3.0” OR whatsapp OR wireless OR “world wide web”)) AND TX ( (controlled before-after OR controlled trial OR “meta-analysis” OR randomi* OR randomly OR “systematic review” OR “time series”))

--------------------------------------------------------------------------------

**Database: Global Index Medicus**

Search Date: 17 May 2021

Yield: 213

--------------------------------------------------------------------------------

tw:((tw:((“care home resident*“ OR “community-dwelling” OR elder* OR frail OR geriatric* OR “late life” OR “later life” OR “middle-age*“ OR mid-life OR midlife OR “nursing home resident*“ OR “old age*“ OR older OR pensioner* OR retired OR retiree* OR “senior citizen*“ OR seniors))) AND (tw:((befriend* OR connected* OR loneliness OR lonely OR psychosocial* OR “social isolation” OR “social support*“ OR “socially isolated” OR “well-being” OR wellbeing))) AND (ti:((app OR apps OR audio* OR chatbot* OR “chat forum*“ OR “chat interface*“ OR chatroom* OR “chat room*“ OR “chat site*“ OR “chat software” OR computer* OR digital* OR dvd* OR “e-counsel*“ OR ehealth* OR “e-health” OR “e-intervention*“ OR email* OR e-mail* OR electronic OR esupport* OR “e-support*“ OR etherap* OR “e-therap*“ OR facebook* OR facetime OR helpline* OR “help line*“ OR “instant messag*“ OR “live chat*“ OR mhealth* OR “m-health” OR “mobile health*“ OR messaging OR mms OR “mobile app*“ OR online* OR phone* OR phoning OR “remote care” OR “remote consult*“ OR “remote* deliver*“ OR “remote health*“ OR robot* OR “self-care” OR “self-guided” OR “self-help” OR “short messag* service*“ OR skyp* OR smartphone* OR sms OR “social network*“ OR “social media” OR “support line*“ OR technolog* OR telecare OR “tele-care” OR teleconsult* OR “tele-consult*“ OR telehealth* OR “tele-health*“ OR telegroup* OR “tele-group*“ OR telemed* OR “tele-med*“ OR telephon* OR teletherap* OR “tele-therap*“ OR texting OR “text messag” OR tweets OR twitter OR “video conferenc*“ OR videoconferenc* OR virtual* OR “web app*“ OR “web-based” OR webcam* OR webcast* OR “web portal*“ OR website* OR “web site*“ OR “web 2.0” OR “web 3.0” OR whatsapp OR wireless OR “world wide web”))))

--------------------------------------------------------------------------------

**Database: Epistemonikos**

Search Date: 17 May 2021

Yield: 1093

--------------------------------------------------------------------------------

(title:((ageing OR aging OR “care home resident*“ OR “community-dwelling” OR elder* OR frail OR geriatric* OR “late life” OR “later life” OR “middle-age*“ OR mid-life OR midlife OR “nursing home resident*“ OR “old age*“ OR older OR pensioner* OR retired OR retiree* OR “senior citizen*“ OR seniors)) OR abstract:((ageing OR aging OR “care home resident*“ OR “community-dwelling” OR elder* OR frail OR geriatric* OR “late life” OR “later life” OR “middle-age*“ OR mid-life OR midlife OR “nursing home resident*“ OR “old age*“ OR older OR pensioner* OR retired OR retiree* OR “senior citizen*“ OR seniors))) AND (title:((befriend* OR connected* OR loneliness OR lonely OR psychosocial* OR “social isolation” OR “social support*“ OR “socially isolated” OR “well-being” OR wellbeing)) OR abstract:((befriend* OR connected* OR loneliness OR lonely OR psychosocial* OR “social isolation” OR “social support*“ OR “socially isolated” OR “well-being” OR wellbeing))) AND (title:((app OR apps OR audio* OR chatbot* OR “chat forum*“ OR “chat interface*“ OR chatroom* OR “chat room*“ OR “chat site*“ OR “chat software” OR computer* OR digital* OR DVD* OR “e-counsel*“ OR ehealth* OR “e-health” OR “e-intervention*“ OR email* OR e-mail* OR electronic OR esupport* OR “e-support*“ OR etherap* OR “e-therap*“ OR facebook* OR facetime OR helpline* OR “help line*“ OR “instant messag*“ OR “live chat*“ OR mhealth* OR “m-health” OR “mobile health*“ OR messaging OR mms OR “mobile app*“ OR online* OR phone* OR phoning OR “remote care” OR “remote consult*“ OR “remote* deliver*“ OR “remote health*“ OR robot* OR “self-care” OR “self-guided” OR “self-help” OR “short messag* service*“ OR skyp* OR smartphone* OR sms OR “social network*“ OR “social media” OR “support line*“ OR technolog* OR telecare OR “tele-care” OR teleconsult* OR “tele-consult*“ OR telehealth* OR “tele-health*“ OR telegroup* OR “tele-group*“ OR telemed* OR “tele-med*“ OR telephon* OR teletherap* OR “tele-therap*“ OR texting OR “text messag” OR tweets OR twitter OR “video conferenc*“ OR videoconferenc* OR virtual* OR “web app*“ OR “web-based” OR webcam* OR webcast* OR “web portal*“ OR website* OR “web site*“ OR “web 2.0” OR “web 3.0” OR whatsapp OR wireless OR “world wide web”)) OR abstract:((app OR apps OR audio* OR chatbot* OR “chat forum*“ OR “chat interface*“ OR chatroom* OR “chat room*“ OR “chat site*“ OR “chat software” OR computer* OR digital* OR DVD* OR “e-counsel*“ OR ehealth* OR “e-health” OR “e-intervention*“ OR email* OR e-mail* OR electronic OR esupport* OR “e-support*“ OR etherap* OR “e-therap*“ OR facebook* OR facetime OR helpline* OR “help line*“ OR “instant messag*“ OR “live chat*“ OR mhealth* OR “m-health” OR “mobile health*“ OR messaging OR mms OR “mobile app*“ OR online* OR phone* OR phoning OR “remote care” OR “remote consult*“ OR “remote* deliver*“ OR “remote health*“ OR robot* OR “self-care” OR “self-guided” OR “self-help” OR “short messag* service*“ OR skyp* OR smartphone* OR sms OR “social network*“ OR “social media” OR “support line*“ OR technolog* OR telecare OR “tele-care” OR teleconsult* OR “tele-consult*“ OR telehealth* OR “tele-health*“ OR telegroup* OR “tele-group*“ OR telemed* OR “tele-med*“ OR telephon* OR teletherap* OR “tele-therap*“ OR texting OR “text messag” OR tweets OR twitter OR “video conferenc*“ OR videoconferenc* OR virtual* OR “web app*“ OR “web-based” OR webcam* OR webcast* OR “web portal*“ OR website* OR “web site*“ OR “web 2.0” OR “web 3.0” OR whatsapp OR wireless OR “world wide web”)))

Publication type: Systematic Review OR Primary Study

--------------------------------------------------------------------------------

## 3 Detailed eligibility criteria

| **Criteria** | **Inclusion** | **Exclusion** |
| --- | --- | --- |
| Population | - Older adults ≥ 60 years old  - Mixed population with disaggregated data for target population (≥ 60 years old)  - Participants in community settings (personal homes, residential homes, or independent living facilities) or supportive care institutions (nursing homes or long-term care and assisted living facilities).  -*Systematic reviews that are focused on older adults without specifying age | - Not older adults (< 60 years old)  - Mixed population without disaggregated data for target population (≥ 60 years old)  - Hospitalized patients |
| Intervention | Digital interventions for SIL  - Social skills training (e.g., computer and internet training)  - Enhancing social support (e.g., telephone befriending, telecare, robopets  - Increasing social interaction (e.g., videoconferencing, social networking sites, chat box,  - Social cognitive training (e.g., online CBT)  If a systematic review has a more general focus and looks at all intervention types, not just digital interventions, it will be included if the data is disaggregated by intervention type (shows results for digital interventions separately). | - digital interventions for monitoring (e.g., sensors for falls) and smart homes  - interventions taking place in hospital settings |
| Evidence/objective | Focus on SIL, mental health and well-being | Not focused on SIL (e.g., CBT for dementia) |
| Study design | - Systematic reviews  - Randomized controlled trials  - Quasi-experimental studies  - Controlled before-after studies  - Interrupted time series with at least six data points (3 before and 3 after a discrete intervention)  - Regression discontinuity designs  -*Studies with a comparison/control group  -protocols of systematic reviews and eligible primary studies | - Not a systematic review (e.g., literature reviews) – does not satisfy at least 4/5 criteria (i) Were inclusion/exclusion criteria reported? (ii) Was the search adequate? (iii) Were the included studies synthesized? (iv) Was the quality of the included studies assessed; (v) Are sufficient details about the individual included studies presented?  - less than six period interrupted time series design  - longitudinal cohort studies with no controls  - Cross-sectional studies  -Qualitative study designs |

## 4 Coding tool

| **Category** | **Subcategories** |
| --- | --- |
| Study characteristics | |
| Publication status | - Complete - On-going (protocol) - Conference abstracts |
| Study design | - Primary study - Systematic review |
| Primary study design | - Randomized controlled trials (RCTs) - non-randomized studies |
| Equity focus: Is the study population identified by the authors as aimed at/focused on disadvantaged across any PROGRESS factors | - Place of residence - Race/Ethnicity - Occupation - Gender/sex - Religion - Education - Socioeconomic status - Social capital (e.g. marital status, network) - Plus factor - Frailty - Plus factor - Digital literacy - Plus factor - Health status e.g. dementia, disease severity - Plus factor - Socially isolated or at risk of social isolation - Plus factor - Lonely or at risk of loneliness - Plus factor - Age - Plus factor - Disability Plus factor - Living situation (e.g. alone, long-term care) |
| If population is identified as “at risk”, how are they identified | - Case-finding - Outreach - From a community-based program - Screening in primary care |
| Methodological quality of systematic reviews | - Low or critically low quality reviews - Moderate quality reviews - High quality reviews |
| Interventions | |
| Improving social skills | - Training in how to use digital technology (e.g. computer and internet) - Digitally delivered training (e.g., about caregiving/skills building) - Digitally delivered learning (e.g., a new language) |
| Enhancing social interaction | - Digital connections with family/friends - Digital connections with community |
| Enhancing social support | - Digital/remote e-health services - Digital social and health care coordination with family/friends - Geolocating/identifying older adults who need services - Socially assistive robots and virtual pets - Virtual spaces - Virtual assistants (e.g., Google home, Alexa) - Virtual social support groups - Digital intergenerational approaches - Digital games (e.g., scrabble, chess, cards, exergames) - Digitally delivered activities (e.g., exercise - yoga, tai chi) - Digital coordination of health or social care services (e.g., online referrals) |
| Social cognitive training | - Digital cognitive behavioural therapy - Digital mindfulness training - Digital psychoeducation - Digital reminiscence therapy Digital cognitive behavioural coaching |
| Multicomponent |  |
| Intervention focus | - Loneliness - Social isolation - Social isolation and loneliness |
| Intervention format | - Group-based - One-on-one |
| How technologies are used | - Telephone calls - E-mail - Messaging - Discussion forums - Videoconferencing - Social networking sites e.g., Facebook, Twitter - Digital games - Robots and virtual companions - Virtual assistants - Virtual spaces or classrooms with messaging capabilities - Mobility tools (e.g. walky - microblogging walking frame) - Participating in an activity - Music - Unspecified |
| How training on how to use of digital technology was delivered | - Digitally (remotely) delivered training - In-person (face to face) training - Unspecified |
| Outcomes | |
| Individual | - Loneliness - Social isolation - Social connectedness - Quality of life/well-being - Anxiety/depression - Confidence level or self- esteem - Adverse effects |
| Societal | - Social support - Social engagement - Social cohesion - Social capital - Digital divide |
| Process indicators | - Acceptance - Adherence - Technology use - Feasibility - Affordability - Cost-effectiveness - Barriers |
| Population sociodemographics | |
| Sex/Gender | - Men only - Women only - Includes LGBTQ2+ |
| Age groups | - Includes 60-70 years - Includes 70-80 years - Includes >80 years - Restricted to >80 years - Unspecified |
| Health conditions | - Communicable disease - Noncommunicable disease - Dementia/Alzheimer’s disease - Depression - Comorbidity - Disability - Physical frailty - Psychological frailty - Social frailty - Care dependent - Discharge from hospital - End-of-life/palliative care |
| PROGRESS-Plus factors | - Place of residence (urban/rural or remote/unspecified) - Race/Ethnicity - Occupation - Gender or Sex (men only/women only/LGBTQ+2/unspecified) - Religion - Education - Socioeconomic status - Social capital (marital status) - Living alone - Care givers |
| Needs | - Social and emotional needs - Purpose in life - Mobility - Personal care needs - Meals - Domestic assistance - Accommodation (housing/home modifications and maintenance) - Financial management - Communication (language support/interpreters, information and assistance/referral services) - Skills development - Learning (e.g., a new activity, language or about social skills) - Care navigation support or task orientation - Clinical/health needs - Respite care - Caregiver support |
| Setting | - Personal home - Independent living (residential home) - Assisted living - Long-term care/nursing home - Unspecified |
| World Health Organization regions | - African Region - Regions of the Americas - South-East Asian Region - European Region - Eastern Mediterranean Region - Western Pacific Region - Unspecified |
| World Bank Classification by income | - Low-income economies - Lower-middle income economies - Upper-middle income economies - High-income economies - Unspecified |
| Countries | - Africa (where multiple countries) - Europe (where multiple countries) - Afghanistan - Angola - Armenia - Argentina - Australia - Austria - Bahamas, The - Bahrain - Bangladesh - Barbados - Belgium - Belarus - Belize - Bolivia - Brazil - Botswana - Bulgaria - Burkina Faso - Cambodia - Cameroon - Canada - Chile - China - Congo - Croatia - Cuba - Colombia - Cyprus - Czech Republic - Dominican Republic - Denmark - Egypt - Ecuador - Eritrea - Estonia - Finland - Ethiopia - Gambia, The - France - Georgia - Ghana - Germany - Greece - Guatemala - Guinea-Bissau - Haiti - Honduras - Hong Kong - Hungary - Iceland - India - Indonesia - Iran - Ireland - Israel - Italy - Ivory Coast - Kazakhstan - Jordan - Kenya - Korea - Kuwait - Japan - Jamaica - Lao - Latin America (where multiple countries) - Lebanon - Lesotho - Liberia - Latvia - Lithuania - Luxembourg - Macedonia - Malawi - Madagascar - Malaysia - Mali - Mexico - Micronesia - Marshall Islands - Mozambique - Mongolia - Myanmar (Burma) - Namibia - Nepal - New Zealand - The Netherlands - Nicaragua - Niger - Nigeria - Northern Ireland - Pakistan - Norway - Panama - Papua New Guinea - Peru - Philippines - Poland - Portugal - Puerto Rico - Romania - Russia - Rwanda - Samoa - San Marino - Saudi Arabia - Scotland - Serbia - Senegal - Singapore - Sierra Leone - Slovakia - Slovenia - South Africa - Spain - Sri Lanka - St Lucia - Swaziland - Sweden - Switzerland - Syria - Taiwan - Thailand - Tanzania - Trinidad and Tobago - Tunisia - Uganda - Ukraine - Turkey - UK - USA - Uzbekistan - Vanuatu - Venezuela - Vietnam - West Indies - Yemen - Zambia - Zimbabwe |
| Equity analysis: Does the study assess any differences in effects (benefit or harm) across any PROGRESS factors | - Place of residence (rural or remote/urban) - Race/ethnicity/culture/language - Occupation - Gender/sex - Religion - Education - Socioeconomic status - Social capital (e.g. social network) - Plus factor - Frailty - Plus factor - Digital literacy Plus factor - Health status (e.g. dementia, disease severity) - Plus factor - Socially isolated or at risk - Plus factor - Lonely or at risk - Plus factor - Age - Plus factor - Disability - Plus factor - Living situation (e.g. alone, long term care) |
