# Supplementary material for: Digital interventions to reduce social isolation and loneliness in older adults: An evidence and gap map
Source: Campbell Syst Rev. 2023 Nov 27;19(4):e1369. doi: 10.1002/cl2.1369 (PMC10681039; doi:10.1002/cl2.1369)
Supplement: Supplementary file 2 — Supporting information. [file CL2-19-e1369-s002.docx]

**ABBREVIATIONS AND ACRONYMS**

EGM = evidence and gap map

LGBTQIA2S+ = lesbian, gay, bisexual, transgender, queer (or sometimes questioning), intersex, asexual, and two-spirited

PICO = population, intervention, comparison, outcome

PROGRESS‐Plus = Place of residence (urban/rural), Race/ethnicity/culture and language, Occupation, Gender or sex, Religion, Occupation, Socioeconomic status, Social capital and Plus factors

WHO = World Health Organization
